# Genetic parameters, genome‐wide associations and potential candidate genes for additive and dominance effects of tail traits in Merinoland sheep based on whole‐genome sequence data in a selection experiment

**DOI:** 10.1111/age.70041

**Published:** 2025-09-18

**Authors:** Johanna Mainzer, Tong Yin, Isabella Giambra, Hannah Hümmelchen, Petra Engel, Henrik Wagner, Axel Wehrend, Sven König

**Affiliations:** ^1^ Institute of Animal Breeding and Genetics Justus‐Liebig‐University of Gießen Gießen Germany; ^2^ Zhejiang Key Laboratory of Dairy Cattle Genetic Improvement and Milk Quality Research Wenzhou China; ^3^ Veterinary Clinic for Reproductive Medicine and Neonatalogy Justus‐Liebig‐University of Gießen Gießen Germany

**Keywords:** candidate genes, dominance effects, genomic analyses, selection experiment, tail abnormalities, tail length

## Abstract

The aim of this study was an in‐depth genomic analysis for tail length (TL), tail characteristics and body measurements in the Merinoland sheep breed considering whole‐genome sequence data. Genomic analyses included the estimation of genetic parameters and dominance effects, genome‐wide associations for the additive and dominance component, and the annotation of potential candidate genes. We implemented a unified selection and mating experiment to create extreme lamb groups based on breeding values for TL. The 254 lambs from the mating experiment were phenotyped at birth for TL, tail circumference, and body length (all in cm), for body weight, and X‐rayed to count the number of vertebrae and to identify tail abnormalities for tail fractures, axis deviations, block vertebrae, and wedged vertebrae. Heritabilities using the variant‐based relationship matrix were large for the morphological measurements TL (0.85), body length (0.93), and body weight (0.85), moderate for tail circumference (0.21), and number of vertebrae (0.29), but close to zero for tail abnormalities. Dominance variance for TL explained 14.95% of the phenotypic variation, but was close to zero for the remaining tail traits. The positive breeding value correlations indicate longer and thicker tails for taller and heavier lambs. Breeding value correlations were negative between TL with block vertebrae and wedged vertebrae. Genome‐wide associations for additive‐genetic and dominance effects revealed 726 significant variants, which are located close to potential candidate genes. These candidate genes have known functions on skeletal growth, and regulate the development of bone structures and of vertebrae characteristics.

## INTRODUCTION

Long and woolly tails, as common in the majority of modern sheep breeds, imply an increased risk of faecal contamination and induce susceptibility to flystrike (e.g., Lagler et al., 2022). Tail docking in lambs is therefore part of the common practice for disease prevention in sheep, but can cause physiological and behavioural changes (Mellor & Murray, 1989). Consequently, tail docking is not in agreement with the legal animal welfare guidelines as defined in the EU Directive 2008/120/EC (European Union, 2008), suggesting establishment of sustainable alternatives. A sustainable and socially accepted solution addresses breeding approaches on shorter tails (Greeff et al., 2015; Scobie & O'Connell, 2002). In sheep as well as in other species, scientific reports indicated pronounced natural variation for tail length (TL). Quite large heritabilities for TL close to 0.60 were estimated in Merinos (Greeff et al., 2015; Oberpenning et al., 2023). TL heritabilities were also large in other sheep breeds, i.e., 0.38 in Rambouillet sheep (Shelton, 1977), 0.48 in Menz sheep (Gizaw et al., 2008), and 0.77 in Finnsheep (Branford Oltenacu & Boylan, 1974). A heritability of 0.82 was reported in F1‐crosses between short‐tailed Finnsheep and long‐tailed Cheviot sheep (Scobie & O'Connell, 2002).

A breeding focus only on TL might induce unfavourable effects on other breeding goal traits. In this regard, Oberpenning et al. (2023) estimated unfavourable genetic correlations between TL and body weight traits in Merinoland sheep. Antagonistic phenotypic correlations between TL with weights at birth and at the docking date were reported by Teubes et al. (2023) in the Elsenburg Merino. In addition to unfavourable effects on breeding goal traits, short or even ‘no‐tail’ breeding might impair physiological mechanisms and body morphology. Multiple cases of spinal defects (e.g. *spina bifida*, *perosmus elumbis*) and functional abnormalities (*arthrogryposis*, malformations of the skull and brain, *atresia ani*, *hindlimb ataxia*) have been observed in lambs of different breeds with deformed, shortened or absent tails (Potter et al., 2010). In Manx cats and in mice, inherited short tails (*brachyury*) or taillessness (*anury*) are associated with spinal defects (Herrmann et al., 1990). In White Polled Heath sheep with congenital shortening and abnormal bending of the tail, detected vertebral ankyloses (block vertebrae) and wedge‐shaped vertebra cause scoliosis of the spine (Kerkmann et al., 2010). Block vertebrae and wedged vertebrae are related with kinky tails and axis deviations of the tail (Schawalder et al., 2010). Radiographic studies examining kinks in the tails of various cat breeds indicated a combined effect of vertebral deformities, including a reduced number of caudal vertebrae and the occurrence of hemivertebrae (wedged vertebrae) (Xu et al., 2016). Hümmelchen et al. (2023) identified positive relationships between TL with the prevalence for tail fractures, axis deviations and wedged vertebrae, but a negative correlation between TL and the presence of block vertebrae.

In addition to the pronounced additive‐genetic component, dominance effects on TL and tail characteristics were proven in sheep and mice (James et al., 1991; Wu et al., 2010). James et al. (1991) assumed that the inheritance of TL in the Australian Merino depends on only a small number of interacting genes with large effects and possible dominance. These assumptions of major gene effects and of pronounced dominance are based on crossbreeding experiments with short‐tailed sheep breeds several decades ago. However, in some of these experiments, lethal effects were observed, e.g., hindquarter paralysis in F1‐lambs without tails from the cross of Siberian fat‐rumped sheep with Hampshire sheep and Rambouillet sheep (Jordan, 1952). This phenotypic phenomenon is defined as ‘sidewheeler’, mostly associated with death during early stages of life. Post‐mortem examinations of lambs confirmed abnormal termination of the spinal cord (Wilson, 1940). Consequently, lethal gene effects were postulated by Lagler et al. (2022) for genes (e.g. *TBXT*) determining short tails in mammals. In Romney sheep, Carter (1976) identified impaired viability of embryos that were homozygous carriers of some putative short‐tail alleles, e.g., for the T gene (*TBXT*) as a major candidate gene for TL. Pleiotropic effects in this regard were described by Herrmann et al. (1990), who identified abnormal mesoderm formation in mice, combined with the absence of notochord formation in embryos.

In summary, genetic effects and associations between TL with other functional and primary traits could be clearly inferred when generating F1 crosses, or when comparing phenotypically diverse sub‐groups. With regard to genomic association analyses and novel traits with limited observations for both phenotypes and genotypes, successful within‐breed approaches focussed on selective genotyping, i.e., consideration of animals with extreme phenotypes (e.g. Bagheri et al., 2013). However, for tail traits in sheep, genomic association analyses (Lagler et al., 2022; Zhi et al., 2018) considered only on a few specific genes including *HOXB13* and *TBXT*, and did not integrate whole‐genome sequences (WGS) or specific selective genotyping and phenotyping approaches.

The availability of WGS data enables association studies across the whole genome, possibly contributing to the detection of novel genomic variants or chromosomal segments of interest.

Consequently, the aim of this study was to implement a selection and mating experiment to create extreme sheep groups for TL. The established groups were used for innovative trait recording considering tail and body measurements, and X‐ray traits to detect tail abnormalities, and for whole‐genome sequencing. The combined database of innovative phenotypes and WGS was used to estimate genetic (co)variance components and to detect genome‐wide associations for additive‐genetic and dominance effects for TL, tail circumference (TC), and tail abnormalities, and to annotate potential candidate genes.

## MATERIALS AND METHODS

### Study and mating design

**FIGURE 1:**
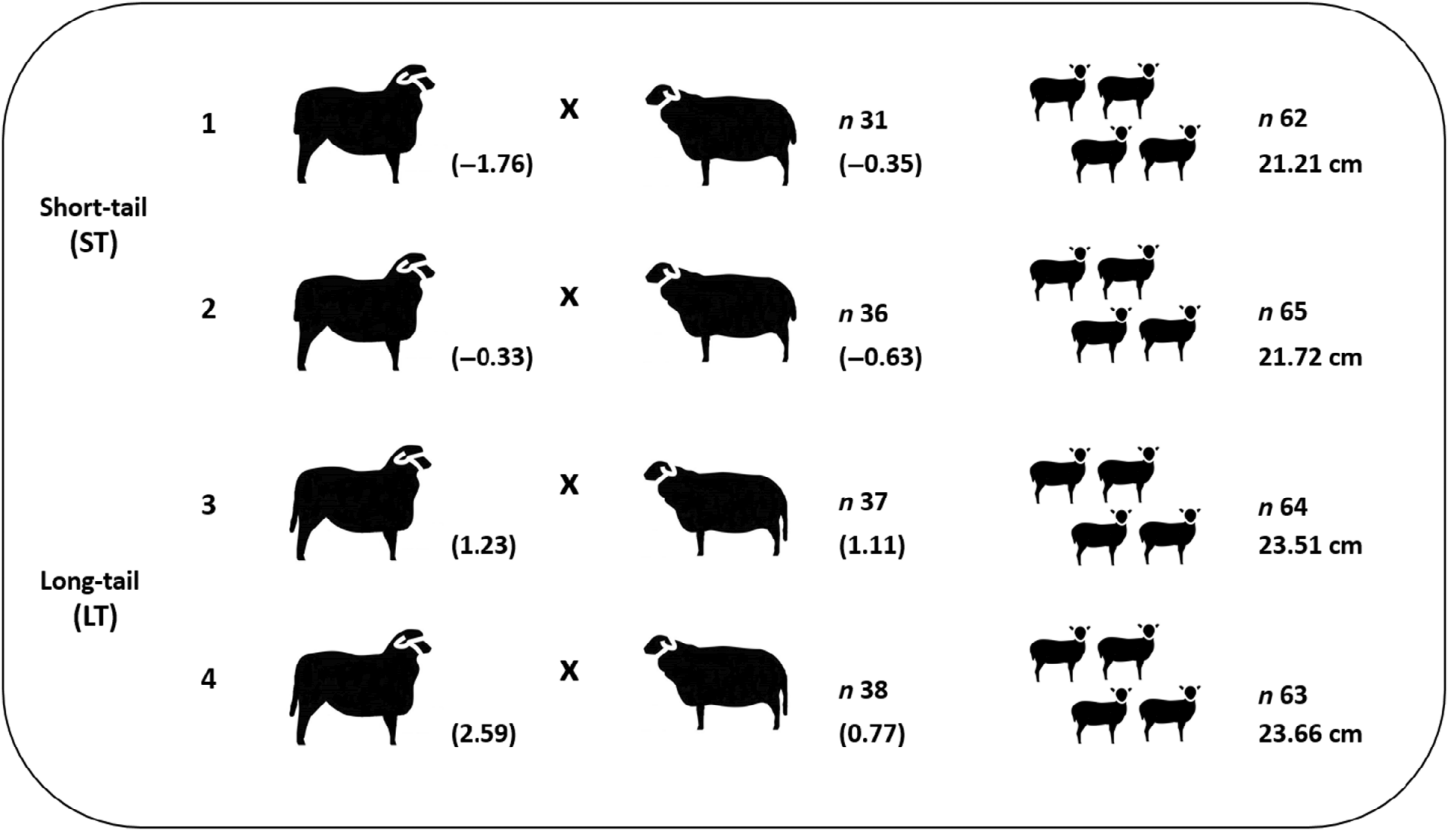
Selective mating experiment: rams (1–4) with estimated breeding values (EBV) for tail length (in parentheses), number (*n*) of mating ewes with average EBV for tail length (in parentheses), and number (*n*) of respective lambs and their average tail length within the four mating groups.

The selection and mating experiment aimed on creating a highly divergent experimental population of Merinoland sheep lambs for TL. Therefore, four rams with extreme estimated breeding values (EBV) for TL were mated with 142 ewes, which were also selected based on their TL EBV. Breeding value estimations considered the pedigree‐based relationship matrix in a single‐trait animal model, and included TL records from 2008 Merinoland lambs, measured over a period of 9 years (2013–2021) at the research station “Oberer Hardthof” of Justus‐Liebig University Gießen, Germany (see Oberpenning et al., 2023). Rams 1 and 2 were short‐tailed with low EBV for TL (−1.76 and −0.33, respectively), and rams 3 and 4 were long‐tailed with high EBV (1.23 and 2.59, respectively). Thus, mating within the four selection groups followed the ‘short × short’ (ST) or ‘long × long’ (LT) strategy (Figure 1). The average EBV of ST ewes mated with ram 1 was −0.35, the average EBV of ST ewes mated with ram 2 was −0.63, the average EBV of LT ewes mated with ram 3 was 1.11, and the average EBV of LT ewes mated with ram 4 was 0.77. The selection and mating experiment was conducted in August and September 2021 at the research station “Oberer Hardthof”. Ewes in heat were detected via intensified visual observations, and afterwards, mated with the selected ram applying the hand mating method. The matings generated 254 lambs, with almost identical lamb numbers in the four mating groups (62 and 65 lambs in the ST groups, and 64 and 63 lambs in the LT groups; see Figure 1). The mean TL measurements from the ST and the LT lamb groups differed significantly (*p* < 0.05).

### Trait recording

The 254 lambs (= offspring from the selection and mating experiment) were phenotyped at birth for tail measurements, body measurements, tail characteristics and tail abnormalities. The tail measurements included TL and TC. TL (in cm) was measured from the tail base to the tip of the tail, and TC (in cm) at the base of the tail. Body measurements included body length (BL, in cm) measured at birth using a self‐made construction with integrated measuring tapes, and BL comprising the distance (in cm) from the first thoracic vertebra to the base of the tail. Body weight (BW, in kg) at birth was recorded using a standard electronic scale.

Caudal spines were examined radiographically. Traits of interest for the tail characteristics/abnormalities included the number of vertebrae (nVERT), and X‐ray images for the vertebral malformations block vertebrae (BLCKV) and wedged vertebrae (WDGV), fractures (FRC), and axis deviations (AXISD) of the tail. X‐ray images were taken using a portable X‐ray device (Physia GAMMA Light AD 100|20) with a setting of 40 kV and 2.5 mAs. Axis deviation was defined as a manually irreparable deviation of the tail from the medians. A BLCKV was detected if the vertebral boundaries of adjacent vertebrae were indistinct or fused. Wedge‐shaped bones identified between two vertebrae were classified as WDGV. A binary data structure was defined for AXISD, BLCKV, WDGV, and FRC. For all radiographic traits AXISD, BLCKV, WDGV, and FRC, the score = 1 was assigned for abnormalities, and otherwise, all animals without a diagnosis, received the score = 0 for the respective trait. In addition to the lambs from the selection experiment, 38 herd contemporaries were phenotyped for the traits described above. However, from these lambs, only phenotype and pedigree data were available, but no WGS data. The descriptive statistics for all traits are given in Table 1 for animals from the selection experiment and the additional herd contemporaries.

**TABLE 1:** Descriptive statistics of tail measurements (tail length [TL], tail circumference [TC]) body measurements (body length [BL], body weight [BW]), and tail characteristics/abnormalities (number of vertebrae [nVERT], axis deviation [AXISD], block vertebrae [BLCKV], wedged vertebrae [WDGV], fracture [FRC]) for the lambs from the selection experiment (*N* = 254, group = SEL) and for herd contemporaries from further matings without genome sequence data (*N* = 38, group = CON).

Trait	Group	Mean	SD	Min	Max	CV
TL (cm)	SEL	22.54	2.29	17.0	28.2	0.10
	CON	23.03	1.94	18.9	24.4	0.08
TC (cm)	SEL	6.54	0.50	3.4	7.9	0.08
	CON	6.74	0.45	3.8	7.7	0.07
BL (cm)	SEL	32.73	2.37	27.2	39.4	0.07
	CON	33.04	2.18	28.6	35.5	0.07
BW (kg)	SEL	5.43	0.95	2.9	8.7	0.18
	CON	5.72	0.80	3.4	8.0	0.14
nVERT	SEL	20.35	1.63	15	24	0.08
	CON	20.56	1.42	18	22	0.07
AXISD	SEL	0.15	0.36	0	1	2.40
	CON	0.12	0.28	0	1	2.33
BLCKV	SEL	0.13	0.34	0	1	2.62
	CON	0.09	0.18	0	1	2.00
WDGV	SEL	0.08	0.28	0	1	3.50
	CON	0.04	0.16	0	1	4.00
FRC	SEL	0.27	0.44	0	1	1.63
	CON	0.20	0.31	0	1	1.55

### DNA extraction, whole genome sequencing, and variant calling

From the 254 selection experiment lambs, blood samples for ongoing WGS were collected within the first week of life. Trait recording and blood sampling were approved by the Regional Council of Giessen (V 54—19c 20 15 h 01 GI 18/14 No. G 44/2021). DNA was extracted from blood (400 μL) using the NucleoSpin®Blood Kit (Machery‐Nagel GmbH & Co. KG, Düren, Germany) according to the manufacturer protocol. DNA with an absorbance ratio at 260 and 280 nm of ~1.8, representing pure DNA, was standardised to 60 ng/μL using the Nanodrop 1000 spectrophotometer (Peqlab, Erlangen, Germany).

WGS was performed using the Illumina NovaSeq 6000 instrument (Illumina, San Diego, CA, USA), which generated 150‐bp paired‐end reads with a 15× coverage. The mean mapped read depth per sample was 15.83 with maximum and minimum mapped depths per sample of 19.73 and 12.52, respectively. All libraries for each sequenced animal were de‐multiplexed using the Illumina bcl2fastq v2.20 software. We calculated the Phred score using the fastqc software package, version 0.11.9 (Andrews, 2010). The median PHRED score was 37 throughout the read length, indicating very high sequencing quality.

Sequencing adapters were trimmed for all reads, and reads with final length <20 bases and all flanking primers and adapters, were discarded. Afterwards, the sequence reads were aligned to the *Ovis aries* ARS‐UI_Ramb_v2.0 genome assembly. The mapping, variant calling, and variant filtering were carried out following a workflow named OVarFlow, which was developed for variant discovery of single nucleotide polymorphisms (SNPs) and insertions and deletions in model and non‐model organisms (Bathke & Lühken, 2021). The variants with a call rate >0.95, a minor allele frequency >0.05, and a non‐significant deviation from Hardy–Weinberg equilibrium (*p* < 1.0E‐06), were kept for the ongoing analyses. Finally, 22 603 883 biallelic variants on autosomes were available from the sequenced sheep. We focussed on autosomes, because we were interested in sex‐independent effects, and the known genes associated with TL are not located on sex chromosomes. At least 95% of the variants were successfully genotyped (called) for each sheep. Quality control was performed using the plink 1.9 package (Purcell & Chang, 2023).

### Population stratification

Due to the selective sequencing approach, it is imperative to correct for the population structure of the extreme lamb subgroups in the ongoing genomic analyses. The genomic variant‐based relationship matrix **G** was constructed considering all genetic variants and using the algorithm by Yang et al. (2010). The relationship matrix **G** was used afterwards for principal component analysis. The software package gcta (Yang et al., 2010) generated the first 20 principal components. The first two principal components explained 8.03% and 6.35% of the genetic variation for TL, respectively, and were considered in the ongoing genetic‐statistical modelling approaches (see Models 1, 2a, 2b and 3).

### Estimation of variance components

Variant‐based heritabilities for the Gaussian distributed tail measurements (TL, TC) and body measurements (BL, BW), and for the count data for the tail characteristic nVERT, were estimated via linear single‐trait animal models with additive‐genetic or with additive‐genetic and dominance effects using the ‐‐reml function as implemented in the gcta software package (Yang et al., 2010). For the binary distributed tail abnormalities AXISD, BLCKV, WDGV, and FRC, the GREML approach in gcta was extended to a case–control design as specified in Lee et al. (2011). In this regard, we specified the phenotypic values as 1 or 0, and the prevalence for abnormalities with 15.28 for AXISD, 12.89% for BLCKV, 8.33% for WDGV, and for 27.73% for FRC. With these specifications, the case–control approach implies a transformation of heritabilities from the observed to underlying liability scale. The respective single‐trait Models 1 (without dominance) and 2a (additionally with dominance) were defined as follows:

(1) $$y=\mathrm{Xb}+Z_{g}g+e$$

(2a) $$y=\mathrm{Xb}+Z_{g}g+Z_{d}d+e$$

where **y** = vector of observations for TL, TC, BL, BW, nVERT, AXISD, BLCKV, WDGV, and FRC; **b** = vector of fixed effects including sex (male, female), birth type (class: single, twin, triplet), parity (class: 1–4), and the first two principal components from the genomic variant‐based relationship matrix; **g** = vector of additive‐genetic effects following *N* (0, **G** $\sigma_{g}^{2}$), where **G** = the genomic variant‐based relationship matrix constructed according to Yang et al. (2010), and $\sigma_{g}^{2}$ = the genomic variance; **d** = vector of dominance effects following *N* (0, **D** $\sigma_{d}^{2}$), where **D** = the genomic dominance relationship matrix constructed according to Zhu et al. (2015), and $\sigma_{d}^{2}$ = the dominance variance; **e** = vector of random residuals following *N* (0, **I** $\sigma_{e}^{2}$), where **I** = an identity matrix, and $\sigma_{e}^{2}$ = the residual variance; and **X**, **Z**_**g**_ and **Z**_**d**_ = incidence matrices for fixed effects, random additive‐genetic effects, and random dominance effects, respectively.

For all traits, EBV were estimated from Model 1. Afterwards, we calculated the correlations between EBVs from lambs with own phenotypic records for all traits. Estimated breeding values for traits with high heritability and based on own records tend to be very accurate, and therefore their correlations are a good approximation to the genetic correlation between traits.

Theoretically, EBV correlations are always an underestimation of genetic correlations with less extreme values, because EBV accuracies are smaller than 1 (Calo et al., 1973).

Because of the limited number of lambs with genome sequences for variance component estimations and failed convergence for the binary traits with Model 2a (as outlined in the results section), we run a Model 2b additionally considering the 38 lambs with phenotypic records, but without genome sequences. In this regard, for the total dataset including 294 lambs, we focussed in Model 2b on a pedigree‐based approach by constructing additive‐genetic relationships and dominance relationships considering the sheep pedigrees back to birth year 1995. All other effects and model specifications were the same as outlined for Model 2a. For the binary traits AXISD, BLCKV, WDGV, and FRC, threshold methodology was applied by defining a probit‐link function as implemented in the software package dmu (Madsen & Jensen, 2010).

### Genome wide associations for direct genetic and for dominance effects

Subsequently, we estimated additive‐genetic and dominance effects for each variant and all traits via GREML applications using gcta (Yang et al., 2010). Again, the GREML with the case–control specification was defined for binary tail abnormalities AXISD, BLCKV, WDGV, and FRC. Model 3 to estimate both effects of variant *i* simultaneously (according to Aliloo et al., 2015) was defined in matrix notation as follows:

(3) $$y=\mathrm{Xb}+x_{a}b_{a}+x_{d}b_{d}+Z_{g}g+e$$

where **y** = the vector of traits as stated above; **b** = vector of fixed effects as considered in Models 1 and 2 and again the first two principal components of **G** to account for population stratifications; $x_{a}$ = vector of centred genotypes for variant *i* calculated as 0 – 2*p*, 1 – 2*p*, and 2 – 2*p* (*p* = allele frequency for the *i*th variant) for variant *i* with original genotypes of 0, 1, and 2, respectively; $b_{a}$ = a regression coefficient representing the additive‐genetic effect for the *i*th variant; $x_{d}$ = a vector containing −2*p* ^2^, 2*pq*, and −2*q* ^2^ for variant *i* with original genotypes of 0, 1, and 2, respectively; $b_{d}$ = a regression coefficient for the dominance effect of the *i*th variant; **g** = vector of additive‐genetic effects following *N* (0, **G** $\sigma_{g}^{2}$); and **e** = vector of random residuals following *N* (0, **I** $\sigma_{e}^{2}$), where **I** = an identity matrix. The incidence matrices **X** and **Z**_**g**_ were the same as defined for Models 1 and 2a. The estimations of SNP effects for the additive‐genetic and dominance component was done as specified by Aliloo et al. (2015).

The test statistic for the additive‐genetic effect of variant *i* as estimated in consecutive runs for each variant was: $\chi_{a}^{2}=\frac{{\hat{b_{a}}}^{2}}{\mathrm{var}\left(\hat{b_{a}}\right)}$, with 1 degree of freedom (df). The test statistic for the dominance effect of variant *i* was: $\chi_{d}^{2}=\frac{{\hat{b_{d}}}^{2}}{\mathrm{var}\left(\hat{b_{d}}\right)}$, with 1 df. The inflation factor (𝜆) was calculated based on the test statistics mentioned above as: $\hat{\lambda }=\frac{\text{median}\left(\chi_{i}^{2}\right)}{\chi_{0.5,\mathrm{df}=1}^{2}}$, where $\chi_{i}^{2}$ = $\chi_{a}^{2}$ or $\chi_{d}^{2}$, and $\chi_{0.5,\;\mathrm{df}=1}^{2}$ = 0.4549 (the statistic for a probability of 0.5 from a chi‐squared distribution with 1 df). The *p*‐values of the additive‐genetic and dominance effects for each variant were determined by $\chi_{a}^{2}$ and $\chi_{d}^{2}$, respectively. Significantly associated variants were detected according to the Bonferroni correction calculated as *p*_Bonf_ = 0.05/(effective number of independent variants) = 0.05/477 627 = 1.05 × 10^−7^, implying −log_10_(*p*_Bonf_) = 6.98. The effective number of independent variants was calculated applying the plink 1.9 software package (Purcell & Chang, 2023). For the calculation of the effective number of independent variants, one sequence variant of a sequence variance pair in linkage disequilibrium with *r* ^2^ > 0.5 was excluded using the ‐‐indep‐pairwise option in plink (Purcell & Chang, 2023). In this regard, a window size of 5000 single variants was chosen, which was shifted in an interval of 500 single variants. Additionally, a less stringent correction was applied and defined according to Guo et al. (2017) as suggestive threshold with *p*_Sug_ = 1/(effective number of independent variants), resulting in −log_10_(*p*_Sug_) = 5.67.

### Annotation of potential candidate genes

Potential candidate genes were retrieved from the NCBI database, which based on the *O. aries* ARS‐UI_Ramb_v2.0 genome assembly. The candidate genes were assigned manually to the corresponding significant or candidate variants. In this regard, a gene was considered as a candidate gene if at least one single variant above the suggestive threshold was located in the respective gene or within a chromosomal segment comprising 1 kb up‐ and downstream of the respective candidate gene. The 1‐kb window was a very narrow chromosomal segment, but restricted the number of annotated potential candidate genes for the suggestive threshold.

In the final step, physiological functions of potential candidate genes were inferred based on information from the NCBI database (Brown et al., 2015).

## RESULTS

### Variance components and genetic parameters for tail measurements, tail abnormalities and body measurements

Genetic parameter estimates for all traits from the single‐trait Model 1 with additive‐genetic effects based on the genome sequence variants are given in Table 2. Table 3 includes the same variance components plus the estimates for the dominance variance from Model 2a (i.e., considering the variant based relationship matrix) for the linear tail and body measurements TL, TC, BL, and BW. The phenotypic and additive‐genetic variances for the binary traits AXISD, BLCKV, WDGV, and FRC from Model 1 were quite small and very close to zero. Test runs for these traits additionally considering dominance variation in Model 2a indicated convergence problems and extremely large standard errors of estimates due to the limited number of sheep with genome sequences. However, the increased sample size and applying the pedigree‐based approach via Model 2b implied reliable estimates for all variance components and all traits (also displayed in Table 3).

**TABLE 2:** Genetic parameters ($\sigma_{g}^{2}$, additive‐genetic variance; $\sigma_{d}^{2}$, dominance variance; $\sigma_{e}^{2}$, residual variance; $\sigma_{p}^{2}$, phenotypic variance; *h* ^2^, heritability ^a^) with respective standard errors in brackets from Model 1 (without dominance effects) for tail length (TL), tail circumference (TC), body length (BL), body weight (BW), number of vertebrae (nVERT), axis deviation (AXISD), block vertebrae (BLCKV), wedged vertebrae (WDGV), and fracture (FRC) considering the lambs from the selection experiment in Model 1.

Trait	Genetic parameters			
	$\sigma_{g}^{2}$	$\sigma_{e}^{2}$	$\sigma_{p}^{2}$	*h* ^2^
TL	4.19 (1.57)	0.76 (1.10)	4.95 (0.72)	0.85 (0.23)
TC	0.05 (0.06)	0.19 (0.06)	0.24 (0.03)	0.21 (0.24)
BL	3.53 (1.47)	0.63 (1.04)	4.15 (0.63)	0.85 (0.26)
BW	0.64 (0.26)	0.05 (0.18)	0.68 (0.11)	0.93 (0.27)
nVERT	0.78 (0.68)	1.87 (0.61)	2.64 (0.35)	0.29 (0.24)
AXISD	<0.00 (0.04)	0.13 (0.03)	0.13 (0.02)	<0.00 (0.26)
BLCKV	0.01 (0.01)	0.10 (0.02)	0.11 (0.01)	0.09 (0.13)
WDGV	<0.00 (0.02)	0.08 (0.02)	0.08 (0.01)	<0.00 (0.20)
FRC	<0.00 (0.02)	0.18 (0.03)	0.18 (0.02)	0.01 (0.11)

^a^ For the binary traits AXISD, BLCKV, WDGV, and FRC, a case–control approach as implemented in the software package gcta was applied, and heritabilities are transformed to the underlying liability scale.

**TABLE 3:** Variance components ($\sigma_{g}^{2}$, additive‐genetic variance; $\sigma_{d}^{2}$, dominance variance; $\sigma_{e}^{2}$, residual variance; $\sigma_{p}^{2}$, phenotypic variance) and heritabilities (*h* ^2^) with respective standard errors in brackets from Model 2a (with dominance effects and considering the genome sequences from the selection experiment sheep) for tail length (TL), tail circumference (TC), body length (BL), and body weight (BW) and from Model 2b (with dominance effects and considering the pedigree relationships from all sheep) for TL, TC, BL, BW, number of vertebrae (nVERT), axis deviation (AXISD), block vertebrae (BLCKV), wedged vertebrae (WDGV), and fracture (FRC).

Trait	Model	Genetic parameters				
		$\sigma_{g}^{2}$	$\sigma_{d}^{2}$	$\sigma_{e}^{2}$	$\sigma_{p}^{2}$	*h* ^2^
TL	2a	3.99	0.74	0.22	4.95	0.81 (0.27)
	2b	4.50	1.03	0.56	6.09	0.74 (0.19)
TC	2a	0.09	<0.00	0.26	0.35	0.25 (0.35)
	2b	0.16	0.03	0.64	0.83	0.19 (0.20)
BL	2a	3.37	0.62	0.16	4.16	0.81 (0.30)
	2b	4.22	0.70	0.44	5.36	0.79 (0.18)
BW	2a	0.55	<0.00	0.12	0.67	0.82 (0.37)
	2b	0.87	0.03	0.39	1.29	0.67 (0.17)
nVERT	2b	1.15	0.06	2.97	4.18	0.28 (0.16)
AXISD	2b	0.04	<0.00	1	1.04	0.04 (0.21)
BLCKV	2b	0.15	0.01	1	1.16	0.13 (0.20)
WDGV	2b	0.02	<0.00	1	1.02	0.02 (0.18)
FRC	2b	0.05	<0.00	1	1.05	0.05 (0.21)

For the linear traits, the additive‐genetic variances from the model with additive‐genetic effects (Model 1) and with additive‐genetic and dominance effects (Model 2a) differed only marginally. Additive‐genetic variances for the tail and body measurements in cm were 4.19 ± 1.57 and 3.99 ± 1.66 for TL, 0.05 ± 0.06 and 0.09 ± 0.13 for TC, 3.53 ± 1.47 and 3.37 ± 1.56 for BL, from Models 1 and 2a, respectively. The additive‐genetic variances for BW in kg were 0.64 ± 0.26 from Model 1 and 0.55 ± 0.29 from Model 2a. Accordingly, due to the pronounced genetic variation, direct heritabilities were moderate to large, i.e., 0.85 ± 0.23 and 0.81 ± 0.27 for TL, 0.85 ± 0.26 and 0.81 ± 0.30 for BL, and 0.93 ± 0.27 and 0.82 ± 0.37 for BW (results from Model 1). In analogy to the genetic variances, heritability estimates only differed marginally from the model without (Model 1) and with dominance (Model 2a). Moderate additive‐genetic variation was also found for nVERT with 0.78 ± 0.68, but genetic variances were close to zero in the range <0.001–0.01 for the binary tail abnormalities (AXISD, BLCKV, WDGV, FRC). The direct heritability for nVERT was 0.29 ± 0.24 and 0.09 ± 0.13 for BLCKV, but heritabilities were very small in a range from <0.001 to 0.01 for the tail abnormalities AXISD, WDGV, and FRC. The dominance variances from Model 2a were 0.74 ± 0.06 for TL, <0.00 ± 0.03 for TC, 0.62 ± 0.09 for BL, and <0.00 ± 0.06 for BW.

For the linear tail and body measurements, variance components and heritabilities indicated only minor differences from the genomic Model 2a and the pedigree‐based Model 2b. We observed generally larger variance components from Model 2b. However, the stronger increase of the residual compared to the additive‐genetic component explains the smaller heritabilities from the pedigree‐based approach. The dominance variances from Model 2b were very close to zero for all binary traits. The application of a threshold Model 2b with a probit‐link function implies a fixation of the residual variance (value 1) for all binary trait abnormalities.

### Breeding value correlations among tail measurements, tail abnormalities, and body measurements

Pearson correlation coefficients among EBV of all traits and considering the lambs with phenotypes are given in Table 4. Correlations were strong and positive between TL and TC (0.60), and between TL and the body measurements BL (0.54) and BW (0.50). The positive EBV correlations indicate longer and thicker tails for taller and heavier lambs. The largest EBV correlation was found between TL and nVERT with 0.73. Positive correlation coefficients were also found between nVERT with the remaining body measurements TC (0.23), BL (0.28), and BW (0.14). TL EBV were positively correlated with EBV for the tail abnormalities AXISD (0.30) and FRC (0.57), indicating fewer axis deviations and fractures when breeding short‐tailed sheep. In contrast, the correlation coefficients were negative (unfavourable) between TL and the vertebral malformations BLCKV (−0.35) and WDGV (−0.24). Regarding the number of vertebrae and tail abnormalities, correlation coefficients were positive between nVERT with AXISD (0.30) and FRC (0.46), but negative between nVERT with BLCKV (−0.21) and WDGV (−0.18). The AXISD EBV were negatively correlated with EBV for the vertebral malformations BLCKV (−0.27) and WDGV (−0.15), but positively correlated with the EBV for FRC (0.42). EBV between BLCKV and WDGV were closely correlated with 0.50. Both tail abnormalities BLCKV and WDGV were negatively correlated with FRC (−0.68 and −0.40, respectively).

**TABLE 4:** Pearson correlation coefficients among estimated breeding from Model 1 (without dominance effects) including tail measurements (tail length [TL], tail circumference [TC]), body measurements (body length [BL], body weight [BW]), and tail abnormalities (number of vertebrae [nVERT], axis deviation [AXISD], block vertebrae [BLCKV], wedged vertebrae [WDGV], fracture [FRC]).

	TC	BL	BW	nVERT	AXISD	BLCKV	WDGV	FRC
TL	0.60***	0.54***	0.50***	0.73***	0.30***	−0.35***	−0.24**	0.57***
TC		0.44***	0.53***	0.23**	0.18*	−0.30***	−0.14	0.45***
BL			0.66***	0.28**	0.14	−0.26**	−0.09	0.32***
BW				0.14	0.03	−0.12	0.03	0.25**
nVERT					0.30***	−0.21*	−0.18*	0.46***
AXISD						−0.27**	−0.15	0.42***
BLCKV							0.50***	−0.68***
WDGV								−0.40***

*p ≤ 0.05; **p ≤ 0.01; ***p ≤ 0.001 (test for significant deviation from a correlation coefficient of zero).

### Genome‐wide associations for direct genetic and dominance effects and respective gene annotations

The genome‐sequence data enabled the detection of a quite large number of significant variants according to the suggestive threshold, especially for the additive‐genetic component and the large heritability traits. Deeper interpretations with focus on the most important chromosomal segments and potential candidate genes are outlined in the discussion section.

**FIGURE 2:**
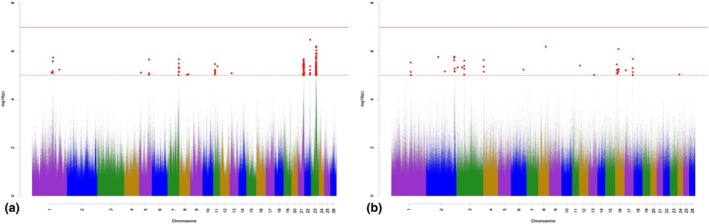
Manhattan plots for −log_10_ *p*‐values of variant effects for additive‐genetic effects (a) and for dominance effects (b) for tail length. Red solid line: genome‐wide significance threshold (*p*_Bonf_) according to Bonferroni; red dotted line: suggestive candidate threshold (*p*_Sug_).

The Manhattan plots for variant associations for TL are presented in Figure 2. With regard to the GWAS for additive‐genetic effects of TL (Figure 2a), we identified 81 significantly associated variants, which were annotated with 26 potential candidate genes. None of the variants exceeded *p*_Bonf_. The largest number of significantly associated variants was detected on OAR 7, 11, 21, and 23. Annotated potential candidate genes are involved in the skeletal development and in fat metabolism (Table S1). The GWAS for the dominance effects for TL (Figure 2b) detected 17 significantly associated variants, which were annotated with 12 potential candidate genes. The largest number of significantly associated variants according to *p*_Sug_ was detected on OAR 2, 3, 16 and 17. The annotated potential candidate genes are involved in the regulation of bone formation and growth (Table S2).

**FIGURE 3:**
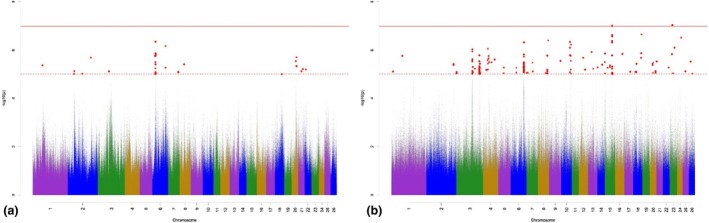
Manhattan plots for −log_10_ *p*‐values of variant effects for additive‐genetic effects (a) and for dominance effects (b) for tail circumference. Red solid line: genome‐wide significance threshold (*p*_Bonf_) according to Bonferroni; red dotted line: Suggestive candidate threshold (*p*_Sug_).

The Manhattan plots for variant associations for TC are presented in Figure 3. With regard to the GWAS for additive‐genetic effects of TC (Figure 3a), we identified 16 significantly associated variants, which were annotated with 11 potential candidate genes. None of the variants surpassed the Bonferroni significance threshold. The largest number of significantly associated variants was detected on OAR 6. Possibly important annotated potential candidate genes are part of skeletal development through regulation of osteoblast differentiation and fat metabolism (Table S1). The GWAS for the dominance effects for TC (Figure 3b) detected 56 significantly associated variants, which were annotated with 46 potential candidate genes. The largest number of significantly associated variants according to *p*_Sug_ was detected on OAR 3, 6, 10, and 15. The annotated potential candidate genes are involved in skeletal muscle growth and development (Table S2).

**FIGURE 4:**
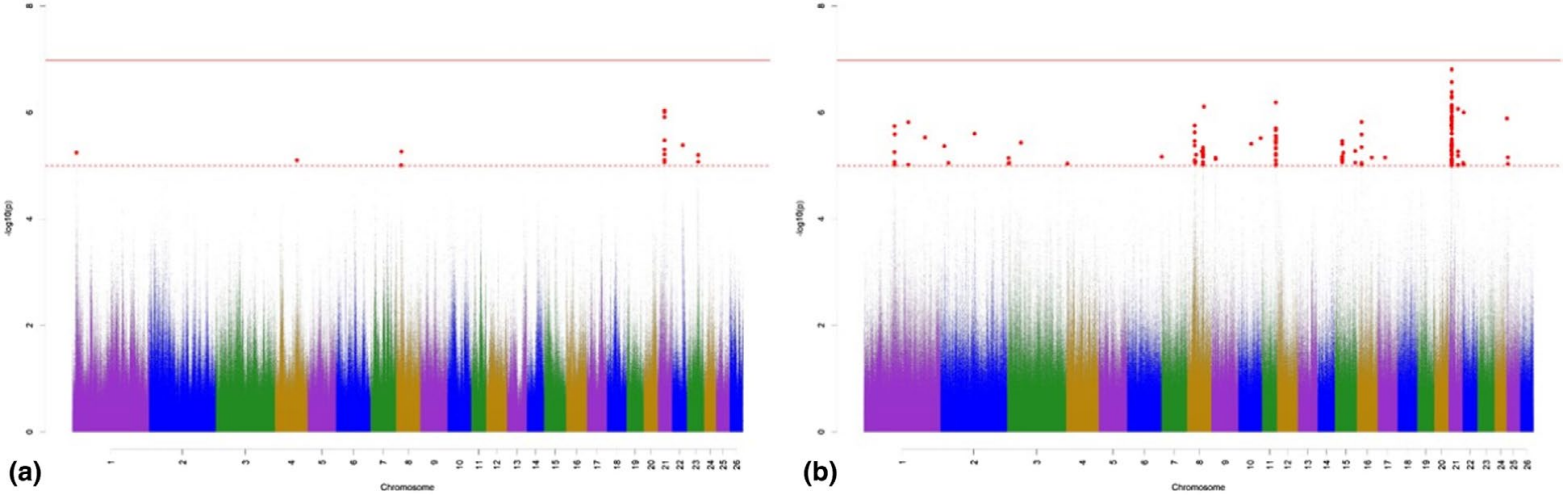
Manhattan plots for −log_10_ *p*‐values of variant effects for additive‐genetic effects (a) and for dominance effects (b) for the number of vertebrae. Red solid line: genome‐wide significance threshold (*p*_Bonf_) according to Bonferroni; red dotted line: suggestive candidate threshold (*p*_Sug_).

The Manhattan plots for variant associations for nVERT are presented in Figure 4. With regard to the GWAS for additive‐genetic effects (Figure 4a), we identified three significantly associated variants, which were annotated with three potential candidate genes. None of the variants surpassed the Bonferroni significance threshold. The three significant variants according to *p*_Sug_ are located on OAR 8 and 23. Possibly important annotated potential candidate genes are shown in Table S1. The GWAS for the dominance effects for nVERT (Figure 4b) detected 100 significantly associated variants, which were annotated with 18 potential candidate genes. The largest number of significantly associated variants according to *p*_Sug_ was detected on OAR 8, 11, and 21. The annotated potential candidate genes are part of skeletal development and growth (Table S2).

**FIGURE 5:**
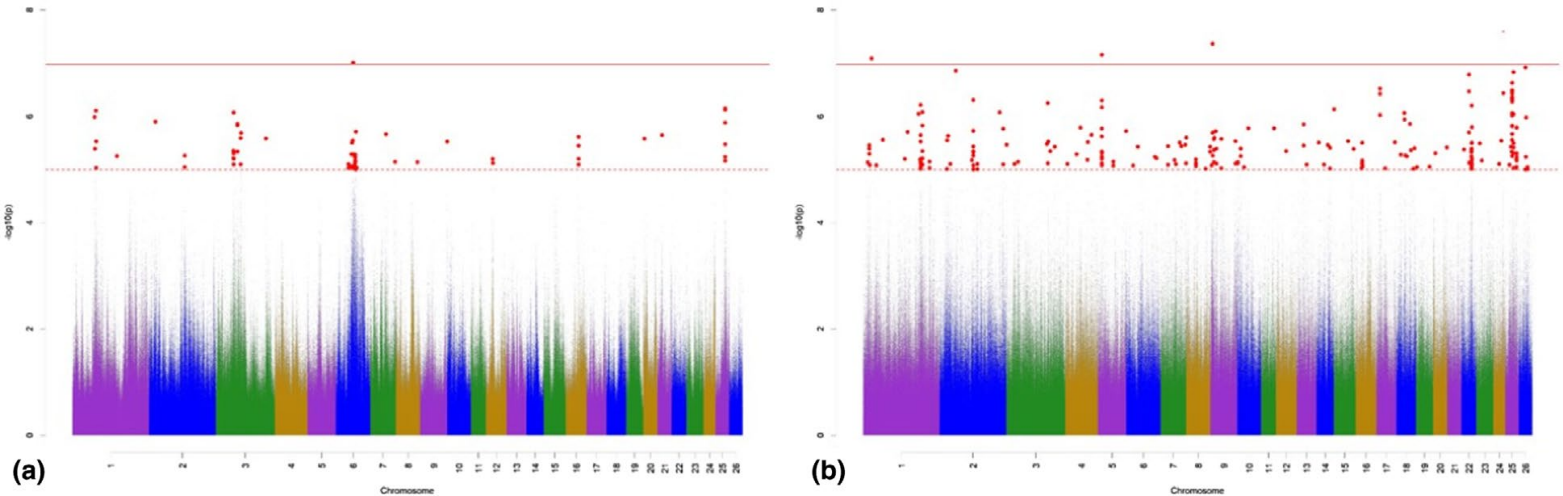
Manhattan plots for −log_10_ *p*‐values of variant effects for additive‐genetic effects (a) and for dominance effects (b) for axis deviation. Red solid line: genome‐wide significance threshold (*p*_Bonf_) according to Bonferroni; red dotted line: suggestive candidate threshold (*p*_Sug_).

The Manhattan plots for variant associations for AXISD are presented in Figure 5. With regard to the GWAS for additive‐genetic effects of AXISD (Figure 5a), we identified 21 significantly associated variants, which were annotated with 15 potential candidate genes. One of the variants on OAR 6 surpassed the Bonferroni significance threshold. The largest number of significantly associated variants was detected on OAR 6. Possibly important annotated potential candidate genes regulate growth traits (Table S1). The GWAS for the dominance effects for AXISD (Figure 5b) detected 134 significantly associated variants, which were annotated with 65 potential candidate genes. The largest number of significantly associated variants according to *p*_Sug_ was detected on OAR 5, 22, and 25. The annotated potential candidate genes are responsible for mesenchymal cell differentiations, and contribute to bone and cartilage development (Table S2).

**FIGURE 6:**
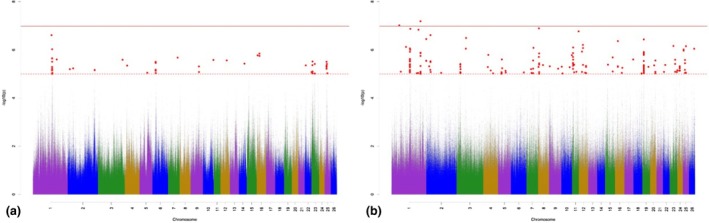
Manhattan plots for −log_10_ *p*‐values of variant effects for additive‐genetic effects (a) and for dominance effects (b) for block vertebrae. Red solid line: genome‐wide significance threshold (*p*_Bonf_) according to Bonferroni; red dotted line: suggestive candidate threshold (*p*_Sug_).

The Manhattan plots for variant associations for BLCKV are presented in Figure 6. With regard to the GWAS for additive‐genetic effects of BLCKV (Figure 6a), we identified 20 significantly associated variants, which were annotated with 17 potential candidate genes. None of the variants was significant according to *p*_Bonf_. The largest number of significantly associated variants was detected on OAR 1, 23, and 25. Possibly important annotated potential candidate genes are involved in bone formation through osteoblast development (Table S1). The GWAS for the dominance effects for BLCKV (Figure 6b) detected 65 significantly associated variants, which were annotated with 38 potential candidate genes. Two of the variants located on OAR 1 were significant according to *p*_Bonf_. The largest number of significantly associated variants according to *p*_Sug_ was detected on OAR 1, 3, and 19. The annotated potential candidate genes are involved in bone formation and chondrogenic differentiation (Table S2).

**FIGURE 7:**
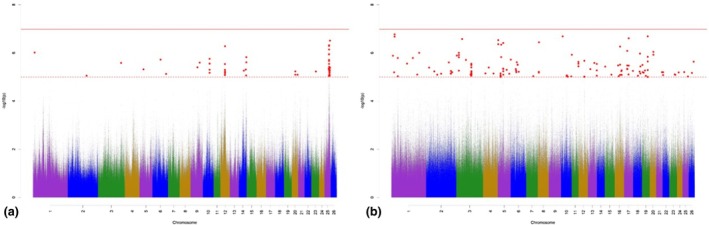
Manhattan plots for −log_10_ *p*‐values of variant effects for additive‐genetic effects of (a) and for dominance effects (b) for wedged vertebrae. Red solid line: genome‐wide significance threshold (*p*_Bonf_) according to Bonferroni; red dotted line: suggestive candidate threshold (*p*_Sug_).

The Manhattan plots for variant associations for WDGV are presented in Figure 7. With regard to the GWAS for additive‐genetic effects of WDGV (Figure 7a), we identified 22 significantly associated variants, which were annotated with 11 potential candidate genes. None of the variants was significant according to *p*_Bonf_. The largest number of significantly associated variants was detected on OAR 25. Annotated potential candidate genes are involved in skeletal development and development of skeletal anomalies (Table S1). The GWAS for the dominance effects for WDGV (Figure 7b) detected 63 significantly associated variants, which were annotated with 37 potential candidate genes. One of the variants on OAR 23 was significant according to *p*_Bonf_. The largest number of significantly associated variants according to *p*_Sug_ was detected on OAR 1, 5, 12 and 19. The annotated potential candidate genes regulate mechanisms of osteogenesis (Table S2).

**FIGURE 8:**
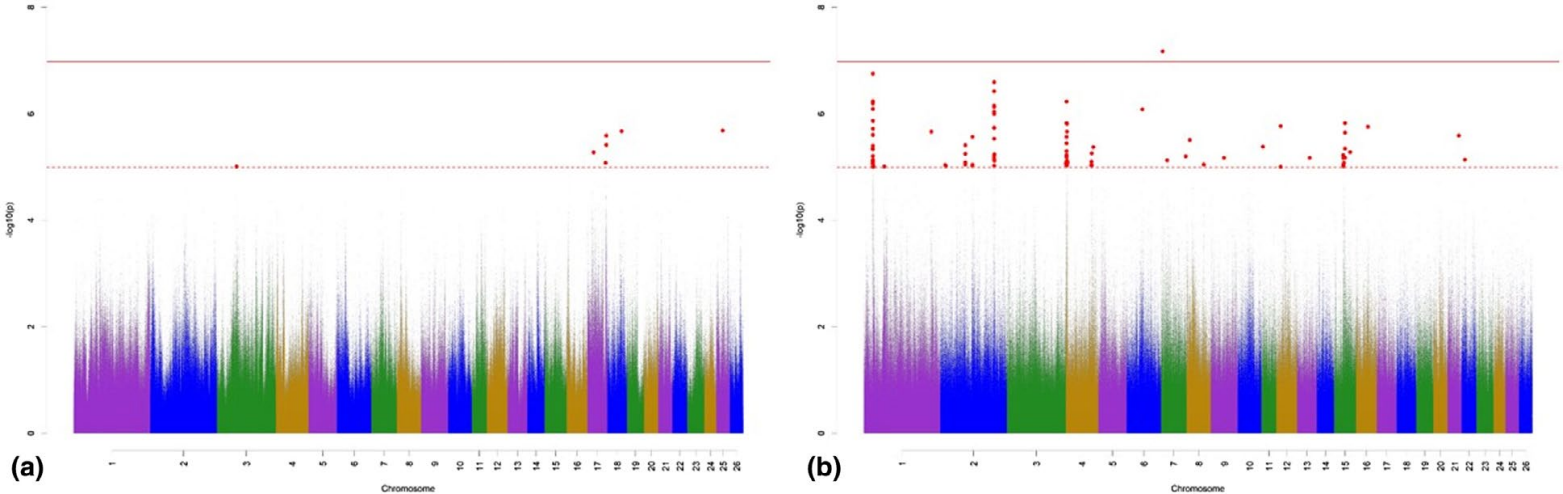
Manhattan plots for −log_10_ *p*‐values of variant effects for additive‐genetic effects (a) and for dominance effects (b) for fractures. Red solid line: genome‐wide significance threshold (*p*_Bonf_) according to Bonferroni; red dotted line: suggestive candidate threshold (*p*_Sug_).

The Manhattan plots for variant associations for FRC are presented in Figure 8. With regard to the GWAS for additive‐genetic effects of FRC (Figure 8a), we identified three significantly associated variants, which were annotated with two potential candidate genes. None of the variants surpassed the Bonferroni significance threshold. Annotated potential candidate genes are shown in Table S1. The GWAS for the dominance effects for FRC (Figure 8b) detected 65 significantly associated variants, which were annotated with 23 potential candidate genes. One variant on OAR 7 was significant according to *p*_Bonf_. The largest number of significantly associated variants according to *p*_Sug_ was detected on OAR 1, 2, 4, and 15. The annotated potential candidate genes are involved in bone metabolism and osteoblast development (Table S2).

The Manhattan plots for variant associations for BL are presented in Figure S1. With regard to the GWAS for additive‐genetic effects of BL (Figure S1a), we identified 24 significantly associated variants, which were annotated with five potential candidate genes. None of the variants surpassed the Bonferroni significance threshold. The largest number of significantly associated variants was detected on OAR 5. Possibly important annotated potential candidate genes are involved in spine maturation and fat metabolism (Table S1). The GWAS for the dominance effects for BL (Figure S1b) detected 13 significantly associated variants, which were annotated with 12 potential candidate genes. The largest number of significantly associated variants according to *p*_Sug_ was detected on OAR 16 and 20. The annotated potential candidate genes are part of muscle development and growth (Table S2).

The Manhattan plots for variant associations for BW are presented in Figure S2. With regard to the GWAS for additive‐genetic effects of BW (Figure S2a), we identified 13 significantly associated variants, which were annotated with seven potential candidate genes. None of the variants surpassed the Bonferroni significance threshold. The largest number of significantly associated variants was detected on OAR 24. Possibly important annotated potential candidate genes are involved in skeletal muscle development and growth (Table S1). The GWAS for the dominance effects for BW (Figure S2b) detected 10 significantly associated variants, which were annotated with six potential candidate genes. The largest number of significantly associated variants according to *p*_Sug_ was detected on OAR 16. The annotated potential candidate genes are involved in the regulation of bone formation and skeletal muscle development and growth (Table S2).

## DISCUSSION

### Variance components and genetic parameters for tail measurements, tail abnormalities, and body measurements

The large direct variant‐based heritabilities for TL with 0.85 and 0.81 from the genomic models with and without dominance, respectively, are in agreement with the estimate based on pedigree data (0.74) and reflect previous pedigree‐based estimates in other sheep breeds, e.g., a heritability of 0.82 in crosses of short‐tailed Finnish landraces with long‐tailed Cheviot sheep (Scobie & O'Connell, 2002). Lagler et al. (2022) reported an even higher heritability estimate (0.99) in specifically selected purebred Merino lambs. In their study, selection of animals based on extreme phenotypes for TL in only two farms. Thus, the possibly upwards biased heritability estimate could be due to similar phenotypes among closely related individuals (Zuk et al., 2012). Smaller heritabilities than in specific crosses or sub‐populations, but still in a moderate to large range from 0.30 to 0.60, were estimated in random samples of Merino populations (Greeff et al., 2015; Oberpenning et al., 2023).

In analogy to TL, the heritabilities for the linear body measurements BL and BW and considering the variant‐based relationship matrices were larger than 0.80, indicating the pronounced genetic background for accurately measured morphological traits. Similar findings were made by Vanvanhossou et al. (2020) when basing genetic parameter estimations of local indigenous cattle on body measurements in cm, or on weights recorded in kg. Nevertheless, the BL and BW heritabilities exceeded the estimates from other genetic sheep studies (Oberpenning et al., 2023; Oliveira et al., 2021). An explanation might be the selective sequencing approach as indicated by Zuk et al. (2012). However, in the genomic Models 1 and 2a, we corrected for possible population stratification. Also, the pedigree‐based modelling approach (Model 2b) supported the quite large heritabilities for body and tail measurements. Furthermore, the variant‐based genetic parameter estimations considering very dense WGS data might explain the larger heritabilities. Approximately one‐third to two‐third of the heritability are captured by common SNPs. The ‘missing’ heritability is explained through the large contribution of rare variants in low linkage disequilibrium, which can be considered in WGS approaches (Wainschtein et al., 2022).

To our knowledge, this is a first study estimating heritabilities for the number of caudal vertebrae in mammals. Our estimate for nVERT was moderate with 0.29. Donaldson (2016) reported heritabilities for the number of vertebrae in the cervical, thoracal, and lumbar spine of sheep in a broad range from 0.08 to 0.99, strongly depending on the section of the vertebral column. In pigs, estimated heritabilities for the number of vertebrae (cervical, thoracic, lumbar, and sacral) ranged from 0.35 to 0.62 (Borchers et al., 2004; Smith et al., 1962). Hence, the nVERT estimate as well as the heritability estimates for the number of vertebrae in other species and sections of the vertebral column indicate moderate to large heritabilities for ‘count data’ of body characteristics.

As expected, the heritabilities for the binary tail abnormalities were quite small and close to zero, reflecting the generally small genetic variations for diseases or disease indicators (Hulst et al., 2021). The importance of environmental factors in this regard addresses the intrauterine environment as well as traumatic impact. In such context, the number of lambs growing in the uterus of the ewe might be associated with abnormalities in morphological traits. Corsello and Piro (2010) indicated relationships between twin gestations with increased risks of congenital malformations in humans. Also, energy deficiency due to pregnancies with two or three foetuses, causing so‐called ‘gravidic toxaemia’ in sheep, might induce oxidative stress and cellular dysfunction (Lebovitz, 1995). Gutteridge (1995) observed morphological and functional cell damage due to physiological and metabolic alterations during late pregnancy. In humans, *diabetes mellitus* during pregnancy was associated with vertebral malformations (Giampietro et al., 2013). Additionally, foetal hypoxia may cause congenital vertebral malformations, because oxygen plays a crucial role as a metabolic regulator during embryogenesis (Li et al., 2015). Several studies have shown that hypoxia during pregnancy can cause spine deformities and congenital scoliosis in mice and humans (Hou et al., 2018; Loder et al., 2000). Low oxygen tension directly affects the vertebral cartilage primordium, resulting in congenital deformities in the cervical, thoracic, and lumbar regions of the spine in the mouse model (Loder et al., 2000). Environmental traumatic effects on vertebral malformations have been described by Hümmelchen et al. (2023) in sheep, and by Sinmez et al. (2017) in dogs. Furthermore, the heritability of binary traits strongly depends on the prevalence, with generally highest estimates for an intermediate prevalence (Hulst et al., 2021). In our study, prevalences were in a small to moderate range, with 15% for AXISD, 13% for BLCKV, 8% for WDGV, and 27% for FRC.

Dominance effects on TC and BW with <0% were negligible, but moderate dominance contributed to the phenotypic variation of TL (14.95%) and BL (14.90%). The estimates from the present study are in agreement with dominance effects in purebred and crossbred sheep for morphological traits, which ranged from 0% to 19.2% (Moghaddar & van der Werf, 2017). The percentage of dominance at the total phenotypic variation for different wool production traits in Merino sheep ranged from 2% to 35% (Konstantinov et al., 2002). In the pedigree‐based approach, the dominance variances for the binary tail abnormalities were also very small and close to zero. Zhu et al. (2020) suggested the application of models without dominance for traits with generally small additive‐genetic variation. In their study (Zhu et al., 2020), the model that additionally included dominant genetic effects did not improve the accuracies of genomic predictions for blood traits in Alpine Merino sheep.

### Breeding value correlations among tail measurements, tail abnormalities, and body measurements

It is imperative that breeding on short tails does not impair other sheep breeding goal traits, especially BW, and does not imply an associated risk of malformations, diseases, or abnormalities. In this regard, we estimated breeding values from the single trait Model 1, and correlated the EBV considering the lambs with phenotypes. The correlation coefficient between the EBV for TL with the EBV for nVERT was 0.73. The respective phenotypic correlation was 0.63, indicating increased TL with increasing nVERT. Genetic and phenotypic associations among both traits were similar in previous studies (Han et al., 2019; Zhi et al., 2018). In the present study, the EBV correlations were significantly different from zero and quite strong between TL and FRC (0.46), and between nVERT and FRC (0.57). Respective phenotypic correlations (Hümmelchen et al., 2023) were also positive, but substantially smaller with 0.17 and 0.16. Fractures are typically caused by trauma and occur more frequently in animals with longer tails and in tails with a larger number of vertebrae, as observed in another study assessing tail injuries in undocked animals (Hannemann et al., 2017). The negative EBV correlations between TL and BLCKV (−0.35) and between TL and WDGV (−0.24) indicate that breeding on shorter tails implies an increased prevalence for BLCKV and WDGV. Phenotypically, the respective correlations were close to zero (−0.04 and 0.03). The EBV correlations from the present study were confirmed in other genetic studies conducted in other species or populations. Schlensker and Distl (2016) estimated a negative genetic correlation of −0.39 between TL and the occurrence of hemivertebrae (WDGV) in French Bulldogs. Zhi et al. (2018) reported an increased incidence of malformed vertebrae in the tails of the short‐tailed Hulunbuir sheep population.

The positive EBV correlations between TL with AXISD and between nVERT with AXISD (both 0.30) slightly exceeded the respective phenotypic correlations. The EBV for AXISD were negatively correlated with the EBV for the vertebral malformations BLCKV and WDGV (−0.27 and −0.15, respectively), but positively correlated with FRC (0.42). Phenotypic correlations between AXISD with BLCKV, WDGV, and FRC were throughout positive (Hümmelchen et al., 2023), indicating an increased incidence of vertebral malformations for sheep with tail axis deviations. Schawalder et al. (2010) identified relationships between malformations of the vertebrae with kinks or AXISD in dog tails. Xu et al. (2016) investigated kinks in the tails of Asian domestic cats and the Japanese Bobtail. They observed a combined effect of various caudal vertebral deformities, including a reduced count for nVERT and the occurrence of WDGV and BLCKV in cats with kinked tails. Furthermore, AXISD of the tails were associated with separations in the intervertebral gap and vertebral FRC (Hümmelchen et al., 2023). Detailed investigations of anomalies of the tail vertebral column in dogs indicated that BLCKV and WDGV are anchored in a very early phase of embryonic development (Schawalder et al., 2010).

The EBV correlations between TL and the body measurements BL and BW were quite high with 0.54 and 0.50, respectively. Oberpenning et al. (2023) and Eck et al. (2019) presented very similar phenotypic correlations between TL and weights in randomly selected samples of Merinoland sheep. The positive EBV and positive phenotypic correlations suggest longer bodies and higher BW at birth of long‐tailed lambs. From a breeding perspective, breeding on shorter tails therefore has unfavourable effects on the development of body weight traits, implying an unfavourable effect on economy of sheep production.

### Genome‐wide associations for direct genetic and dominance effects and annotated potential candidate genes for tail traits

Overall, a quite large number of annotated potential candidate genes, for additive‐genetic as well as for dominance effects, could be related with functions of these genes in morphological trait developments, bone structures, or vertebrae characteristics in sheep as well as in other species.

With regard to the additive‐genetic effects for TL, significantly associated variants are located on OAR 13 in the intron of *CAMK1D*, which is a marker gene for osteoblasts (Wasserman et al., 2013). *CAMK1D* was associated with vertebral body development in Ujumqin sheep, and with the number of vertebrae in the thoracic and lumbar spine (Li et al., 2018). In addition, we detected variants in the intron of *PPP6R3* on OAR 21, which affected the development of the skeleton and cartilage in horses, and bone mineral density in humans (Pan et al., 2022). On OAR 23, we found variants in the intron of *PIEZO2*, which influenced bone development and osteoblast differentiation in mice (Zhou et al., 2020). *PIEZO2* was involved in the limb development of goats and in the aetiology of digital arthrogryposis (Delle Vedove et al., 2016). Homozygous individuals for mutations inactivating *PIEZO2* suffered from arthrogryposis and scoliosis, whereas carriers of gain‐of‐function mutations were susceptible to multiple congenital contractures of limbs and variable absence of cruciate knee ligaments (Coste et al., 2013). Detected variants on OAR 23 in introns of the genes *LAMA1*, *PTPRM*, and *SPIRE1* were associated with growth traits and body conformation traits such as body weight in sheep and goats (Abousoliman et al., 2021). We detected variants in the intron of *INSR* on OAR 5, as well as in the exons of *ACACA* on OAR 11, which were related with body weight, but also with fat metabolism and fat deposition in sheep and goats (Bakhtiarizadeh & Alamouti, 2020; Wang et al., 2022). With regard to TL dominance effects, we identified variants in the intron of *ANKRD26* on OAR 12. Polymorphisms of *ANKRD26* were linked to morphometric traits such as pin width in the dromedary (Sani et al., 2023), and gigantism in mice (Bera et al., 2008).

With regard to additive‐genetic effects for TC, we identified variants in the intron of *EXOC6B* on OAR3. Functions of *EXOC6B* in sheep addressed the development of body conformation traits, especially lamb metacarpal length (James et al., 2022). With regard to TC dominance effects, significant variants located on OAR 4 in the intron of *CREB5* regulated fat tail development in sheep (Yuan et al., 2016). Variants on OAR 8 were located in the intron of *PRKN* (aka *PARK2*), which influenced TL in sheep (Tao et al., 2021).

With regard to additive‐genetic effects for nVERT, we detected variants in introns of the genes *MAN1A1* and *MCM9* on OAR 8. Both genes influenced body weight and body conformation traits in different sheep breeds (Gholizadeh et al., 2015; Tao et al., 2020). Regarding dominance effects on nVERT, we annotated two very interesting candidate genes, *MAP3K5* (located on OAR 8) and *TMEM135* (located on OAR 21). In previous studies, *MAP3K5* was associated with morphological traits in sheep, including body height, BL, chest circumference, and cannon circumference (Kominakis et al., 2017; Yang et al., 2023). *TMEM135* contributed to bone growth and development in chicken, trough the regulation of ossification (Mohammadi et al., 2022).

With regard to additive‐genetic effects on AXISD, significant variants were located on OAR 2 in the introns of the gene *RAPGEF4*, and on OAR 8 in the intron of the gene *MAP3K4* gene. Both genes were suggested as possible candidates for fat‐tail type in sheep (Yuan et al., 2016). Regarding AXISD dominance effects, we detected significant variants in the intron of *ZNF121* on OAR 5. *ZNF121* influenced the development of intervertebral discs, and is responsible for intervertebral disc degeneration (Yuan et al., 2020). In addition, significant variants located in the introns of the genes *PCOLCE* on OAR 1, *PPM1H* on OAR 3, and *CADPS2* on OAR 4, were identified. *PCOLCE* was associated with bone formation and cartilage development in pigs (Li et al., 2021). *PPM1H* influenced the differentiation of mesenchymal cells into bone and cartilage tissue (Shen et al., 2014). *CADPS2* was associated with the occurrence of chondrodysplasia in Texel sheep (Zhao et al., 2012). Chondrodysplasia in Texel sheep is a recessively inherited disorder characterised by dwarfism and angular deformities of the forelimbs. Interestingly, we annotated *NRG3* on OAR 25, which was also associated with angular limb deformities in sheep. Respective gene effects addressed the regulation of transcription factors with known functions in bone growth (Becker et al., 2023). Further significant variants were identified in the intron of the gene *EML2* on OAR 14. The gene *EML2* was associated with bone formations during embryonic development, and with the number of lumbar vertebrae in donkeys (Sun et al., 2023).

With regard to additive‐genetic effects on BLCKV, significant variants were detected in the introns of the genes *ATP9B* and *KLHL14* on OAR 23, and in the intron of the gene *ZNF25* on OAR 25. These genes are involved in mechanism of bone metabolism or bone differentiation. Specifically, *ZNF25* was suggested as a candidate gene for osteoblast differentiation in humans (Twine et al., 2016), *KLHL14* as a candidate gene for bone strength in chicken (Yue et al., 2022), and *ATP9B* as a candidate gene regulating the progression of osteoarthritis (Zhou et al., 2018). Regarding BLCKV dominance effects, we detected significant variants located in the genes *BCKDHB* and *TGFB2*. Both genes contributed to malformations of bony structures, bone formation and bone degradation. The gene *BCKDHB* on OAR 8 was discovered in the context of angular limb deformity in Rambouillet rams (Becker et al., 2021). The *TGFB2* gene on OAR 12 affected the development of vertebrae and bone length as studied in knockout mice (Baffi et al., 2004).

With regard to additive‐genetic effects on WDGV, we detected significant variants in the introns of the genes *ZMIZ1* and *NRG3* on OAR 25, both influencing malformations of bony structures. *ZMIZ1* was associated with distal skeletal abnormalities and axial spondyloarthritis in humans (He et al., 2023), and *NRG3* with angular limb deformities in sheep (Becker et al., 2023). Regarding WDGV dominance effects, we annotated *FBN2* on OAR 5 as a potential candidate gene. Mutations in *FBN2* induced abnormal bone phenotypes, such as reduced bone formation in knockout mice, as well as skeletal abnormalities in humans (Chen et al., 2022). Furthermore, *FBN2* was associated with bone density (Pei et al., 2019).

With regard to GWAS for FRC, only significant variants for dominance effects were annotated with potential candidate genes. The significant variants were located on OAR 1 in the exon of the gene *CYP2J2*, on OAR 2 in the introns of the genes *FTCDNL1*, *SPATS2L*, and *ZNF804A*, and on OAR 15 in the intron of the gene *SOX6*. The gene *CYP2J2* is involved in the bio‐activation of vitamin D, which in turn affects the normal development of bones (Aiba et al., 2006). *FTCDNL1* played an important role in bone metabolism and in the development of osteoporosis in humans (Lu et al., 2015). Osteoporosis contributed to impaired bone density, and in causality increasing the risk of bone fractures. *SPATS2L* was associated with femoral head separation in chickens (Goldoni et al., 2022), and *ZNF804A* with fractures in racehorses (Blott et al., 2014). *SOX6* influenced bone mineral density, osteoblast development, chondrogenesis, and cartilage formation in humans (Yang et al., 2011), and activated chondrogenesis in murine fracture healing (Uusitalo et al., 2001).

Overall, the WGS GWAS combined with a selection experiment to create extreme phenotypes presented a quite large number of chromosomal regions harbouring potential candidate genes with known functions on morphological and skeletal traits in other species. However, with regard to TL, we did not find significant signals in relation to the previously reported TL‐candidate genes *HOXB13* and *TBXT* (Han et al., 2019; Li et al., 2022). The already described significant association of the nonsynonymous c.334G>T‐SNP of TBXT in different sheep breeds (Han et al., 2019; Li et al., 2022; Zhi et al., 2018) was not confirmed in the present study, probably due to breed and study concept differences. In the present study, we performed a unique selection and mating experiment for German Merinoland sheep. Accordingly, in Merinoland sheep, the study by Lagler et al. (2022) addressed non‐significant effects of *TBXT* mutations in Merinoland sheep. In contrast, for the gene *HOXB13*, Lagler et al. (2022) detected a causal insertion. However, this insertion is not considered in the ovine reference genome sequence, probably explaining the non‐significant associations in the present study. Consequently, the insertion in the promoter of *HOXB13* should be typed in a following complementary study using, e.g., polymerase chain reaction‐based methods. Such focus research is also imperative for all other suggested potential candidate genes.

## CONCLUSION

The quite large additive‐genetic variance and direct heritability for TL based on the variant‐based relationship matrix indicate the great potential to breed sheep with short tails within a few generations. However, strong breeding on short tails might be associated with unfavourable effects, i.e., a higher prevalence of some vertebral abnormalities as outlined via EBV correlations. In contrast, breeding on short tail implies a prevalence decline of fractures. Furthermore, dominance plays a role in the genetic architecture of TL in Merinoland sheep, but effects are negligible compared to the additive‐genetic component. In this regard, dominance explained 14.95% of the total phenotypic variation for TL. Genome‐wide associations based on WGS data revealed 726 significantly associated variants for both additive‐genetic and dominance effects. In ongoing gene annotations, 38 potential candidate genes were suggested for TL, and in total 354 potential candidate genes for all traits together. The potential candidate genes have known functions in the context of morphological trait developments and skeletal growth, and regulate the development of bone structures or of vertebrae characteristics. Hence, the created selection experiment based on extreme EBV groups for TL contributed to deeper insights into genomic and physiological mechanisms of TL, and associated tail characteristics.

## CONFLICT OF INTEREST STATEMENT

The authors declare that they have no conflict of interest.

## Supporting information


Figures S1–S2.

Table S1.

Table S2.

## Data Availability

Data supporting this study are openly available from the OSF repository at https://doi.org/10.17605/OSF.IO/BJTQZ, and GWAS results are openly available from the figshare repository at https://doi.org/10.6084/m9.figshare.27897057.v1
