# Supplementary material for: Genetic parameters, genome‐wide associations and potential candidate genes for additive and dominance effects of tail traits in Merinoland sheep based on whole‐genome sequence data in a selection experiment
Source: Anim Genet. 2025 Sep 18;56(5):e70041. doi: 10.1111/age.70041 (PMC12445261; doi:10.1111/age.70041)

**Supplementary figure 1.** Manhattan plots for –log(10) P-values of variant effects for additive genetic effects (**a**) and for dominance effects (**b**) for body length (BL). Red solid line: Genome-wide significance threshold (*P_Bonf_*) according to Bonferroni; Red dotted line: Suggestive candidate threshold (*P*_Sug_).
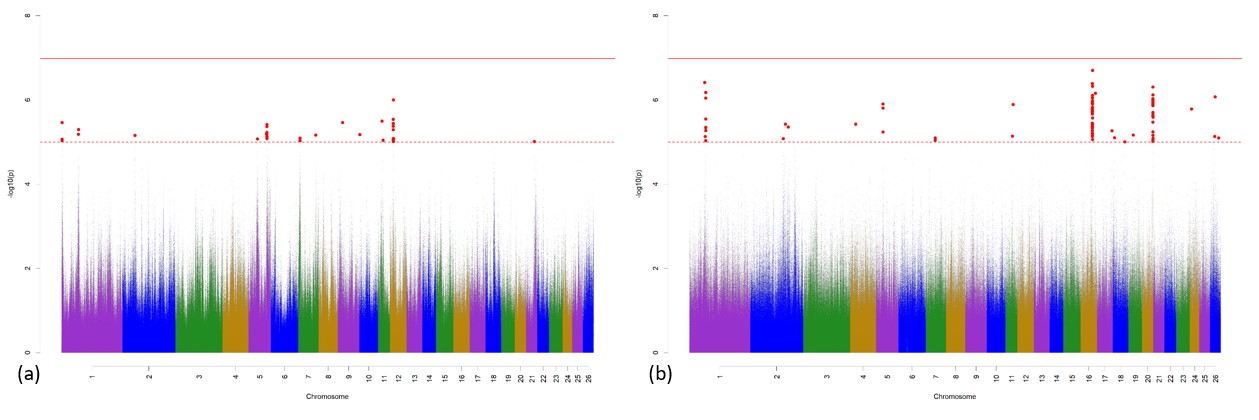


**Supplementary figure 2.** Manhattan plots for –log(10) P-values of variant effects for additive-genetic effects (**a**) and for dominance effects (**b**) for body weight (BW). Red solid line: Genome-wide significance threshold (*P_Bonf_*) according to Bonferroni; Red dotted line: Suggestive candidate threshold (*P*_Sug_).


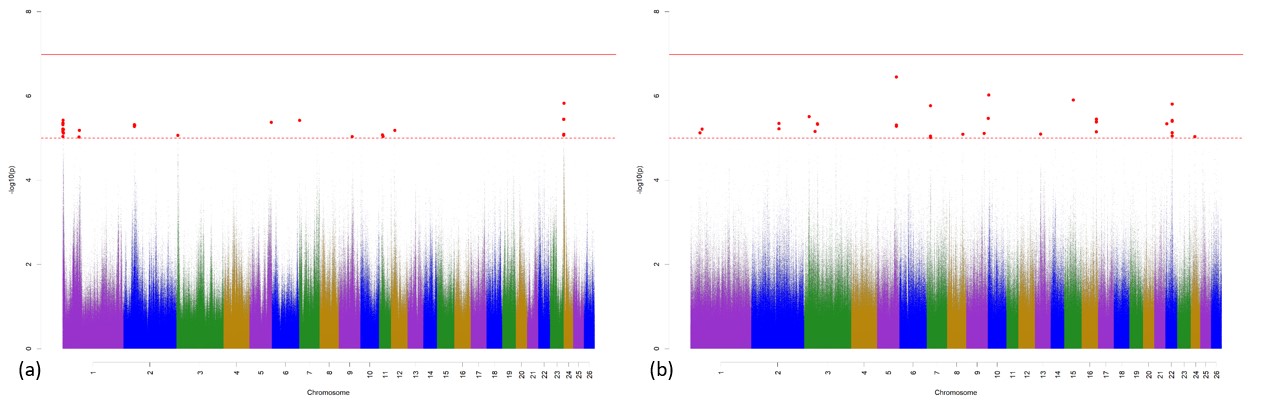

Supplement: Supplementary file 1 — Figures S1–S2. [file AGE-56-0-s003.docx]
