# Supplementary material for: Genetic parameters, genome‐wide associations and potential candidate genes for additive and dominance effects of tail traits in Merinoland sheep based on whole‐genome sequence data in a selection experiment
Source: Anim Genet. 2025 Sep 18;56(5):e70041. doi: 10.1111/age.70041 (PMC12445261; doi:10.1111/age.70041)
Supplement: Supplementary file 2 — Table S1. [file AGE-56-0-s001.docx]

**Supplementary table 1.** Candidate genes for additive-genetic effects for tail measurements (tail length (TL), tail circumference (TC)), body measurements (body length (BL), body weight (BW), and tail abnormalities (number of vertebrae (nVERT), axis deviation (AXISD), block vertebrae (BLCKV), wedged vertebrae (WDGV), fracture (FRC)).

| Trait | Gene symbol | NCBI gene ID | OAR | Gene position (bp) | SNP position (bp) | Function^1^ |
| --- | --- | --- | --- | --- | --- | --- |
| TL | *ZPLD1* | 101113639 | 1 | 167,803,417-167,853,202 | 167,835,854  167,837,083  167,842,977 | - |
|  | *INSR* | 443431 | 5 | 13,788,125-13,936,266 | 13,898,789 | Growth traits and (tail) fat deposition in Ethiopian indigenous sheep (Ahbara et al., 2019);  Litter size in Finnsheep and Hu sheep (Khaltabadi Farahani et al., 2020);  Embryonic development in mice (Louvi et al., 1997) |
|  | *EFCAB11* | 101119643 | 7 | 99,501,904-99,675,257 | 99,541,360  99,547,767  99,555,186  99,559,307  99,559,338  99,560,371  99,560,459 | Feed efficiency in Santa Inês sheep (Alvarenga et al., 2017);  Thigh and ham weight in pigs (Palombo et al., 2021; Zappaterra et al., 2021) |
|  | *PHACTR2* | 101118620 | 8 | 68,181,710-68,481,096 | 68,269,473 | - |
|  | *AATF* | 101109984 | 11 | 13,002,555-13,116,679 | 13,017,630  13,021,793  13,022,190  13,112,870  13,115,803 | - |
|  | *ACACA* | 443186 | 11 | 13,136,279-13,424,653 | 13,136,958 | Fat Tail deposition in sheep (Bakhtiarizadeh and Alanouti, 2020; ^a^Wang et al., 2022);  Fat metabolism (adipogenesis) in sheep (Dervishi et al., 2011);  Growth traits (body weight) and fat deposition in muscle tissue in Karachai goats (Selionova et al., 2022);  Milk quality (fatty acid metabolism) in sheep and goats (Moioli et al., 2007) |
|  | *FBXL20* | 101101999 | 11 | 39,486,651-39,585,097 | 39,555,125 | - |
|  | *CAMK1D* | 101108939 | 13 | 16,279,288-16,672,804 | 16,550,242 | Vertebral development in sheep (Li et al., 2018);  Osteoblast differentiation in mice (Wasserman et al., 2013) |
|  | *ASRGL1* | 100135685 | 21 | 37,286,133-37,308,758 | 37,305,347 | - |
|  | *PPP6R3* | 101113973 | 21 | 42,327,961-42,451,286 | 42,399,058  42,404,484  42,404,488  42,416,378 | Skeleton and cartilage development in horses (Pan et al., 2022);  Bone mineral density in humans (Medina-Gomez et al., 2017) |
|  | *CPT1A* | 443434 | 21 | 42,553,789-42,613,314 | 42,555,831  42,560,107  42,573,035  42,581,751  42,582,568  42,589,850  42,593,910  42,606,347  42,608,531  42,609,245 | Fat metabolism in sheep (Li et al., 2022);  Growth traits in goats (Li et al., 2019) |
|  | *IGHMBP2* | 101114988 | 21 | 42,654,335-42,680,243 | 42,656,699 | - |
|  | *SMIM38* | 121817506 | 21 | 42,860,774-42,870,227 | 42,862,493 | - |
|  | *PACS1* | 101104443 | 21 | 40,588,342-40,732,848 | 40,704,654 | - |
|  | *FANK1* | 101116440 | 22 | 44,882,616-44,996,266 | 44,929,206  44,937,479 | - |
|  | *DOCK1* | 101117205 | 22 | 45,861,626-46,430,644 | 46,235,128  46,236,427  46,250,654  46,263,273  46,402,603 | Muscle traits in sheep (Guðmundsdóttir, 2015);  Embryonic development (muscle tissue growth) in Mice (Laurin et al., 2008) |
|  | *EPB41L3* | 101106481 | 23 | 39,340,880-39,546,167 | 39,539,048  39,540,597  39,540,604  39,540,650  39,540,674  39,540,675  39,540,687 | - |
|  | *LOC105604472*  (uncharacterized) | 105604472 | 23 | 39,755,003-39,810,454 | 39,539,048  39,540,597  39,540,604  39,540,650  39,540,674  39,540,675  39,540,687  39,787,876  39,789,624  39,790,663  39,790,664  39,790,925  39,790,928 | - |
|  | *ARHGAP28* | 101102444 | 23 | 40,279,522-40,421,813 | 40,286,908  40,287,894  40,293,253 | - |
|  | *LOC121817770*  (uncharacterised) | 121817770 | 23 | 40,293,013-40,299,623 | 40,293,253 | - |
|  | *LAMA1* | 101102692 | 23 | 40,436,066-40,560,280 | 40,521,937  40,526,654  40,526,705 | Growth traits (birth weight) in sheep (Abousoliman et al., 2021) |
|  | *PTPRM* | 101102951 | 23 | 40,835,282-41,491,342 | 40,861,399  40,864,072  40,965,306  41,179,362  41,195,956  41,209,756  41,310,031  41,435,075 | Growth traits (birth weight) in sheep (Abousoliman et al., 2021) |
|  | *PIEZO2* | 101105203 | 23 | 42,710,021-42,967,602 | 42,860,908  42,862,154  42,956,385  42,958,011  42,959,931 | Bone development (osteoblast differentiation) in mice (Zhou et al., 2020);  Limb development in goats (Luigi-Sierra et al., 2020);  Arthrogryposis and Scoliosis in humans (Delle Vedove et al., 2016);  Distal arthrogryposis in humans (Coste et al., 2013) |
|  | *PRELID3A* | 101109137 | 23 | 43,346,324-43,397,506 | 43,355,674 |  |
|  | *SPIRE1* | 101106482 | 23 | 43,395,568-43,537,954 | 43,451,253  43,516,929 | Body weight and body conformation traits in Karachai goats (Selionova et al., 2022) |
|  | *RNMT* | 101110469 | 23 | 43,976,087-44,009,298 | 43,994,438 | - |
| TC | *CNTNAP5* | 101116541 | 2 | 189,526,480-190,612,887 | 189,736,517 | Body conformation traits (body length) in cattle (Vanvanhossou et al., 2020);  Hip cross height in Brahman cattle (^b^Chen et al., 2020);  Body conformation traits in goats (Rahmatalla et al., 2014);  Growth traits in pigs (Puig-Oliveras et al., 2014) |
|  | *ZNF804A* | 101116539 | 2 | 124,682,478-125,033,650 | 124,919,790  124,919,791  124,919,795 | - |
|  | *EXOC6B* | 101111176 | 3 | 94,125,397-94,833,697 | 94,179,934 | Body conformation traits (metacarpal length) in sheep (James et al., 2022);  Skeletal malformations of the limbs (spondyloepimetaphyseal dysplasia) in humans (Campos-Xavier et al., 2018) |
|  | *COL25A1* | 101120576 | 6 | 16,363,707-16,859,020 | 16,540,904 | - |
|  | *DKK2* | 101123633 | 6 | 18,492,309-18,618,733 | 18,582,817 | - |
|  | *NPNT* | 101102901 | 6 | 19,569,046-19,646,418 | 19,611,100 | Osteoblast differentiation in mice (Kahai et al., 2009);  Osteoblast differentiation and mineralization, osteogenic angiogenesis in mice (Sun et al., 2018) |
|  | *GSTCD* | 101103813 | 6 | 19,692,829-19,839,711 | 19,722,042  19,722,089  19,722,105  19,722,962 | Fatty acid metabolism in sheep (Gunawan et al., 2021) |
|  | *ASCC3* | 101123305 | 8 | 36,369,002-36,710,267 | 36,484,149 | Growth and carcass traits (backfat thickness) in pigs (Wei et al., 2023) |
|  | *KLHL28* | 101105190 | 18 | 52,514,013-52,550,629 | 52,528,583 | - |
|  | *OPCML* | 101117888 | 21 | 30,857,247-31,902,052 | 31,591,118 | - |
|  | *PRKG1* | 443010 | 22 | 6,687,943-8,080,976 | 7,900,325 | - |
| BL | *LOC121819679*  (uncharacterised) | 121819679 | 5 | 90,707,564-90,742,522 | 90,735,266 | - |
|  | *FAM172A* | 101122278 | 5 | 90,726,962-91,134,007 | 90,735,266 | - |
|  | *PCSK1* | 443029 | 5 | 93,366,210-93,418,530 | 93,370,089  93,370,122  93,370,182  93,370,186  93,370,187  93,370,202  93,370,208  93,370,239  93,370,242  93,370,332  93,370,596  93,370,607  93,370,763  93,370,816  93,370,832  93,371,281  93,371,467  93,373,282  93,379,588  93,384,010  93,395,447 | Growth traits (body length, chest circumference) in cattle (Sun et al., 2015);  Growth traits (birth weight) in cattle (Shan et al., 2010);  Fat deposition and production traits (growth performance, backfat thickness, average daily gain) in pigs (Fontanesi et al., 2012) |
|  | *LOC101120092* | 101120092 | 10 | 2,249,108-2,363,880 | 2,322,591 | - |
|  | *OPCML* | 101117888 | 21 | 30,857,247-31,902,052 | 31,791,964 | Spine maturation in mice (^b^Zhang et al., 2019) |
| BW | *COL11A1* | 101117548 | 1 | 80,145,966-80,371,232 | 80,272,224  80,272,225 | Meat tenderness in cattle (Dinh et al., 2021);  Adult height in humans (Shen et al., 2016) |
|  | *HDAC4* | 101108012 | 1 | 2,120,918-2,410,934 | 2,122,306  2,122,640  2,123,588  2,128,964  2,132,243  2,136,096 | Regulation of muscle growth and development in goats (Sui et al., 2019);  Skeletal muscle growth and development in chicken (Zhao et al., 2020);  Muscle regeneration in mice (Renzini et al., 2018) |
|  | *LRRC8A* | 101117230 | 3 | 7,496,776-7,524,523 | 7,513,242 | - |
|  | *LOC101105161*  (*NREP*) | 101105161 | 7 | 2,142,429-2,175,276 | 2,148,294 | - |
|  | *HNF1B* | 443198 | 11 | 13,675,334-13,738,334 | 13,693,176 | - |
|  | *LOC114112008* | 114112008 | 24 | 128,987-133,732 | 128,687 | - |
|  | *SOX8* | 101106132 | 24 | 1,320,938-1,325,831 | 1,325,833 | Growth traits (withers height, chest girth) in yaks (Zhang et al., 2022);  Regulation of bone formation in mice (Schmidt et al., 2005) |
| nVERT | *MAN1A1* | 101103326 | 8 | 18,939,969-19,111,314 | 19,006,803 | Body weight in Baluchi sheep (Gholizadeh et al., 2015);  Body weight and body conformation traits in Luzhong Mutton sheep (Tao et al., 2020) |
|  | *MCM9* | 101123472 | 8 | 19,365,239-19,453,469 | 19,390,828 | Body weight and body conformation traits in Luzhong Mutton sheep (Tao et al., 2020) |
|  | *LOC114110481*  (uncharacterised) | 114110481 | 23 | 40,559,628-40,570,067 | 40,568,574 | - |
| AXISD | *LOC106990799* | 106990799 | 1 | 88,819,385-88,831,604 | 88,823,845 | - |
|  | *DCBLD2* | 101107057 | 1 | 164,028,356-164,112,229 | 164,082,021 | Fat tail deposition in sheep (Mastrangelo et al., 2019) |
|  | *LOC101102199* | 101102199 | 1 | 83,945,152-83,995,645 | 83,987,791 | - |
|  | *STXBP3* | 101122397 | 1 | 86,084,081-86,130,764 | 86,094,373 | - |
|  | *RAPGEF4* | 101112196 | 2 | 136,857,957-137,186,679 | 136,963,847  136,963,880  136,963,881 | Fat tail deposition in sheep (Yuan et al., 2016) |
|  | *MSANTD3* | 105608992 | 2 | 22,686,670-22,716,364 | 22,704,789 | Growth traits (yearling weight) in cattle (Mkize et al., 2023) |
|  | *C2CD5* | 101116387 | 3 | 193,251,622-193,339,539 | 193,293,265 | Wool traits in sheep (Ramos et al., 2023) |
|  | *CRACD*  (*KIAA1211*) | 101108572 | 6 | 72,183,595-72,472,783 | 72,289,077  72,398,363  72,432,936 | - |
|  | *RBM47* | 101115877 | 6 | 60,111,604-60,281,254 | 60,275,400 | Growth in sheep (Yurchenko et al., 2019) |
|  | *WDR19* | 101113150 | 6 | 59,082,949-59,177,019 | 59,133,905 | - |
|  | *GABRB1* | 101122358 | 6 | 66,336,302-66,776,044 | 66,715,955  66,756,422  66,759,092 | - |
|  | *FOXN3* | 101114708 | 7 | 98,859,775-99,307,627 | 99,061,037 | Productivity traits in sheep (Krivoruchko et al., 2021) |
|  | *MAP3K4* | 101113246 | 8 | 84,418,003-84,524,485 | 84,432,236 | Fat tail development in sheep (Bakhtiarizadeh et al., 2024);  Hepatic lipid metabolism in humans (He et al., 2022) |
|  | *SPATA17* | 101121111 | 12 | 20,350,132-20,598,752 | 20,417,780 | Growth and development traits (chest circumference) in sheep (Tuersuntuoheti et al., 2022) |
|  | *PRIM2* | 101106040 | 20 | 2,741,395-3,045,123 | 3,013,558 | - |
| BLCKV | *CADM2* | 101120371 | 1 | 153,842,363-155,133,810 | 154,084,965 | Body size traits (body height) in sheep (Jiang et al., 2021);  Growth traits (body weight) in yaks (Ge et al., 2019) |
|  | *ATP13A5* | 101107321 | 1 | 194,858,634-194,975,190 | 194,888,117 | - |
|  | *LOC114108764* | 114108764 | 3 | 208,167,527-208,550,471 | 208,325,184 | - |
|  | *LOC105614851* | 105614851 | 3 | 208,324,835-208,383,801 | 208,325,184 | - |
|  | *NXPH1* | 101121848 | 4 | 17,300,516-17,665,790 | 17,426,814 | - |
|  | *BDH2* | 101106011 | 6 | 22,094,624-22,123,462 | 22,119,408 | - |
|  | *TACR3* | 101121604 | 6 | 21,712,367-21,793,451 | 21,789,444 | - |
|  | *NRXN3* | 101116577 | 7 | 87,447,430-89,290,222 | 89,263,820 | - |
|  | *LOC101112162* | 101112162 | 16 | 7,007,705-7,039,835 | 7,036,863 | - |
|  | *PRKG1* | 443010 | 22 | 6,687,943-8,080,976 | 6,996,983 | - |
|  | *ATP9B* | 101108356 | 23 | 1,047,116-1,203,681 | 1,130,364 | Regulation of osteoarthritis in Mice (Zhou et al., 2018) |
|  | *KLHL14* | 101117636 | 23 | 24,716,862-24,830,407 | 24,748,055 | Bone strength in chickens (Yue et al., 2022) |
|  | *LOC121817702*  (uncharacterised) | 121817702 | 23 | 24,842,259-24,895,346 | 24,872,721 | - |
|  | *DOK6* | 101112783 | 23 | 7,632,803-8,038,013 | 7,891,463  7,891,482  7,891,486  7,998,942 | - |
|  | *CCDC102B* | 101103523 | 23 | 8,346,709-8,637,982 | 8,439,921 | - |
|  | *ZNF25* | 105604930 | 25 | 12,319,433-12,347,314 | 12,321,215  12,347,519 | Osteoblast differentiation in humans (Twine et al., 2016);  Yearling weight in sheep (Lu et al., 2020) |
|  | *ANK3* | 101120296 | 25 | 15019554-15,775,015 | 15,417,105 | - |
| WDGV | *ZMYM6* | 101108433 | 1 | 10,034,945- 10,081,252 | 10,081,864 | - |
|  | *EXO1*  (*HEX1, hExol*) | 101120258 | 12 | 34,231,773-34,275,752 | 34,261,389 | Bone health (osteogenesis) in humans (^a^Chen et al., 2020) |
|  | *WDR64* | 101120508 | 12 | 34,286,142-34,421,065 | 34,415,198 | - |
|  | *RGS7* | 101121279 | 12 | 34,682,357-35,155,100 | 34,833,230  34,833,273  35,113,679  35,152,542  35,152,546 | Bone mineral density in humans (^a^He et al., 2023);  Chondrogenic differentiation in humans (Appleton et al., 2006) |
|  | *NLRP13* | 101109118 | 14 | 64,110,668-64,134,880 | 64,112,726  64,113,935 | - |
|  | *CLUL1* | 101103443 | 23 | 35,967,660-35,991,671 | 35,992,193 | - |
|  | *ZMIZ1* | 101111251 | 25 | 33,943,007-34,133,057 | 34,035,441  34,035,442 | Distal skeleton anomalies in humans (^b^He et al., 2023; Lu et al., 2022);  Axial spondyloarthritis in humans (Layh-Schmitt et al., 2017) |
|  | *SH2D4B* | 101109766 | 25 | 34,962,083-35,040,911 | 35,030,663 | - |
|  | *NRG3* | 101112537 | 25 | 35,919,548-37,160,225 | 36,577,876  36,577,895  36,580,715  36,580,761  36,583,354  37,150,562 | Angular limb deformity in sheep (Becker et al., 2023) |
|  | *CCSER2* | 101110815 | 25 | 38,308,913-38,468,877 | 38,374,023 | Muscle fiber characteristics (number of muscle fibers) in pigs (^b^Zhang et al., 2020) |
|  | *SHLD2* | 101113562 | 25 | 40,679,986-40,783,361 | 40,706,645 | Growth traits (eaning weight) in sheep (Abousoliman et al., 2021) |
| FRC | *LOC114113604*  (uncharacterised) | 114113604 | 3 | 76,549,727-76,551,119 | 76,550,039 | - |
|  | *SGSM1* | 101116605 | 17 | 65,200,637-65,276,700 | 65,253,189  65,253,194 | Growth traits in pigs (^c^Wang et al., 2022; Zhou et al., 2019);  Growth traits in dogs (Antkowiak and Szydlowski, 2023) |

^1^No function: no function relevant to the study.

**References**

Abousoliman I, Reyer H, Oster M, Murani E, Mohamed I, Wimmers K (2021) Genome-wide analysis for early growth-related traits of the locally adapted Egyptian Barki sheep. Genes 12. https://doi.org/10.3390/genes12081243

Ahbara A, Bahbahani H, Almathen F, Al Abri M, Agoub MO, Abeba A, Kabede A, Musa HH, Mastrangelo S, Pilla F, Ciani E, Hanotte O, Mwacharo JM (2019) Genome-wide variation, candidate regions and genes associated with fat deposition and tail morphology in Ethiopian indigenous sheep. Frontiers in Genetics 9:699. https://doi.org/10.3389/fgene.2018.00699

Alvarenga AB (2017) Feed efficiency traits in Santa Inês sheep under genomic approaches. Universidade de São Paulo. https://doi.org/10.11606/D.11.2018.tde-20032018-160145

Antkowiak M and Szydlowski M (2023) Uncovering structural variants associated with body weight and obesity risk in labrador retrievers: a genome-wide study. Front Genet 14. https://doi.org/10.3389/fgene.2023.1235821

Appleton CTG, James CG, Beier F (2006) Regulator of G‐protein signaling (RGS) proteins differentially control chondrocyte differentiation. Journal of Cellular Physiology 207:735-745. https://doi.org/10.1002/jcp.20615

Bakhtiarizadeh MR and Alamouti AA (2020) RNA‑Seq based genetic variant discovery provides new insights into controlling fat deposition in the tail of sheep. Scientific Reports 10. https://doi.org/10.1038/s41598-020-70527-8

Bakhtiarizadeh MR (2024) Deciphering the role of alternative splicing as a potential regulator in fat-tail development of sheep: a comprehensive RNA-seq based study. Scientific Reports 14:2361. https://doi.org/10.1038/s41598-024-52855-1

Becker GM, Shira KA, Woods JL, Khilji SF, Schauer CS, Webb BT, Stewart WC, Murdoch BM (2023) Angular limb deformity associated with TSPAN18, NRG3 and NOVA2 in Rambouillet rams. Scientific Reports 13. https://doi.org/10.1038/s41598-023-43320-6

Campos‐Xavier B, Rogers RC, Niel‐Bütschi F, Ferreira C, Unger S, Spranger J, Superti‐Furga A (2018) Confirmation of spondylo‐epi‐metaphyseal dysplasia with joint laxity, EXOC6B type. American Journal of Medical Genetics Part A 176:2934-2935. https://doi.org/10.1002/ajmg.a.40631

Chen Q, Huang B, Zhan J, Wang J, Qu K, Zhang F, Shen J, Jia P, Ning Q, Zhang J, Chen N, Chen H, Lei C (2020a) Whole-genome analyses identify loci and selective signals associated with body size in cattle. Journal of Animal Science 98. https://doi.org/10.1093/jas/skaa068

Chen YS, Kang XR, Zhou ZH, Yang J, Xin Q, Ying CT, Zhang YP, Tao J (2020b) MiR-1908/EXO1 and MiR-203a/FOS, regulated by scd1, are associated with fracture risk and bone health in postmenopausal diabetic women. Aging (Albany NY) 12. https://doi.org/10.18632/aging.103227

Coste B, Houge G, Murray MF, Stietziel N, Bandell M, Giovanni MA, Philippakis A, Hoischen A, Riemer G, Steen U, Steen VM, Mathur J, Cox J, Lebo M, Rehm H, Weiss ST, Wood JN, Maas RL, Sunyaev SR, Patapoutian A (2013) Gain-of-function mutations in the mechanically activated ion channel PIEZO2 cause a subtype of Distal Arthrogryposis. PNAS 110:4667-4672. https://doi.org/10.1073/pnas.1221400110

Delle Vedove A, Storbeck M, Heller R, Hölker I, Hebbar M, Shukla A, Magnusson O, Cirak S, Girisha KM, O´Driscoll M, Loeys B, Wirth B (2016) Biallelic loss of proprioception-related PIEZO2 causes muscular atrophy with perinatal respiratory distress, arthrogryposis, and scoliosis. The American Journal of Human Genetics 99:1206-1216. http://dx.doi.org/10.1016/j.ajhg.2016.09.019

Dervishi E, Serrano C, Joy M, Serrano M, Rodellar C, Calvo JH (2011) The effect of feeding system in the expression of genes related with fat metabolism in semitendinous muscle in sheep. Meat Science 89:91-97. https://doi.org/10.1016/j.meatsci.2011.04.003

Dinh PTN, Chung Y, Lee DJ, Kang DH, Park BH, Chung KY, Lee SH (2021) Association Study of Functional Candidate Genes for Meat Tenderness in Hanwoo Cattle. Journal of Animal Breeding and Genomics 5:181-189. https://doi.org/10.12972/jabng.20210017

Fontanesi L, Bertolini F, Scotti E, Trevisi P, Buttazzoni L, Dall'Olio S, Davoli R, Bosi P, Russo V (2012) Polymorphisms in an obesity-related gene (PCSK1) are associated with fat deposition and production traits in Italian heavy pigs. Animal 6:1913-1924. https://doi.org/10.1017/S1751731112001280

Ge F, Jia C, Chu M, Liang C, Yan P (2019) Copy number variation of the CADM2 gene and its association with growth traits in yak. Animals 9. https://doi.org/10.3390/ani9121008

Gholizadeh M, Rahimi-Mianji G, Nejati-Javaremi A (2015) Genomewide association study of body weight traits in Baluchi sheep. J Genet 94:143–146. https://doi.org/10.1007/s12041-015-0469-1

Guðmundsdóttir ÓÓ (2015) Genome-wide association study of muscle traits in Icelandic sheep (Doctoral dissertation)

Gunawan A, Listyarini K, Harahap RS, Jakaria, Roosita K, Sumantri C, Inounu I, Akter SH, Islam A, Uddin MJ (2021) Hepatic transcriptome analysis identifies genes, polymorphisms and pathways involved in the fatty acids metabolism in sheep. PLoS One 16. https://doi.org/10.1371/journal.pone.0260514

He Z, Bin Y, Chen G, Li Q, Fan W, Ma Y, Yi J, Luo X, Tan Z, Li J (2022) Identification of MAP3K4 as a novel regulation factor of hepatic lipid metabolism in non-alcoholic fatty liver disease. Journal of Translational Medicine 20. https://doi.org/10.1186/s12967-022-03734-8

He L, Wang Y, Pan J, Guo L, Zhou H, Zhang L (2023a) Clinical report and genetic analysis of a novel variant in ZMIZ1 causing neurodevelopmental disorder with dysmorphic factors and distal skeletal anomalies in a Chinese family. Genes Genom. https://doi.org/10.1007/s13258-023-01480-9

He D, Liu H, Wei W, Zhao Y, Cai Q, Shi S, Chu X, Qin X, Zhang N, Xu P, Zhang F (2023b) A longitudinal genome‑wide association study of bone mineral density mean and variability in the UK Biobank. Osteoporosis International 34:1907-1916. https://doi.org/10.1007/s00198-023-06852-1

James C, Pemberton JM, Navarro P, Knott S (2022) The impact of SNP density on quantitative genetic analyses of body size traits in a wild population of Soay sheep. Ecol Evol 12. https://doi.org/10.1002/ece3.9639

Jiang J, Cao Y, Shan H, Wu J, Song X, Jiang Y (2021) The GWAS analysis of body size and population verification of related SNPs in Hu sheep. Front Genet 12. https://doi.org/10.3389/fgene.2021.642552

Kahai S, Lee SC, Seth A, Yang BB (2010) Nephronectin promotes osteoblast differentiation via the epidermal growth factor-like repeats. FEBS Letters 584:233-238. https://doi.org/10.1016/j.febslet.2009.11.077

Khaltabadi Farahani AH, Mohammadi H, Moradi H (2020) Gene set enrichment analysis using genome-wide association study to identify genes and pathways associated with litter size in various sheep breeds. Animal Production 22:325-335. https://doi.org/10.22059/jap.2020.292715.623468

Krivoruchko A, Sermyagin A, Saprikina T, Golovanova N, Kvochko A, Yatsyk O (2021) Genome wide associations study of single nucleotide polymorphisms with productivity parameters in Jalgin merino for identification of new candidate genes. Gene Reports 23. https://doi.org/10.1016/j.genrep.2021.101065

Laurin M, Fradet N, Blangy A, Hall A, Vuori K, Côté JF (2008) The atypical Rac activator Dock180 (Dock1) regulates myoblast fusion in vivo. Proceedings of the National Academy of Sciences 105:15446-15451. https://doi.org/10.1073/pnas.0805546105

Layh-Schmitt G, Lu S, Navid F, Brooks SR, Lazowick E, Davis KM, Montagna C, Gadina M, Colbert RA (2017) Generation and differentiation of induced pluripotent stem cells reveal ankylosing spondylitis risk gene expression in bone progenitors. Clinical Rheumatology 36:143-154. https://doi.org/10.1007/s10067-016-3469-5

Li S, Luo R, Lai D, Ma, M, Hao F, Qi X, Liu X, Liu D (2018) Whole-genome resequencing of Ujumqin sheep to investigate the determinants of the multi-vertebral trait. Genome 61:653-661. https://doi.org/10.1139/gen-2017-0267

Li WY, Liu Y, Gao CF, Lan XY, Wu XF (2019) A novel duplicated insertion/deletion (InDel) of the CPT1a gene and its effects on growth traits in goat. Anim Biotechnol 32:343-351. https://doi.org/10.1080/10495398.2019.1698433

Li T, Jin M, Fei X, Yuan Z, Wang Y, Quan K, Wang T, Yang J, He M, Wei C (2022) Transcriptome comparison reveals the difference in liver fat metabolism between different sheep breeds. Animals 12. https://doi.org/10.3390/ani12131650

Louvi A, Accili D, Efstratiadis A (1997) Growth-promoting interaction of IGF-II with the insulin receptor during mouse embryonic development. Developmental Biology 189(1)33-48. https://doi.org/10.1006/dbio.1997.8666

Lu Z, Yue Y, Yuan C, Liu J, Chen Z, Niu C, Sun X, Zhu S, Zhao H, Guo T, Yang B (2020) Genome-wide association study of body weight traits in chinese fine-wool sheep. Animals 10. https://doi.org/10.3390/ani10010170

Lu G, Ma L, Xu P, Xian B, Wu L, Ding J, He X, Xia H, Ding W, Yang Z, Peng, Q (2022) A de novo ZMIZ1 pathogenic variant for neurodevelopmental disorder with dysmorphic facies and distal skeletal anomalies. Frontiers in Genetics 13 :840577. https://doi.org/10.3389/fgene.2022.840577

Luigi-Sierra MG, Landi V, Guan D, Delgado JV, Castelló A, Cabrera B, Mármol-Sánchez E, Alvarez JF, Gómez-Carpio M, Martínez A, Such X, Jordana J, Amills M (2020) A genome-wide association analysis for body, udder, and leg conformation traits recorded in Murciano-Granadina goats. Journal of Dairy Science, 103:11605-11617. https://doi.org/10.3168/jds.2020-18461

Mastrangelo S, Moioli B, Ahbara A, Latairish S, Pportolano B, Pilla E, Ciani E (2019) Genome-wide scan of fat-tail sheep identifies signals of selection for fat deposition and adaptation. Anim Prod Sci 59:835-848. https://doi.org/10.1071/AN17753

Medina-Gomez C, Kemp JP, Dimou NL, Kreiner E, Chesi A, Zemel BS et al. (2017) Bivariate genome-wide association meta-analysis of pediatric musculoskeletal traits reveals pleiotropic effects at the SREBF1/TOM1L2 locus. Nature communications 8:121. https://doi.org/10.1038/s41467-017-00108-3

Mkize N and Maiwashe A (2023) The Identification of Genomic Regions Associated with Tick Count, Growth Traits, and Skin Thickness in F2 Angus x Nguni Cattle Using Genome-Wide Association Analysis

Moioli B, D’Andrea M, Pilla FJSRR (2007) Candidate genes affecting sheep and goat milk quality. Small Ruminant Research 68:179-192. https://doi.org/10.1016/j.smallrumres.2006.09.008

Palombo V, D’Andrea M, Licastro D, Dal Monego S, Sgorlon S, Sandri M, Stefanon B (2021) Single-step genome wide association study identifies QTL signals for untrimmed and trimmed thigh weight in Italian crossbred pigs for dry-cured ham production. Animals 11. https://doi.org/10.3390/ani11061612

Pan J, Purev C, Zhao H, Zhang Z, Wang F, Wendoule N, Qi G, Liu Y, Zhou H (2022) Discovery of exercise-related genes and pathway analysis based on comparative genomes of Mongolian originated Abaga and Wushen horse. Open Life Sciences 17:1269-1281. https://doi.org/10.1515/biol-2022-0487

Puig-Oliveras A, Ballester M, Corominas J, Revilla M, Estellé J, Fernandez AI, Ramayo-Caldas Y, Folch JM (2014) A co-association network analysis of the genetic determination of pig conformation, growth and fatness. PLoS One 9. https://doi.org/10.1371/journal.pone.0114862

Rahmatalla SA, Arends D, Reissmann M, Wimmers K, Reyer H, Brockmann GA (2018) Genome‐wide association study of body morphological traits in Sudanese goats. Animal Genetics 49:478-482. https://doi.org/10.1111/age.12686

Ramos Z, Garrick DJ, Blair HT, Vera B, Ciappesoni G, Kenyon PR (2023). Genomic regions associated with wool, growth and reproduction traits in Uruguayan Merino sheep. Genes 14. https://doi.org/10.3390/genes14010167

Renzini A, Marroncelli N, Noviello C, Moresi V, Adamo S (2018) HDAC4 regulates skeletal muscle regeneration via soluble factors. Frontiers in Physiology 9. https://doi.org/10.3389/fphys.2018.01387

Schmidt K, Schinke T, Haberland M, Priemel M, Schilling AF, Mueldner C, Rueger JM, Sock E, Wegner M, Amling M (2005) The high mobility group transcription factor Sox8 is a negative regulator of osteoblast differentiation. The Journal of Cell Biology 168:899-910. https://doi.org/10.1083/jcb.200408013

Selionova M, Aibazov M, Mamontova T, Malorodov V, Sermyagin A, Zinovyeva N, Easa AA (2022) Genome-wide association study of live body weight and body conformation traits in young Karachai goats. Small Ruminant Research 216:106836. https://doi.org/10.1016/j.smallrumres.2022.106836

Shan L, Sun J, Zhang C, Fang X, Lei C, Lan X, Chen H (2011) The polymorphisms of bovine PCSK1 gene and their associations with growth traits. Genes & Genomics 33:57-63. https://doi.org/10.1007/s13258-010-0049-y

Shen C, Zheng X, Gao J, Zhu C, Ko R, Tang X, Yang C, Dou J, Lin Y, Cheng Y, Liu L, Xu S, Chen G, Zuo X, Yin X, Sun L, Cui Y, Yang S, Zhang X, Zhou F (2016) A rare variant in COL11A1 is strongly associated with adult height in Chinese Han population. Journal of Genetics and Genomics 45:549-554. https://doi.org/10.1016/j.jgg.2016.04.002

Sui M, Zheng Q, Wu H, Zhu L, Ling Y, Wang L, Fang F, Liu Y, Zhang Z, Chu M, Zhang Y (2020) The expression and regulation of miR-1 in goat skeletal muscle and satellite cell during muscle growth and development. Animal Biotechnology 31:455-462. https://doi.org/10.1080/10495398.2019.1622555

Sun J, Shan L, Zhang C, Chen H (2015) Haplotype combination of the bovine PCSK1 gene sequence variants and association with growth traits in Jiaxian cattle. Journal of Genetics 94:123-129. https://doi.org/10.1007/s12041-014-0440-6

Sun Y, Kuek V, Qiu H, Tickner J, Chen L, Wang H, He W, Xu J (2018) The emerging role of NPNT in tissue injury repair and bone homeostasis. Journal of Cellular Physiology 233:1887-1894. https://doi.org/10.1002/jcp.26013

Tao L, He XY, Pan LX, Wang JW, Gan SQ, Chu MX (2020) Genome-wide association study of body weight and conformation traits in neonatal sheep. Anim Genet 51:336-340. https://doi.org/10.1111/age.12904

Tuersuntuoheti M, Zhang J, Zhou W, Zhang CL, Liu C, Chang Q, Liu S (2023) Exploring the growth trait molecular markers in two sheep breeds based on Genome-wide association analysis. PLoS One 18. https://doi.org/10.1371/journal.pone.0283383

Twine NA, Harkness L, Kassem M, Wilkins MR (2016) Transcription factor ZNF25 is associated with osteoblast differentiation of human skeletal stem cells. BMC Genomics 17. https://doi.org/10.1186/s12864-016-3214-0

Vanvanhossou SFU, Scheper C, Dossa LH, Yin T, Brügemann K, König S (2020) A multi-breed GWAS for morphometric traits in four Beninese indigenous cattle breeds reveals loci associated with conformation, carcass and adaptive traits. BMC Genomics 21. https://doi.org/10.1186/s12864-020-07170-0

Wang Q, Cao H, Su X, Liu W (2022a) Identification of key miRNAs regulating fat metabolism based on RNA-seq from fat-tailed sheep and F2 of wild Argali. Gene 834. https://doi.org/10.1016/j.gene.2022.146660

Wang JJ, Li ZD, Zheng LQ, Zhang T, Shen W, Lei CZ (2022c) Genome-wide detection of selective signals for fecundity traits in goats (Capra hircus). Gene 818. https://doi.org/10.1016/j.gene.2022.146221

Wasserman E, Webster D, Kuhn G, Attar-Namdar M, Müller R, Bab I (2013) Differential load-regulated global gene expression in mouse trabecular osteocytes. Bone 53:14-23. https://doi.org/10.1016/j.bone.2012.11.017

Wei C, Zeng H, Zhong Z, Cai X, Teng J, Liu Y, Zhao Y, Wu X, Li J, Zhang Z (2023) Integration of non-additive genome-wide association study with a multi-tissue transcriptome analysis of growth and carcass traits in Duroc pigs. Animal 17. https://doi.org/10.1016/j.animal.2023.100817

Yuan Z, Liu Z, Kijas JW, Zhu C, Hu S, Ma X, Zhang L, Du L, Wang H, Wei C (2016) Selection signature analysis reveals genes associated with tail type in Chinese indigenous sheep. Anim Genet 48:55-66. https://doi.org/10.1111/age.12477

Yue Q, Chen Y, Chen H, Zhou R (2022) Transcriptome profile reveals novel candidate genes associated with bone strength in end-of-lay hens. Animal Biotechnology 34:3099-3107. https://doi.org/10.1080/10495398.2022.2134884

Yurchenko AA, Deniskova TE, Yudin NS, Dotsev AV, Khamiruev TN, Selionova MI, Egorov SV, Reyer H, Wimmers K, Brem G, Zinovieva NA, Larkin DM (2019) High-density genotyping reveals signatures of selection related to acclimation and economically important traits in 15 local sheep breeds from Russia. BMC Genomics 20:1-19. https://doi.org/10.1186/s12864-019-5537-0

Zappaterra M, Zambonelli P, Schivazappa C, Simoncini N, Virgili R, Stefanon B, Davoli R (2021) Investigating the features of PDO green hams during salting: Insights for new markers and genomic regions in commercial hybrid pigs. Animals 11. https://doi.org/10.3390/ani11010068

Zhang T, Gao H, Sahana G, Zan Y, Fan H, Liu J, Shi L, Wang H, Du L, Wang L, Zhao F (2019b) Genome‐wide association studies revealed candidate genes for tail fat deposition and body size in the Hulun Buir sheep. Journal of Animal Breeding and Genetics 136:362-370. https://doi.org/10.1111/jbg.12402

Zhang L, Guo Y, Wang L, Liu X, Yan H, Gao H., Hou X, Zhang Y, Guo H, Yue J, An J, Wang L (2020b) Genomic variants associated with the number and diameter of muscle fibers in pigs as revealed by a genome-wide association study. Animal 14:475-481. 10.1017/S1751731119002374

Zhang Z, Chu M, Bao Q, Bao P, Guo X, Liang C, Yan P (2022) Two different copy number variations of the SOX5 and SOX8 genes in yak and their association with growth traits. Animals 12. https://doi.org/10.3390/ani12121587

Zhao J, Shen X, Cao X, He H, Han S, Chen Y, Cui C, Wei Y, Wang Y, Li D, Zhu Q, Yin H (2020) Hdac4 regulates the proliferation, differentiation and apoptosis of chicken skeletal muscle satellite cells. Animals 10. https://doi.org/10.3390/ani10010084

Zhou ZB, Du D, Huang GX, Chen A, Zhu L (2018) Circular RNA Atp9b, a competing endogenous RNA, regulates the progression of osteoarthritis by targeting miR-138-5p. Gene 646:203-209. https://doi.org/10.1016/j.gene.2017.12.064

Zhou L, Zhao W, Fu Y, Fang X, Ren S, Ren J (2019) Genome‐wide detection of genetic loci and candidate genes for teat number and body conformation traits at birth in Chinese Sushan pigs. Anim Genet 50: 753-756. https://doi.org/10.1111/age.12844

Zhou T, Gao B, Fan Y, Liu Y, Feng S, Cong Q, Zhang X, Zhou Y, Yadav PS, Lin J, Wu N, Zhao L, Huang D, Zhou S, Su P, Yang Y (2020) Piezo1/2 mediate mechanotransduction essential for bone formation through concerted activation of NFAT-YAP1-ß-catenin. eLife 9. https://doi.org/10.7554/eLife.52779
