# Supplementary material for: Genetic parameters, genome‐wide associations and potential candidate genes for additive and dominance effects of tail traits in Merinoland sheep based on whole‐genome sequence data in a selection experiment
Source: Anim Genet. 2025 Sep 18;56(5):e70041. doi: 10.1111/age.70041 (PMC12445261; doi:10.1111/age.70041)
Supplement: Supplementary file 3 — Table S2. [file AGE-56-0-s002.docx]

**Supplementary table 2.** Candidate genes for dominance effects for tail measurements (tail length (TL), tail circumference (TC)), body measurements (body length (BL), body weight (BW), and tail abnormalities (number of vertebrae (nVERT), axis deviation (AXISD), block vertebrae (BLCKV), wedged vertebrae (WDGV), fracture (FRC)).

| Trait | Gene symbol | NCBI gene ID | OAR | Gene position (bp) | SNP position (bp) | Function^1^ |
| --- | --- | --- | --- | --- | --- | --- |
| TL | *SCARA3* | 101107766 | 2 | 102,048,891-102,083,829 | 102,069,857 | Milk yield in buffalos (Deng et al., 2019) |
|  | *DNER* | 101119703 | 2 | 231,387,211-231,765,997  231,387,211-231,765,997 | 231,391,913  231,464,813 | Production traits in Romanov sheep (Krivoruchko et al., 2021) |
|  | *DENND1A* | 101113668 | 3 | 11,836,438-12,372,699 | 12,194,062 | Fertility traits in sheep and cows (Liu et al., 2022a; Zheng et al., 2021) |
|  | *CFAP36* | 101121587 | 3 | 68,523,662-68,553,690 | 68,554,588 | - |
|  | *CTNNA2* | 101121501 | 3 | 50,614,437-52,019,005 | 51,455,511 | Milk fat yield in dairy cattle (Cai et al., 2018; Nayak et al., 2023) |
|  | *LOC105605798*  (*ANKRD26*) | 105605798 | 12 | 6,686,174-6,764,917 | 6,756,435 | Body conformation traits (pin width) in dromedaries (Sani et al., 2023);  Gigantism in mice (Bera et al., 2008) |
|  | *LOC114117597* | 114117597 | 13 | 52,745,286-52,828,192 | 52,781,909 | - |
|  | *PARP8* | 101120603 | 16 | 28,476,208-28,668,014 | 28,493,552 | Fertility traits (pregnancy rate) in sheep (Ramos et al., 2023) |
|  | *HCN1* | 101115314 | 16 | 29,254,337-29,705,331 | 29,412,128 | - |
|  | *GRK3* | 101117366 | 17 | 65,579,987-65,696,519  65,579,987-65,696,519  65,579,987-65,696,519  65,579,987-65,696,519  65,579,987-65,696,519 | 65,643,066  65,643,099  65,643,648  65,644,856  65,646,403 | Regulation of osteoclast differentiation in mice (Rabjohns et al., 2023) |
|  | *MYO18B* | 101117620 | 17 | 65,700,568-65,942,641 | 65,893,350 | Skeletal muscle development in yaks (^a^Wu et al., 2022);  Cardiac muscle tissue development in sheep and mice (Ajima et al., 2008; Capra et al., 2017); |
|  | *PARN* | 101112185 | 24 | 13,861,238-14,060,689 | 13,910,278 | - |
| TC | *NCKAP5L* | 101122265 | 3 | 136,480,240-136,510,941 | 136,501,556 | Fat metabolism in sheep (Liu et al., 2019);  Cold climate adaptation in sheep (Yudin and Larkin, 2023) |
|  | *SPATS2* | 101118688 | 3 | 136,720,265-136,875,197 | 136,789,194 | Fat tail deposition in sheep (Mastrangelo et al., 2019);  Fertility traits (high fecundity) in goats (^b^Wang et al., 2022) |
|  | *VAV2* | 101110137 | 3 | 2,186,976-2,364,531 | 2,189,666 | Pain response in sheep (Deng et al., 2018) |
|  | *LOC121819093*  (uncharacterised) | 121819093 | 3 | 129,030,690-129,073,357 | 129,053,073  129,060,248 | - |
|  | *PLEKHG7* | 101113743 | 3 | 129,075,938-129,158,031 | 129,127,634 | Pain response in sheep (Deng et al., 2018) |
|  | *CRADD* | 101115270 | 3 | 129,974,676-130,165,068 | 130,116,037 | - |
|  | *PLXNC1* | 101106582 | 3 | 130,420,049-130,575,490 | 130,503,901 | Mastitis resistance in sheep (Zuo et al., 2022) |
|  | *EXOC4* | 101107440 | 4 | 98,604,960-99,411,756 | 98,683,165 | Body weight in sheep (Zuo et al., 2022);  Muscle development traits in cattle (Bordbar et al., 2019) |
|  | *CREB5* | 101112976 | 4 | 68,869,477-69,308,814 | 69,167,211 | Fat tail development in sheep (Yuan et al., 2016) |
|  | *MAGI2* | 101102476 | 4 | 43,952,724-44,782,170 | 44,589,308 | - |
|  | *LOC121819400* | 121819400 | 4 | 51,242,635-51,508,560 | 51,467,401 | - |
|  | *ABCB1*  (*MDR1*) | 443367 | 4 | 33,893,569 33,995,231 | 33,893,167 | - |
|  | *CTNNA1* | 101112313 | 5 | 47,583,359-47,916,160 | 47,632,454 | Regulation of skeletal muscle development (inhibitor of myogenesis) in pigs (Zhao et al., 2011) |
|  | *WDFY3* | 101110676 | 6 | 100,188,709-100,469,517 | 100,209,430 | Carcass traits (bone weight) in cattle (Chang et al., 2018; Niu et al., 2021) |
|  | *PTPN13* | 101121266 | 6 | 102,189,370-102,416,348 | 102,345,798 | - |
|  | *EPB41L4A* | 101122032 | 7 | 1,455,363-1,742,418 | 1,610,113 | Fertility traits (litter size) in sheep (Gholizadeh and Esmaeili-Fard, 2022) |
|  | *DMGDH* | 101113594 | 7 | 10,041,506-10,112,577 | 10,040,798 | Fertility traits (litter weight) in pigs (Wu et al., 2018) |
|  | *UNC13C* | 101123470 | 7 | 53,791,928-54,498,557 | 54,300,572 | Heat tolerance in sheep (Aboul-Naga et al., 2022) |
|  | *TRPM7* | 100037693 | 7 | 57,824,962-57,915,989 | 57,901,067 | - |
|  | *OPRM1* | 101104758 | 8 | 78,545,349-78,602,834 | 78,554,080 | - |
|  | *CNKSR3* | 101105165 | 8 | 78,853,789-78,960,210  78,853,789-78,960,210 | 78,853,083  78,853,672 | - |
|  | *PRKN*  (*PARK2*) | 101113503 | 8 | 84,707,212-85,938,018 | 85,901,518 | Tail length in sheep (Tao et al., 2021);  Birth weight, postnatal growth traits (skeletal muscle development, regulation of metabolic pathways) in sheep (Mohammadi et al., 2020);  Meat production traits (head width, withers height) in cattle (Jourshari et al., 2023);  Lipid regulation in chickens (Pan et al., 2022) |
|  | *LOC101109626* | 101109626 | 10 | 71,057,752-71,241,310 | 71,182,852  71,182,854 | - |
|  | *LOC121820460* | 121820460 | 10 | 71,684,856-71,697,966 | 71,693,192 | - |
|  | *LOC105614203* | 105614203 | 10 | 71,700,097-71,883,325 | 71,818,877  71,818,878 | - |
|  | *PLD5* | 101119741 | 12 | 33,710,548-34,123,447 | 33,991,423 | Live body weight and body conformation traits in goats (Selionova et al., 2022) |
|  | *CNTNAP4* | 101118808 | 14 | 2,897,397-3,185,377 | 3,036,277 | Body conformation traits (body length) in sheep (Tao et al., 2020);  Fertility traits in cattle (Forutan et al., 2022);  Production traits in pigs (Lan et al., 2023) |
|  | *DENND5A* | 101123580 | 15 | 43,068,458-43,170,104 | 43,114,003  43,130,543 | - |
|  | *WT1* | 100101227 | 15 | 61,942,099-61,992,076 | 61,946,779  61,946,793 | Perirenal adipose deposition in Chinese indigenous sheep (Yuan et al., 2019) |
|  | *CCDC73* | 101122132 | 15 | 62,137,389-62,289,239 | 62,188,805 | - |
|  | *GRIA4* | 443274 | 15 | 1,498,196-2,166,450 | 1,989,301 | - |
|  | *LOC114118375* | 114118375 | 15 | 1,988,878-1,989,616 | 1,989,301 | - |
|  | *LOC101107200* | 101107200 | 15 | 66,102,557-66,103,954 | 66,103,828 | - |
|  | *KIAA1549L* | 101102174 | 15 | 62,809,295-63,117,626 | 63,004,206  63,004,296  63,005,660  63,005,838 | Body weight in sheep (Li et al., 2023) |
|  | *POLE* | 101116176 | 17 | 44,569,409-44,625,726 | 44,609,930 | - |
|  | *SEMA4B* | 101103596 | 18 | 20,891,221-20,933,797 | 20,931,232 | - |
|  | *SLCO3A1* | 101120608 | 18 | 14,259,305-14,633,889 | 14,465,097 | - |
|  | *BDKRB2* | 101120781 | 18 | 58,805,804-58,882,789 | 58,877,415 | Carcass traits in Santa Ines sheep (Cortez de Souza et al., 2022);  Skeletal muscle hypertrophy (muscle weight, body weight) in mice (de Picoli Souza et al., 2010; Verbrugge et al., 2018) |
|  | *CILK1* | 101107478 | 20 | 24,897,453-24,944,157 | 24,937,526 | Meat productivity traits (withers height) in sheep (Krivoruchko et al., 2023);  Growth retardation and pathologies of the spine in mice (Fu et al., 2019) |
|  | *PGK2* | 101102678 | 20 | 22,170,955-22,172,660 | 22,171,148  22,171,158 | - |
|  | *HIVEP1* | 101115835 | 20 | 43,479,638-43,615,643 | 43,582,171  43,582,173  43,583,072 | - |
|  | *LOC114110152* | 114110152 | 21 | 2,016,679-2,248,952 | 2,095,433 | - |
|  | *ELP2* | 101107058 | 23 | 21,308,232-21,363,536 | 21,350,358 | Body size traits (chest width) in sheep (Tao et al., 2021) |
|  | *ARHGAP28* | 101102444 | 23 | 40,279,522-40,421,813 | 40,293,253 | Fertility traits in sheep (Ghoreishifar et al., 2021; Moioli et al., 2017) |
|  | *LOC121817770* | 121817770 | 23 | 40,293,013-40,299,623 | 40,293,253 | - |
|  | *TENM3* | 101108282 | 26 | 12,367,699-12,996,592 | 12,402,338 | Meat production traits (chest width) in sheep (Krivoruchko et al., 2022);  Milk production traits (milk yield) in sheep (Sutera et al., 2019) |
| BL | *DPP10* | 101109248 | 2 | 180,472,640-181,242,813 | 180,804,649 | Carcass and pork traits (backfat thickness, lean meat percentage, ham lean meat percentage) in pigs (Jové-Juncà et al., 2024) |
|  | *LRP1B* | 101102124 | 2 | 168,072,439-170,263,357 | 168,805,458 | Muscle growth and development (birth weight) in goats (Zhang et al., 2021);  Birth weight in pigs (Wang et al., 2016) |
|  | *LOC101114268* | 101114268 | 5 | 31,833,567-31,835,249 | 31,833,572 | - |
|  | *HSD17B4* | 101114519 | 5 | 32,047,561-32,153,917 | 32,122,615 | Muscle development in pigs (Jo et al., 2016) |
|  | *ERBB2* | 780519 | 11 | 39,816,076-39,840,334 | 39,828,944 | - |
|  | *ADCY2* | 101102089 | 16 | 65,471,619-65,927,574 | 65,754,087 | Body weight in pigs (Ji et al., 2016);  Feed efficiency traits (residual feed intake) in Nellor cattle (Brunes et al., 2021);  Feed consumption traits (feed intake) in Japanes quail (Alboali et al., 2024) |
|  | *LOC121816974* | 121816974 | 17 | 68,255,711-68,266,893 | 68,255,923 | - |
|  | *PGPEP1L* | 101111665 | 18 | 7,506,808-7,577,350 | 7,566,767 | - |
|  | *GRM7* | 443520 | 19 | 18,572,632-19,515,318 | 19,445,400 | Body length in dogs (Sheet et al., 2021) |
|  | *CDYL* | 101120964 | 20 | 48,724,783-48,821,555 | 48,807,754 | - |
|  | *LOC105603900* | 105603900 | 20 | 48,926,527-48,958,695 | 48,941,872  48,945,850 | - |
|  | *SLC22A23* | 101122229 | 20 | 49,452,590-49,580,292 | 49,509,263 | Growth traits (live weight) in cattle (Zhang et al., 2018) |
| BW | *SPESP1* | 101106331 | 7 | 15,739,817-15,772,853 | 15,746,231  15,757,146 | - |
|  | *LOC101120092* | 101120092 | 10 | 2,249,108-2,363,880 | 2,352,274 | - |
|  | *SYT9* | 101106543 | 15 | 44,932,620-45,142,112 | 45,081,107 | - |
|  | *ADCY2* | 101102089 | 16 | 65,471,619-65,927,574 | 65,686,359  65,686,384  65,686,607  65,686,639 | Body weight in pigs (Ji et al., 2016);  Feed efficiency traits (residual feed intake) in Nellor cattle (Brunes et al., 2021);  Feed consumption traits (feed intake) in Japanes quail (Alboali et al., 2024) |
|  | *PCDH15* | 101115841 | 22 | 4,266,126-5,354,887 | 5,152,847 | Growth traits (weaning weight) in sheep (Abousoliman et al., 2021);  Body mass index in humans (Costa-Urruitia et al., 2019) |
|  | *ADD3* | 101117808 | 22 | 30,116,499-30,246,052 | 30,124,571 | - |
| nVERT | *FAM78B* | 101113028 | 1 | 118,168,754-118,262,819 | 118,211,889  118,212,195  118,213,342  118,213,462  118,213,561  118,214,025  118,214,333  118,214,391 | Endoparasite resistance in sheep (Ahbara et al., 2021);  Wool traits (mean fiber diameter) in sheep (Mohammadi et al., 2020) |
|  | *PPM1L* | 101105045 | 1 | 227,086,709-227,418,140 | 227,262,733 | - |
|  | *PALM2AKAP2* | 101114239 | 2 | 13,168,570-13,684,319 | 13,515,508 | Body size in pigs (Wu et al., 2020);  Body height in humans (Kim et al., 2010) |
|  | *SESTD1* | 101122080 | 2 | 130,867,094-131,007,612 | 130,920,325 | - |
|  | *LOC114114129* | 114114129 | 3 | 9,503,414-9,526,493 | 9,519,140  9,519,145 | - |
|  | *HMCN2* | 101101882 | 3 | 6,154,936-6,327,447 | 6,269,354 | Semen traits (gross motility) in sheep (Hodge et al., 2023) |
|  | *NCS1* | 101102136 | 3 | 6,363,404-6,419,146 | 6,415,846 | Semen traits (gross motility) in sheep (Hodge et al., 2023) |
|  | *ENPP3* | 101122795 | 8 | 57,075,090-57,164,843 | 57,147,610 | - |
|  | *ATG5* | 100913169 | 8 | 30,899,310-31,066,729 | 30,925,098 | - |
|  | *MAP3K5* | 101121018 | 8 | 61,835,832-62,064,330 | 62,035,863  62,037,560  62,038,029  62,038,030  62,038,593  62,039,086  62,039,283  62,040,246  62,042,066  62,049,583  62,056,179 | Body size traits (body height, body length, chest circumference, and cannon circumference) in Hu sheep (Yang et al., 2023);  Body size traits in sheep (Kominakis et al., 2017);  Feed efficiency in sheep and pigs (Alvarenga et al., 2017; Do et al. 2014) |
|  | *FIG4* | 101113245 | 8 | 27,684,956-27,863,956 | 27,793,072  27,797,205  27,797,217  27,797,490  27,797,829  27,797,921  27,800,377  27,808,974 | ALS (amyotrophic lateral sclerosis) in humans (Chow et al., 2009; Osmanovic et al., 2017) |
|  | *AFG1L* | 101116838 | 8 | 28,960,060-29,158,503 | 29,103,809 | Energy metabolism in South African Mutton Merino sheep (Liu et al., 2022b);  Energy metabolism in humans (Cesnekova et al., 2016) |
|  | *LOC101108923* | 101108923 | 8 | 29,713,581-29,881,084 | 29,870,405 | - |
|  | *KLF12* | 101122799 | 10 | 49,104,321-49,625,788 | 49,619,823 | Intramuscular fat deposition (preadipocyte differentiation and lipid accumulation) in goats (Du et al., 2021) |
|  | *LOC105602212* | 105602212 | 15 | 32,548,327-32,795,231 | 32,720,501 | - |
|  | *LOC114118558* | 114118558 | 15 | 77,728,670-77,731,885 | 77,731,683 | - |
|  | *TMEM135* | 101123334 | 21 | 7,803,992-8,073,204 | 7,918,557  7,920,188  7,923,411  7,947,533  7,979,688  7,980,282  7,982,923  7,986,570  7,986,580  7,986,594  7,986,713  7,986,801  7,987,120  7,987,735  7,988,213  7,988,311  7,988,543  7,988,802  7,989,109  7,989,136  7,989,311  7,989,337  7,989,919  7,990,535  7,991,220  7,991,421  7,991,536  7,991,543  7,991,784  7,992,634  7,992,638  7,993,402  7,994,269  7,995,259  7,995,300  7,995,444  7,998,054  7,998,503  7,998,651  7,998,666  7,998,714  7,998,769  7,998,803  7,999,739  7,999,819  7,999,820  7,999,989  8,000,573  8,000,772  8,000,863  8,001,353  8,001,415  8,001,550  8,001,559  8,001,692  8,003,739  8,007,731 | Bone growth and development (regulation of ossification) in chickens (Mohammadi et al., 2022) |
|  | *DISC1* | 101114672 | 25 | 3,815,272-4,249,748 | 3,816,061  3,817,185 | - |
| AXISD | *LOC101120030* | 101120030 | 1 | 50,432,486 50,772,209 | 50,496,903 | - |
|  | *DCBLD2* | 101107057 | 1 | 164,028,356-164,112,229 | 164,082,021 | - |
|  | *LOC114115358* | 114115358 | 1 | 23,581,773-24,515,480 | 24,229,121 | - |
|  | *NAALADL2* | 101111764 | 1 | 211,999,590-213,651,595 | 213,000,580 | - |
|  | *MFN1* | 101109233 | 1 | 208,498,977-208,534,821 | 208,514,899 | Growth traits (hucklebone width, hip width, height at sacrum, chest width and rump length) in cattle (Yao et al., 2022) |
|  | *PCOLCE2* (*PCPE2*) | 101104952 | 1 | 246,761,534-246,845,863 | 246,796,217 | Development of thoracolumbar vertebra and rib primordium in pigs (Li et al., 2021)  Cartilage development in humans (Steiglitz et al., 2002);  Bone formation and remodelling (van Gool et al., 2010) |
|  | *SERPINE2* | 101122004 | 2 | 226,127,034-226,195,095 | 226,151,076 | - |
|  | *PPP1R8* | 101105555 | 2 | 239,262,283-239,280,416 | 239,265,746 | - |
|  | *CAND1* | 101107775 | 3 | 152,640,674-152,676,244 | 152,673,014 | - |
|  | *PPM1H* | 101117068 | 3 | 156,769,622-157,065,084 | 156,964,159  156,964,207 | Mesenchymal cell differentiation (Shen et al., 2014) |
|  | *VAV2* | 101110137 | 3 | 2,186,976-2,364,531 | 2,250,348 | Limb-like skeletal structure development in zebrafish (Hawkins et al., 2021) |
|  | *ARNTL2* | 101112046 | 3 | 188,041,159-188,148,469 | 188,144,765 | - |
|  | *KCND2* | 101122690 | 4 | 86,317,435-86,875,141 | 86,655,230 | Growth traits (post weaning average daily gain) in Akkaraman sheep (Kizilaslan et al., 2022);  Growth traits (withers height) in neonatal sheep (Tao et al., 2020) |
|  | *CADPS2* | 101104231 | 4 | 88,341,260-88,915,993 | 88,439,308 | Chondrodysplasia in sheep (Zhao et al., 2012) |
|  | *IMMP2L* | 101113581 | 4 | 58,352,711-59,311,644 | 58,594,501 | - |
|  | *LOC105615213*  (*ZNF121*) | 105615213 | 5 | 12,108,046-12,180,788 | 12,159,841  12,159,847  12,162,966  12,163,718  12,163,757  12,163,763  12,163,767  12,163,801  12,163,821  12,163,865  12,164,051  12,166,984  12,167,506  12,167,579  12,167,698  12,167,862  12,168,519  12,168,573  12,168,676  12,168,709  12,168,727  12,169,564 | Intervertebral disc degeneration in humans (Yuan et al., 2020); |
|  | *ZNF846* | 114108666 | 5 | 12,188,791-12,208,290 | 12,195,088  12,198,834  12,198,927 | - |
|  | *LOC121819660* | 121819660 | 5 | 61,828,638-62,190,601 | 61,844,430  61,844,431  61,844,434 | - |
|  | *PTPN13* | 101121266 | 6 | 102,189,370-102,416,348 | 102,220,034 | - |
|  | *SIPA1L1* | 101122281 | 7 | 80,987,074-81,371,012 | 80,986,893 | - |
|  | *NRXN3* | 101116577 | 7 | 87,447,430-89,290,222 | 87,957,556 | - |
|  | *FOXN3* | 101114708 | 7 | 98,859,775-99,307,627 | 99,061,037 | - |
|  | *KATNBL1* | 101123043 | 7 | 26,598,640-26,654,337 | 26,638,348 | - |
|  | *RYR3* | 101111289 | 7 | 26,921,919-27,497,844 | 26,995,342  26,995,344 | Fat tail deposition in sheep (Xu et al., 2021) |
|  | *SCG3* | 101104667 | 7 | 56,807,343-56,844,831 | 56,809,481  56,809,494  56,810,547 | - |
|  | *FERMT2* | 101115796 | 7 | 63,644,760-63,717,761 | 63,655,947 | - |
|  | *PSMB1* | 101116408 | 8 | 91,713,291-91,734,719 | 91,733,839 | - |
|  | *TBP* | 100144760 | 8 | 91,734,827-91,751,971 | 91,733,839 | - |
|  | *AKAP12* | 101105509 | 8 | 76,104,452-76,213,902 | 76,194,731 | Growth traits (body weight, body length, chest depth, chest width, chest circumference, hip cross height, hip width, and cannon bone circumference) in Shaanbei white cashmere goats (Bai et al., 2021) |
|  | *ADGRB3* | 101109454 | 9 | 5,048,235-5,939,626 | 5,150,102 | - |
|  | *RALYL* | 101114878 | 9 | 90,633,037-91,454,224 | 90,805,166  90,991,981 | Growth and developmental traits (average daily gain) in Esme sheep (Yilmaz et al., 2021);  Birth weight in Alpine Merino sheep (Xiao et al., 2023) |
|  | *FAM135B* | 101122366 | 9 | 17,314,471-17,587,280 | 17,379,728 | - |
|  | *TOX* | 101117675 | 9 | 37,934,496-38,243,571 | 37,945,350  37,945,351 | - |
|  | *LOC101120092* | 101120092 | 10 | 2,249,108-2,363,880 | 2,254,150 | - |
|  | *LOC121820663* | 121820663 | 11 | 56,196,880-56,201,959 | 56,202,147 | - |
|  | *RIN2* | 101123061 | 13 | 38,930,046-39,134,879 | 38,987,595 | Meat production (DNA methylation in longissimus dorsi muscle) in sheep (Cao et al., 2017);  Growth and carcass traits (abdominal fat weight, fat width and hatching weigth traits) in chickens (Lin et al., 2020) |
|  | *MYO3A* | 101114632 | 13 | 25,859,778-26,031,351 | 25,954,034 | Growth traits in dromedaries (Sani et al., 2022);  Body mass index in humans (Graff et al., 2017) |
|  | *CENPN* | 101121445 | 14 | 7,187,597-7,214,327 | 7,214,573 | - |
|  | *EML2* | 101117612 | 14 | 52,993,777-53,018,367 | 53,009,229  53,009,239  53,009,299  53,009,300  53,009,330 | Embryonic development/ bone formation (thoracolumbar vertebral number) in Dezhou donkeys (^b^Sun et al., 2023) |
|  | *BBOX1* | 101115823 | 15 | 56,517,646-56,588,855 | 56,539,231 | - |
|  | *F2* | 100307041 | 15 | 75,592,821-75,606,996 | 75,591,837 | - |
|  | *RAB3C* | 101110089 | 16 | 20,634,185-20,932,905 | 20,825,864 | Regulation of exocytosis in mice (Geppert et al., 1994) |
|  | *ANKRD55* | 101112514 | 16 | 23,165,057-23,277,005 | 23,169,648 | - |
|  | *ARL15* | 101118640 | 16 | 24,959,594-25,422,539 | 25,174,145 | - |
|  | *MYO18B* | 101117620 | 17 | 65,700,568-65,942,641 | 65,759,510 | Skeletal muscle development in yaks (^a^Wu et al., 2022);  Skeletal muscle development (differentiation of myoblasts ) in mice (Tang et al., 2019) |
|  | *BDKRB2* | 101120781 | 18 | 58,805,804-58,882,789 | 58,831,930 | - |
|  | *EML1* | 101117881 | 18 | 62,362,079-62,556,237 | 62,450,557 | Carcass traits (tenderloin weight) in pigs (Zhou et al., 2023) |
|  | *ST8SIA2* | 101114980 | 18 | 13,928,934-14,001,817 | 13,963,476  13,963,488 | - |
|  | *LOC121817097*  (uncharacterised) | 121817097 | 18 | 26,985,952-27,033,503 | 27,008,705 | - |
|  | *OTUD7A* | 101120609 | 18 | 27,675,930-28,064,221 | 27,802,451 | - |
|  | *PEAK1* | 101104347 | 18 | 30,017,598-30,346,287 | 30,265,276 | - |
|  | *LRFN5* | 101103927 | 18 | 49,064,229-49,377,366 | 49,161,906 | - |
|  | *CHDH* | 101106116 | 19 | 47,169,305-47,258,495 | 47,190,274 | - |
|  | *PRKG1* | 443010 | 22 | 6,687,943-8,080,976 | 7,604,599 | - |
|  | *SORCS1* | 101116272 | 22 | 26,954,784-27,535,121 | 27,434,458  27,434,459  27,434,478 | Backfat thickness in Duroc pigs (Ding et al., 2022) |
|  | *CCDC172* | 101103108 | 22 | 36,284,799-36,340,152 | 36,286,860 | - |
|  | *HSPA12A* | 101120879 | 22 | 36,596,366-36,774,402 | 36,630,036  36,630,039  36,630,040  36,633,315  36,640,451  36,640,506  36,679,016 | Thermotolerance adaptation in sheep (Astuti et al., 2022);  Adaptation (heat tolerance) in cattle (Saravanan et al., 2021) |
|  | *SHTN1* | 101104358 | 22 | 36,804,280-36,917,681 | 36,834,378  36,849,769 | - |
|  | *SRRM3* | 105604868 | 24 | 34,727,844-34,781,579 | 34,748,328  34,759,103 | - |
|  | *RUSF1* | 101107249 | 24 | 28,042,029-28,057,744 | 28,050,186 | - |
|  | *CTNNA3* | 101123346 | 25 | 21,570,751-23,431,511 | 22,196,562  22,197,702  22,200,375  22,200,863  22,201,124  22,201,142  22,201,228  22,201,314  22,201,414  22,201,422  22,201,475  22,201,481  22,201,497  22,201,559  22,201,588  22,201,616  22,201,986  22,202,017  22,202,320  22,202,331  22,202,385 | Growth traits (body weight, body height, body length and chest circumference) in sheep (^a^Zhao et al., 2022);  Feed efficiency (average daily feed intake) in Hu sheep (Zhang et al., 2023);  Growth and developmental traits in Youzhou dark goats (^a^Sun et al., 2023);  Growth traits (chest depth) in Qinchuan cattle (Yu et al., 2023);  Embryonic development of skeletal muscles in chickens (^b^Wu et al., 2022) |
|  | *NRG3* | 101112537 | 25 | 35,919,548-37,160,225 | 36,585,999 | Angular limb deformity in Rambouillet sheep (Becker et al., 2023) |
|  | *CCSER2* | 101110815 | 25 | 38,308,913-38,468,877 | 38,348,893  38,348,895  38,349,928  38,354,020  38,357,712 | - |
|  | *JMJD1C* | 101123089 | 25 | 18,566,765-18,888,002 | 18,707,059 |  |
| BLCKV | *LOC121819842* | 121819842 | 1 | 78,681,800-78,899,891 | 78,881,013 | - |
|  | *CADM2* | 101120371 | 1 | 153,842,363-155,133,810 | 154,084,965  154,422,987 | Growth traits (body height) in Hu sheep (Jiang et al., 2021);  Growth traits (withers height and body length) in Chinese goats (Xu et al., 2020);  Body weight in chickens and mice (Wang et al., 2023; Yan et al., 2018) |
|  | *LOC114109980* | 114109980 | 1 | 237,189,033-237,191,485 | 237,188,043 | - |
|  | *LOC114109996* | 114109996 | 1 | 237,301,251-237,302,005 | 237,302,265  237,302,299  237,302,337  237,302,351 | - |
|  | *LOC101123603* | 101123603 | 2 | 22,816,449-22,854,782 | 22,819,462 | - |
|  | *RBKS* | 101120994 | 33 | 34,780,634-34,889,759 | 34,843,694  34,867,317  34,876,133  34,878,883  34,879,043  34,881,890 | - |
|  | *LCLAT1* | 101103884 | 3 | 37,298,612-37,490,607 | 37,478,853 | Fat tail deposition (lipid metabolism) in sheep (Zhang et al., 2020a) |
|  | *GALNT14* | 101104386 | 3 | 37,723,331-37,943,242 | 37,927,936 | - |
|  | *LRPPRC* | 101113138 | 3 | 80,305,231-80,404,271 | 80,362,067 | Body weight in Indian Sahiwal cattle (Kour et al., 2021) |
|  | *PRR16* | 101113754 | 5 | 30,798,472-31,079,455 | 30,895,176 | Birth weight in sheep (Abousoliman et al., 2021) |
|  | *ARHGAP24* | 101119904 | 6 | 100,964,948-101,542,349 | 101,174,658 | Growth traits (body condition score) in sheep (Lakhsassi et al., 2023);  Growth performance in pigs (Meng et al., 2017) |
|  | *FERMT2* | 101115796 | 7 | 63,644,760-63,717,761 | 63,648,796 | Body weight traits (fat synthesis, muscle development) in sheep (^b^Zhao et al., 2022);  Muscle growth and development (Suzuki et al., 2015; Suzuki et al., 2018) |
|  | *BCKDHB* | 101113939 | 8 | 7,103,498-7,372,809 | 7,330,435  7,337,271  7,337,719  7,337,988 | Angular limb deformity in Rambouillet sheep (Becker et al., 2021);  Fat metabolism in sheep (Yan et al., 2017) |
|  | *LRP12* | 101123640 | 9 | 72,962,315-73,051,379 | 73,003,262 | Osteoblast differentiation in mice (Gui et al., 2016) |
|  | *PSMD3* | 101105833 | 11 | 40,061,259-40,074,296 | 40,073,255 | - |
|  | *LOC114116936* | 114116936 | 11 | 6,820,921-6,867,287 | 6,867,339 | - |
|  | *ANKFN1* | 101122610 | 11 | 6,939,069-6,986,842 | 6,970,733  6,972,611 | - |
|  | *CEP112* | 101117261 | 11 | 61,472,298-61,785,295 | 61,669,853 | - |
|  | *TGFB2* | 554322 | 12 | 21,040,668-21,133,945 | 21,062,071 | Skeletal development (vertebrae, long bones, craniofacial bones) in mice (Baffi et al., 2004);  Skeletal abnormalities and developmental alterations in organs in mice (Sanford et al., 1997) |
|  | *KIF26B* | 101106609 | 12 | 30,912,133-31,429,837 | 30,920,857  30,981,544  31,063,397 | Osteogenic differentiation (post-traumatic heterotopic ossification) in humans and mice (Pickering et al., 2022);  Heterotopic ossification (Hatzikotoulas et al., 2019) |
|  | *SHCBP1L* | 101105346 | 12 | 63,392,402-63,431,076 | 63,405,408 | - |
|  | *RC3H1* | 101114887 | 12 | 54,641,533-54,715,548 | 54,714,209 | - |
|  | *TRIO* | 114118752 | 16 | 58,803,894-59,159,372 | 58,931,710  58,942,586 | - |
|  | *LOC114108841* | 114108841 | 17 | 70,597,127-71,220,068 | 70,899,743 | - |
|  | *ACTR8* | 101110005 | 19 | 47,135,144-47,162,952 | 47,149,209  47,149,295 | Lumbar spinal stenosis in humans (Jiang and Chen, 2021) |
|  | *LOC121817204*  (uncharacterised) | 121817204 | 19 | 9,567,410-9,572,755 | 9,569,732  9,570,064  9,571,273  9,571,288 | - |
|  | *LOC121817205*  (uncharacterised) | 121817205 | 19 | 9,572,745-9,604,499 | 9,575,672  9,577,932 | - |
|  | *DTNBP1* | 100145862 | 20 | 40,577,347-40,676,362 | 40,660,899  40,664,610 | Fat tail deposition (ratio of carcass weight to tail fat weight) in Hulun Buir sheep (^a^Zhang et al., 2019);  Body fat distribution in humans (Ma, Jia and Zhao, 2018) |
|  | *JARID2* | 101113542 | 20 | 40,676,953-40,907,561 | 40,696,096 | - |
|  | *CARMIL1* | 101116184 | 20 | 31,261,670-31,566,751 | 31,380,527 | Economic traits (average daily gain, backfat thickness) in Duroc pigs (^c^Wang et al., 2022) |
|  | *PRKG1* | 443010 | 22 | 6,687,943-8,080,976 | 6,996,983 | - |
|  | *SLC14A2* | 443267 | 23 | 45,298,765-45,794,210 | 45,769,422  45,792,498 | Conformation traits (back quality) in Danish pig breeds (Le et al., 2017);  Subcutaneous fat thickness in humans and pigs (Lee et al., 2011) |
|  | *PHLPP1* | 101119357 | 23 | 61,362,591-61,594,848 | 61,385,328 | Bone homeostasis in mice (Karkache et al., 2023);  Bone formation in mice (Mattson et al., 2019) |
|  | *SMG1* | 101111768 | 24 | 16,934,311-17,033,888 | 17,016,429  17,019,646  17,021,065 | - |
|  | *SIPA1L2* | 101112448 | 25 | 4,580,484-4,827,750 | 4,675,165  4,676,473 | Chondrogenic differentiation (^b^Chen et al., 2022) |
|  | *SLC16A9* | 101120546 | 25 | 14,657,594-14,737,960 | 14,657,948 | - |
|  | *CCDC6* | 101120801 | 25 | 14,797,518-14,907,581 | 14,817,273 | Osteoarthritis in mice (Butterfield et al., 2021) |
|  | *CTNNA3* | 101123346 | 25 | 21,570,751-23,431,511 | 21,989,171  21,993,572 | Growth traits (body weight, body height, body length and chest circumference) in sheep (^a^Zhao et al., 2022);  Skeletal muscle growth in chickens (^b^Wu et al., 2022) |
| WDGV | *MIGA1* | 101109140 | 1 | 54,243,541-54,320,287 | 54,300,617 | - |
|  | *ZMYM6* | 101108433 | 1 | 10,034,945-10,081,252 | 10,081,864 | - |
|  | *EFCAB14* | 101104868 | 1 | 21,225,842-21,264,609 | 21,256,729 | - |
|  | *ZYG11A* | 101116518 | 1 | 27,243,277-27,317,265 | 27,275,074  27,275,096 | - |
|  | *LOC105609512* | 105609512 | 1 | 27,272,948-27,274,243 | 27,275,074  27,275,096 | - |
|  | *NAALADL2* | 101111764 | 1 | 211,999,590-213,651,595 | 213,013,248 | - |
|  | *LOC105611838*  (uncharacterised) | 105611838 | 1 | 228,062,008-228,106,507 | 228,099,194 | - |
|  | *NBEAL1* | 101112883 | 2 | 205,106,564-205,265,401 | 205,131,027 | - |
|  | *PPP1R1C* | 101119792 | 2 | 127,678,781-127,801,107 | 127,692,838 | Development of muscularity (hindquarters, inner thighs, width of thighs, width of withers) in cattle (Doyle et al, 2020);  Muscle mass in mice (Kärst et al., 2011) |
|  | *MGAT4C* | 101108733 | 3 | 122,248,965-123,101,668 | 122,388,859 | Growth traits (weaning weight) in sheep (Abousoliman et al., 2021) |
|  | *LOC101106519* | 101106519 | 4 | 89,768,281-89,793,778 | 89,788,140 | - |
|  | *ORC5* | 101121088 | 4 | 46,826,080-46,901,627 | 46,854,217 | - |
|  | *PBX4* | 101104316 | 5 | 3,515,804-3,583,196 | 3,521,293  3,540,349 | - |
|  | *FBN2* | 101104991 | 5 | 23,263,268-23,492,427 | 23,463,970  23,466,230  23,466,924  23,467,672  23,467,932  23,470,111  23,470,357  23,471,004  23,478,539  23,478,801  23,478,891 | Abnormal bone phenotypes in mice (^a^Nistala et al., 2010);  Reduced bone formation in mice (^b^Nistala et al., 2010);  Skeletal abnormalities in humans (^a^Chen et al., 2022);  Bone mineral density in mice (Pei et al., 2019) |
|  | *LOC114114877*  (uncharacterised) | 114114877 | 5 | 44,238,438-44,436,629 | 44,272,673  44,272,762  44,273,927  44,279,776 | - |
|  | *DLGAP5* | 101118701 | 7 | 65,699,892-65,742,067 | 65,703,029 | - |
|  | *ELF1* | 101106441 | 10 | 11,535,981-11,651,712 | 11,612,621 | Growth and body conformation traits in pigs (Fernandez et al., 2012) |
|  | *MYO16* | 101115289 | 10 | 83,158,060-83,686,037 | 83,285,332 | Osteoarthritis in humans (Chou et al., 2012) |
|  | *PIBF1* | 101123131 | 10 | 48,141,509-48,370,729 | 48,233,775 | - |
|  | *LOC101119063* | 101119063 | 11 | 1,645,383-1,760,061 | 1,745,274  1,745,277  1,745,291  1,745,306 | - |
|  | *LOC106990700* | 106990700 | 12 | 467,278-469,832 | 470,777 | - |
|  | *LOC121820746* | 121820746 | 12 | 474,269-512,182 | 474,648  485,892 | - |
|  | *MMEL1* | 101120510 | 12 | 49,576,607-49,614,531 | 49,582,418  49,586,350  49,588,654 | - |
|  | *LOC114117613* | 114117613 | 13 | 62,555,003-62,565,336 | 62,558,710 | - |
|  | *CCDC7* | 101111568 | 13 | 19,358,118-19,618,532 | 19,587,840 | - |
|  | *LOC114118751*  (uncharacterised) | 114118751 | 16 | 56,138,725-56,256,110 | 56,236,591  56,239,435  56,240,183 | - |
|  | *ANKRD50* | 101123497 | 17 | 33,291,653-33,329,471 | 33,312,066  33,312,115 | - |
|  | *LOC114108841* | 114108841 | 17 | 70,597,127-71,220,068 | 71,061,733 | - |
|  | *FRMD5* | 101111053 | 18 | 53,322,619-53,648,164 | 53,478,649 | - |
|  | *CFAP20DC* | 101107905 | 19 | 42,457,451-42,718,329 | 42,652,034  42,653,998  42,654,017  42,654,022 | - |
|  | *LOC114109372*  (uncharacterised) | 114109372 | 19 | 42,752,247-42,769,639 | 42,763,245 | - |
|  | *CTDSPL* | 101114730 | 19 | 11,338,406-11,462,888 | 11,343,968 | Regulation of osteogenesis in humans (Wang et al., 2015) |
|  | *OVAR*  (*OVAR-I*) | 100171394 | 20 | 27,745,369-27,748,539 | 27,747,215  27,747,218 | - |
|  | *SLC14A2* | 443267 | 23 | 45,298,765-45,794,210 | 45,573,802 | Conformation traits in pigs (Le et al., 2017) |
|  | *LOC114110480*  (uncharacterised) | 114110480 | 23 | 45,566,179-45,604,632 | 45,573,802 | - |
|  | *CHRM3* | 443189 | 25 | 11,500,228-12,063,731 | 11,657,504 | - |
|  | *LOC114111062*  (uncharacterised) | 114111062 | 26 | 25,020,704-25,025,523 | 25,023,577 | - |
| FRC | *LOC105608331*  (uncharacterised) | 105608331 | 1 | 33,981,029-34,046,864 | 34,004,131 | - |
|  | *FGGY* | 101104022 | 1 | 34,046,935-34,672,433 | 34,304,551  34,493,962 | Skeletal muscle atrophy (inhibition of muscle cell differentiation) in mice (Smith et al., 2021) |
|  | *LOC114115024*  (uncharacterised) | 114115024 | 1 | 34,493,787-34,495,430 | 34,493,962 | - |
|  | *LOC101121563*  (*CYP2J2*) | 101121563 | 1 | 34,806,29034,837,200 | 34,820,819  34,820,894  34,821,762  34,821,777  34,823,379  34,823,443  34,824,308  34,824,323  34,824,332  34,829,118  34,829,119  34,829,871  34,829,875  34,831,004  34,834,161  34,834,618  34,834,629  34,835,875  34,835,894  34,835,916 | Bioactivation of vitamin D in humans (Aiba et al., 2006);  Arachidonic acid metabolism (Zordoky and El-Kadi, 2010) |
|  | *LOC101121820* | 101121820 | 1 | 34,844,469-34,880,255 | 34,844,645  34,845,456 | - |
|  | *PATJ* | 101106044 | 1 | 36,970,640-37,361,094 | 37,280,946 | - |
|  | *LINGO2* | 101112027 | 2 | 96,720,020-98,236,892 | 97,192,600  97,192,638  97,192,650  97,192,829  97,231,236 | Body size traits (heart size) in cattle (An et al., 2020);  Body mass in humans (Rask-Andersen et al., 2015);  Craniofacial abnormalities (macrocephaly, hypertelorism) in humans (Koufaris et al., 2015) |
|  | *FTCDNL1*  (*FONG*) | 101102297 | 2 | 202,169,511-202,264,929 | 202,168,647  202,168,798  202,168,950  202,169,266  202,169,994  202,170,388  202,171,509  202,175,364  202,177,844  202,180,471 | Osteoporosis in humans (Kou et al., 2011; Lu et al., 2015) |
|  | *SPATS2L* | 101103625 | 2 | 202,606,813-202,791,134 | 202,733,488  202,737,119 | Femoral head separation in chickens (Goldoni et al., 2022) |
|  | *ZNF804A* | 101116539 | 2 | 124,682,478-125,033,650 | 124,928,802  124,935,669 | Fractures in racehorses (Blott et al., 2014) |
|  | *MTPN* | 101102640 | 4 | 101,182,968-101,256,510 | 101,201,511 | Skeletal muscle development and growth in sheep, cattle and pigs (Bordbar et al., 2020; Pasandideh et al., 2020; Wang et al., 2012) |
|  | *SLC35B4* | 101120568 | 4 | 99,611,930-99,652,026 | 99,640,411 | - |
|  | *LOC106991103* | 106991103 | 4 | 99,634,968-99,642,061 | 99,640,411 | - |
|  | *COBL* | 101106517 | 4 | 5,361,674-5,667,161 | 5,485,279 | - |
|  | *PGM2* | 101111285 | 6 | 57,862,879-57,899,271 | 57,881,306 | - |
|  | *LOC105613069* | 105613069 | 7 | 21,990,625-21,992,785 | 21,989,921 | - |
|  | *LOC121820150*  (uncharacterised) | 121820150 | 8 | 12,266,228-12,292,907 | 12,283,481 | - |
|  | *TOM1L1* | 101120591 | 11 | 4,877,532-4,938,111 | 4,892,837 | - |
|  | *STK35* | 101114546 | 13 | 52,705,017-52,746,695 | 52,741,920 | - |
|  | *TRIM66* | 101102263 | 15 | 43,588,445-43,648,384 | 43,595,519  43,595,544  43,595,561  43,595,576  43,595,590  43,595,641 | Osteosarcoma carcinogenesis in humans and mice (Chen et al., 2015) |
|  | *SOX6* | 101116170 | 15 | 35,563,095-36,278,876 | 35,739,308  36,187,649  36,187,652  36,245,300 | Bone mineral density (osteoporosis) in humans (Yang et al., 2011);  Osteoblast development (osteoporosis) in humans (Hsu et al., 2009);  Chondrogenesis and cartilage formation (Dy et al., 2010; Ikeda et al., 2005; Lefebvre et al., 1998; Lefebvre et al., 2001; Smits et al., 2001; Uusitalo et al., 2001); |
|  | *EHF* | 100913164 | 15 | 63,998,618-64,041,973 | 64,042,933 | - |
|  | *VPS37C* | 101122144 | 21 | 34,823,115-34,852,724 | 34,831,922 | Number of cervical vertebrae in ducks (Xu et al., 2022) |

^1^No function: no function relevant to the study.

**References**

Aboul-Naga AM, Alsammam AM, El Allali A, Elshafie MH, Abdelal ES, Abdelkhalek TM, Abdelsabour TH, Mohamed LG, Hamwieh A (2022) Genome-wide analysis identified candidate variants and genes associated with heat stress adaptation in Egyptian sheep breeds. Front Genet 13. https://doi.org/10.3389/fgene.2022.898522

Abousoliman I, Reyer H, Oster M, Murani E, Mohamed I, Wimmers K (2021) Genome-wide analysis for early growth-related traits of the locally adapted Egyptian Barki sheep. Genes 12. https://doi.org/10.3390/genes12081243

Ahbara AM, Rouatbi M, Gharbi M, Rekik M, Haile A, Rischkowsky B, Mwacharo JM (2021) Genome‑wide insights on gastrointestinal nematode resistance in autochthonous Tunisian sheep. Scientific Reports 11. https://doi.org/10.1038/s41598-021-88501-3

Aiba I, Yamasaki T, Shinki T, Izumi S, Yamamoto K, Yamada S, Terato H, Ide H, Ohyama Y (2006) Characterization of rat and human CYP2J enzymes as Vitamin D 25-hydroxylases. Steroids 71:849-856. https://doi.org/10.1016/j.steroids.2006.04.009

Ajima R, Akazawa H, Kodama M, Takeshita F, Otsuka A, Kohno T, Komuro I, Ochiya T, Yokota J (2008) Deficiency of Myo18B in mice results in embryonic lethality with cardiac myofibrillar aberrations. Genes to Cells 13:987-999. https://doi.org/10.1111/j.1365-2443.2008.01226.xAlboali et al., 2024

Alvarenga AB (2017) Feed efficiency traits in Santa Inês sheep under genomic approaches. Universidade de São Paulo. https://doi.org/10.11606/D.11.2018.tde-20032018-160145

An B, Xu L, Xia J, Wang X, Miao J, Chang T, Song M, Ni J, Xu L, Zhang L, Li J, Gao H (2020) Multiple association analysis of loci and candidate genes that regulate body size at three growth stages in Simmental beef cattle. BMC genetics 21:1-11. https://doi.org/10.1186/s12863-020-0837-6

Astuti PK, Ilie DE, Gavojdian D, Wanjala G, Badaoui B, Ohran H, Pasic-Juhas E, Bagi Z, Jávor A, Kusza S (2022) Validation of SNP markers for thermotolerance adaptation in Ovis aries adapted to different climatic regions using KASP-PCR technique. Scientific Reports 12. https://doi.org/10.1038/s41598-022-26909-1

Baffi MO, Slattery E, Sohn P, Moses HL, Chytil A, Serra R (2004) Conditional deletion of the TGF-β type II receptor in Col2a expressing cells results in defects in the axial skeleton without alterations in chondrocyte differentiation or embryonic development of long bones. Developmental Biology 276:124-142. https://doi.org/10.1016/j.ydbio.2004.08.027

Bai Y, Yuan R, Luo Y, Kang Z, Zhu H, Qu L, Lan X, Song X (2021) Exploration of genetic variants within the goat A-Kinase anchoring protein 12 (AKAP12) gene and their effects on growth traits. Animals 11. https://doi.org/10.3390/ani11072090

Becker GM, Stegemiller MR, Schauer CSS, Stewart WCC, Murdoch BM (2021) PSIV-16 Genome-wide association study of Rambouillet rams with angular limb deformities. Journal of Animal Science 99:301. https://doi.org/10.1093/jas/skab235.554

Becker GM, Shira KA, Woods JL, Khilji SF, Schauer CS, Webb BT, Stewart WC, Murdoch BM (2023) Angular limb deformity associated with TSPAN18, NRG3 and NOVA2 in Rambouillet rams. Scientific Reports 13. https://doi.org/10.1038/s41598-023-43320-6

Bera TK, Liu XF, Yamada M, Gavrilova O, Mezey E, Tessarollo L, Anver M, Hahn Y, Lee B, Pastan I (2008) A model for obesity and gigantism due to disruption of the Ankrd26 gene. PNAS 105:270-275. https://doi.org/10.107/pnas.0710978105

Blott SC, Swineburne JE, Sibbons C, Fox-Clipsham LY, Helwegen M, Hillyer L, Parkin TDH, Newton JR, Vaudin M (2014) A genome-wide association study demonstrates significant genetic variation for fracture risk in Thoroughbred racehorses. BMC Genomics 15. https://doi.org/10.1186/1471-2164-15-147

Bordbar F, Jensen J, Zhu B, Wang Z, Xu L, Chang T, Xu L, Du M, Zhang L, Gao H, Xu L, Li J (2019) Identification of muscle-specific candidate genes in Simmental beef cattle using imputed next generation sequencing. PLoS One 14. https://doi.org/10.1371/journal.pone.0223671

Bordbar F, Jensen J, Du M, Abied A, Gu W, Xu L, Gao H, Zhang L, Li J (2020) Identification and validation of a novel candidate gene regulating net meat weight in Simmental beef cattle based on imputed next‐generation sequencing. Cell Proliferation 53, e12870. https://doi.org/10.1111/cpr.12870

Brunes LC, Baldi F, Lopes FB, Lôbo RB, Espigolan R, Costa MFO, Stafuzza NB, Magnabosco CU (2021) Weighted single-step genome-wide association study and pathway analyses for feed efficiency traits in Nellore cattle. J Anim Breed Genet 138:23-44. https://doi.org/10.1111/jbg.12496

Butterfield NC, Curry KF, Steinberg J, Dewhurst H, Komla-Ebri D, Mannan NS., Adoum AT, Leitch VD, Logan JD, Waung JA, Ghirardello E, Southam L, Youlten SE, Wilkinson JM, McAninch EA, Vancollie VE, Kussy F, White JK, Lelliot CJ, Adams DJ, Jaques R, Bianco AC, Boyde A, Zeggini E, Croucher PI, Williams GR, Bassett JD (2021) Accelerating functional gene discovery in osteoarthritis. Nature communications 12. https://doi.org/10.1038/s41467-020-20761-5

Cai Z, Guldbrandtsen B, Lund MS, Sahana G (2018) Dissecting closely linked association signals in combination with the mammalian phenotype database can identify candidate genes in dairy cattle. BMC Genet 19. https://doi.org/10.1186/s12863-019-0717-0

Cao Y, Jin HG, Ma HH, Zhao ZH (2017) Comparative analysis on genome-wide DNA methylation in longissimus dorsi muscle between Small Tailed Han and Dorper×Small Tailed Han crossbred sheep. Asian-Australasian Journal of Animal Sciences 30. https://doi.org/10.5713/ajas.17.0154

Capra E, Toschi P, Del Corvo M, Lazzari B, Scapolo PA, Loi P, Williams JL, Stella A, Ajimone-Marsan P (2017) Genome-wide epigenetic characterization of tissues from three germ layers isolated from sheep foetuses. Front Genet 8. https://doi.org/10.3389/fgene.2017.00115

Cesnekova J, Rodinova M, Hansikova H, Houstek J, Zeman J, Stiburek L (2016) The mammalian homologue of yeast Afg1 ATPase (lactation elevated 1) mediates degradation of nuclear-encoded complex IV subunits. Biochemical Journal 473:797-804. https://doi.org/10.1042/BJ20151029

Chang T, Xia J, Xu L, Wang X, Zhu B, Zhang L, Gao X, Chen Y, Li J, Gao H (2018) A genome-wide association study suggests several novel candidate genes for carcass traits in Chinese Simmental beef cattle. Anim Genet 49:312-316. https://doi.org/ 10.1111/age.12667

Chen W, Wang M, Zhang Z, Tang H, Zuo X, Meng X, Xiong M, Zhou F, Liang B, Dai F, Fang J, Gao J, Zhu J, Zhu Y, Wan H, Wang M, Chan S, Sun L (2015) Replication the association of 2q32. 2–q32. 3 and 14q32. 11 with hepatocellular carcinoma. Gene 561:63-67. https://doi.org/10.1016/j.gene.2015.02.006

Chen J, Xiang Q, Xiao X, Xu B, Xie H, Wang H, Yang M, Liu S (2022a) Carrying both COL1A2 and FBN2 gene heterozygous mutations results in a severe skeletal clinical phenotype: an afected family. BMCMedical Genomics 15. https://doi.org/10.1186/s12920-022-01296-8

Chen T, Peng Y, Hu W, Shi H, Li P, Que Y, Giu J, Gao B, Zhou H, Chen Y, Zhu Y, Li S, Liang A, Gao W, Huang D (2022b) Irisin enhances chondrogenic differentiation of human mesenchymal stem cells via Rap1/PI3K/AKT axis. Stem Cell Research & Therapy 13. https://doi.org/10.1186/s13287-022-03092-8Chou et al., 2012

Chow CY, Landers JE, Bergren SK, Sapp PC, Grant AE, Jones JM, Everett L, Lenk GM, McKenna-Yasek DM, Weisman LS, Figlewicz D, Brown RH, Meisler MH (2009) Deleterious variants of FIG4, a phosphoinositide phosphatase, in patients with ALS. The American Journal of Human Genetics 84:85-88. https://doi.org/10.1016/j.ajhg.2008.12.010

Cortez de Souza TC, Rocha da Cruz VA, Mourao GB, Pedrosa VB, Rovadoscki GA, Lehmann Coutinho LL, Ferreira de Camargo LF, Costa RB, Pinto de Carvalho GG, Pinto LFB (2022) Estimates of heritability and candidate genes for primal cuts and dressing percentage in Santa Ines sheep. Livestock Science 264. https://doi.org/10.1016/j.livsci.2022.105048

Costa-Urruitia P, Colistro V, Jimenéz-Osorio AS, Cárdenas-Hernández H, Solares-Tlapechco J, Ramirez-Alcántara M, Grenados J, de Jesús Ascencio-Montiel I, Rodríguez-Arellano E (2019) Genome-wide association study of body mass index and body fat in Mexican-Mestizo children. Genes 10. https://doi.org/10.3390/genes10110945de Picoli Souza et al., 2010

Deng X, Wang D, Wang S, Wang H, Zhou H (2018) Identifcation of key genes and pathways involved in response to pain in goat and sheep by transcriptome sequencing. Biol Res 51. https://doi.org/10.1186/s40659-018-0174-7

Deng T, Liang A, Liang S, Ma X, Lu X, Duan A, Pang C, Hua G, Liu S, Campanile G, Salzano A, Gasparrini B, Neglia G, Liang X, Yang L (2019) Integrative analysis of transcriptome and GWAS data to identify the hub genes associated with milk yield trait in buffalo. Front Genet 10. https://doi.org/10.3389/fgene.2019.00036

Ding R, Zhuang Z, Qiu Y, Ruan D, Wu J, Ye J, Cao L, Zhou S, Zheng E, Huang W, Wu Z, Yang J (2022) Identify known and novel candidate genes associated with backfat thickness in Duroc pigs by large-scale genome-wide association analysis. Journal of Animal Science 100. https://doi.org/10.1093/jas/skac012Do et al. 2014

Doyle JL, Berry DP, Veerkamp RF, Carthy TR, Evans RD, Walsh SW, Purfield, DC (2020) Genomic regions associated with muscularity in beef cattle differ in five contrasting cattle breeds. Genetics Selection Evolution 52:1-18. https://doi.org/10.1186/s12711-020-0523-1

Du Y, Emu Q, Zhu J, Lin Y (2021) miR-214-5p regulating differentiation of intramuscular preadipocytes in goats via targeting KLF12. Front Genet 12. https://doi.org/10.3389/fgene.2021.748629

Dy P, Smits P, Silvester A, Penzo-Méndez A, Dumitriu B, Han Y, de la Motte CA, Kingsley DM, Lefebvre V (2010) Synovial joint morphogenesis requires the chondrogenic action of Sox5 and Sox6 in growth plate and articular cartilage. Developmental Biology 341:346-359. https://doi.org/10.1016/j.ydbio.2010.02.024Fernandez et al., 2012

Forutan M, Engle B, Goddard ME, Hayes BJ (2022) A conditional multi-trait sequence GWAS of heifer fertility in tropically adapted beef cattle. Proceedings of 12th World Congress on Genetics Applied to Livestock Production (WCGALP) Technical and species orientated innovations in animal breeding, and contribution of genetics to solving societal challenges. Wageningen Academic Publishers 1106-1109. https://doi.org/10.3920/978-90-8686-940-4_262

Fu Z, Gailey CD, Wang EJ, Brautigan DL (2019) Ciliogenesis associated kinase 1: targets and functions in various organ systems. FEBS Letters 593:2990-3002. https://doi.org/10.1002/1873-3468.13600

Geppert M, Bolshakov VY, Siegelbaum SA, Takei K, De Camilli P, Hammer RE, Südhof TC (1994) The role of Rab3A in neurotransmitter release. Nature 369:493-497. https://doi.org/10.1038/369493a0

Gholizadeh M and Esmaeili-Fard (2022) Meta-analysis of genome-wide association studies for litter size in sheep. Theriogenology 180:103-112. https://doi.org/10.1016/j.theriogenology.2021.12.025

Ghoreishifar SM, Rochus CM, Moghaddaszadeh-Ahrabi S, Davoudi P, Ardestani SS, Zinovieva NA, Deniskove TE, Johansson AM (2021) Shared ancestry and signatures of recent selection in Gotland sheep. Genes 12. https://doi.org/10.3390/genes12030433

Goldoni I, Ibelli AMG, Fernandes LT, de Oliveira Peixoto J, Hul LM, Cantão ME, de Simoni Gouveia JJ, Ledur MC (2022) Comprehensive analyses of bone and cartilage transcriptomes evince ion transport, inflammation and cartilage development-related genes involved in chickens’ femoral head separation. Animals 12. https://doi.org/10.3390/ani12060788

Graff M, Scott R, Justice, AE, Young KL, Feitosa MF, Barata L et al. (2017) Genome-wide physical activity interactions in adiposity-A meta-analysis of 200,452 adults. PLoS genetics 13. https://doi.org/10.1371/journal.pgen.1006528

Gui Y, Duan Z, Qiu X, Tang W, Gober HJ, Li D, Wang L (2016) Multifarious effects of 17-β-estradiol on apolipoprotein E receptors gene expression during osteoblast differentiation in vitro. BioScience Trends 10:54-66. https://doi.org/10.5582/bst.2016.01006

Hatzikotoulas K, Pickering GA, Clark MJ, Felix-Ilemhenbhio F, Kocsy K, Simpson J, MacInnes SJ, Koprulu M, Southam L, Bellantuono I, Baidžajevas K, Young DA, Gartland A, Zeggini E, Kiss-Toth E, Wilkinson JM (2019) Genome-wide association and functional analyses identify CASC20 and KIF26B as target loci in heterotopic ossification. bioRxiv. https://doi.org/10.1101/845958

Hawkins MB, Henke K, Harris MP. (2021) Latent developmental potential to form limb-like skeletal structures in zebrafish. Celln184:899-911. https://doi.org/10.1016/j.cell.2021.01.003

Hodge MJ, de las Heras-Saldana S, Rindfleisch SJ, Stephen CP, Pant SD (2023) QTLs and candidate genes associated with semen traits in Merino sheep. Animals 13. https://doi.org/10.3390/ani13142286

Hsu TL, Tantoh DM, Chou YH, Hsu SY, Ho CC, Lung CC, Jan CF, Wang L, Liaw YP (2020) Association between osteoporosis and menopause in relation to SOX6 rs297325 variant in Taiwanese women. Menopause 27:887-892. https://doi.org/  10.1097/GME.0000000000001544

Ikeda T, Kawaguchi H, Kamekura S, Ogata N, Mori Y, Nakamura K, Ikegawa S, Chung UI (2005) Distinct roles of Sox5, Sox6, and Sox9 in different stages of chondrogenic differentiation. Journal of Bone and Mineral Metabolism 23:337-340. https://doi.org/10.1007/s00774-005-0610-y

Ji J, Zhou L, Guo Y, Huang L, Ma J (2016) Genome-wide association study identifies 22 new loci for body dimension and body weight traits in a White Duroc×Erhualian F2 intercross population. Asian-Australas J Anim Sci 30:1066-1073. https://doi.org/10.5713/ajas.16.0679

Jiang J, Cao Y, Shan H, Wu J, Song X, Jiang Y (2021) The GWAS analysis of body size and population verification of related SNPs in Hu sheep. Front Genet 12. https://doi.org/10.3389/fgene.2021.642552

Jiang X and Chen D (2021) The identification of novel gene mutations for degenerative lumbar spinal stenosis using whole-exome sequencing in a Chinese cohort. BMC Medical Genomics 14. https://doi.org/10.1186/s12920-021-00981-4

Jo JL, Hwang JH, Kwon SG, Park DH, Kim TW, Kang DG, Yu GE, Kim IS, Ha J, Kim CW (2016) Association between a non-synonymous HSD17B4 single nucleotide polymorphism and meat-quality traits in Berkshire pigs. Genet Mol Res 15. http://dx.doi.org/10.4238/gmr15048970

Jourshari MG, Shadparvar AA, Hossein-Zadeh NG, Rafeie F, Banabazi MH, Johansson AM (2023) Genome-wide association study on abdomen depth, head width, hip width, and withers height in native cattle of Guilan (Bos indicus). PLoS One 18. https://doi.org/10.1371/journal.pone.0289612

Jové-Juncà T, Crespo-Piazuelo D, González-Rodríguez O, Pascual M, Hernández-Banqué C, Reixach J, Quintanilla R, Ballester M (2024) Genomic architecture of carcass and pork traits and their association with immune capacity. Animal 18. https://doi.org/10.1016/j.animal.2023.101043

Kärst S, Cheng R, Schmitt AO, Yang H, De Villena FPM, Palmer AA, Brockmann GA (2011) Genetic determinants for intramuscular fat content and water-holding capacity in mice selected for high muscle mass. Mamm Genome 22:530–43. https://doi.org/10.1007/s00335-011-9342-6

Karkache IY, Molstad DH, Vu E, Jensen ED, Bradley EW (2023) Phlpp1 Expression in Osteoblasts Plays a Modest Role in Bone Homeostasis. JBMR plus 7. https://doi.org/10.1002/jbm4.10806

Kim JJ, Lee HI, Park T, Kim K, Lee JE, Cho NH, Shin C, Cho YS, Lee JY, Han BG, Yoo HW, Lee JK (2010). Identification of 15 loci influencing height in a Korean population. J. Hum. Genet. 55:27-31. https://doi.org/10.1038/jhg.2009.116

Kizilaslan M, Arzik Y, White SN, Piel LM, Cinar MU. (2022) Genetic parameters and genomic regions underlying growth and linear type traits in Akkaraman sheep. Genes 13. https://doi.org/10.3390/genes13081414

Kominakis A, Hager-Theodorides AL, Zoidis E, Saridaki A, Antonakos G, Tsiamis G (2017) Combined GWAS and ‘guilt by association’-based prioritization analysis identifes functional candidate genes for body size in sheep. Genet Sel Evol 49. https://doi.org/10.1186/s12711-017-0316-3

Kou I, Takahashi A, Urano T, Fukui N, Ito H, Ozaki K, Tanaka T, Hosoi T, Shiraki M, Inoue S, Nakamura Y, Kamatani N, Kubo M, Mori S, Ikegawa S (2011) Common variants in a novel gene, FONG on chromosome 2q33. 1 confer risk of osteoporosis in Japanese. PLoS One 6(5), e19641. https://doi.org/10.1371/journal.pone.0019641

Koufaris C, Papagregoriou G, Kousoulidou L, Moutafi M, Tauber M, Jouret B, Kieffer I, Deltas C, Tanteles GA, Anastasiadou V, Patsalis PC, Sismani, C (2015) Haploinsufficiency of the miR-873/miR-876 microRNA cluster is associated with craniofacial abnormalities. Gene 561:95-100. https://doi.org/10.1016/j.gene.2015.02.018

Kour A, Deb SM, Nayee N, Niranjan SK, Raina VS, MukherjeeA, Gupta ID, Patil CS (2023) Novel insights into genome-wide associations in Bos indicus reveal genetic linkages between fertility and growth. Animal Biotechnology 34:39-55. https://doi.org/10.1080/10495398.2021.1932520

Krivoruchko A, Sermyagin A, Saprikina T, Golovanova N, Kvochko A, Yatsyk O (2021) Genome wide associations study of single nucleotide polymorphisms with productivity parameters in Jalgin merino for identification of new candidate genes. Gene Reports 23. https://doi.org/10.1016/j.genrep.2021.101065

Krivoruchko AY, Yatsyk OA, Saprikina TY, Petukhova DD (2022) Genome-wide association study (GWAS) with productivity in Romanov sheep breed. Proceedings of the National Academy of Sciences of Belarus Agrarian Series 59:71-80. https://doi.org/10.29235/1817-7204-2021-59-1-71-80.

Krivoruchko AY, Zuev RV, Surov AI, Skokova AV, Kanibolotskaya AA, Likhovid AA, Yatsyk OA (2023) A genome-wide search of new meat-productivity candidate genes in a north Caucasian meat and wool sheep breed. Russ J Genet 59:483-492. https://doi.org/10.1134/S1022795423050095

Lakhssassi K, Meneses C, Sarto MP, Serrano M, Calvo JH (2023) Genome-wide analysis reveals that the cytochrome P450 family 7 subfamily B member 1 gene is implicated in growth traits in Rasa Aragonesa ewes. Animal 17. https://doi.org/10.1016/j.animal.2023.100975

Lan Q, Deng Q, Qi S, Zhang Y, Li Z, Yin S, Li Y, Tan H, Wu M, Yin Y, He J, Liu M (2023) Genome-wide association analysis identified variants associated with body measurement and reproduction traits in Shaziling pigs. Genes 14. https://doi.org/10.3390/genes14020522

Le TH, Christensen OF, Nielsen B, Sahana G. (2017) Genome-wide association study for conformation traits in three Danish pig breeds. Genetics Selection Evolution 49:1-12. https://doi.org/10.1186/s12711-017-0289-2

Lee KT, Byun MJ, Kang KS, Park EW, Lee SH, Cho S, Kim HY, Kim KW, Lee TH, Park JE, Park WC, Shin DH, Park HS, Kim H (2011) Neuronal genes for subcutaneous fat thickness in human and pig are identified by local genomic sequencing and combined SNP association study. PloS one 6(2), e16356. https://doi.org/10.1371/journal.pone.0016356

Lefebvre V, Li P, De Crombrugghe B (1998) A new long form of Sox5 (L-Sox5), Sox6 and Sox9 are coexpressed in chondrogenesis and cooperatively activate the type II collagen gene. The EMBO journal 17:5718-5733. https://doi.org/10.1093/emboj/17.19.5718

Lefebvre V, Behringer RR, De Crombrugghe B (2001) L-Sox5, Sox6 and Sox9 control essential steps of the chondrocyte differentiation pathway. Osteoarthritis and cartilage 9:69-S75. https://doi.org/10.1053/joca.2001.0447

Li J, Wang L, Yu D, Hao J, Zhang L, Adeola AC, Mao B, Gao Y, Wu S, Zhu C, Zhang Y, Ren J, Mu C, Irwin DM, Wang L, Hai T, Xie H, Zhang Y (2021) Single-cell RNA sequencing reveals thoracolumbar vertebra heterogeneity and rib-genesis in pigs. Genomics Proteomics Bioinformatics 19:423-436. https://doi.org/10.1016/j.gpb.2021.09.008

Li Y, Yang H, Guo J, Yang Y, Yu Q, Guo Y, Zhang C, Wang Z, Zuo P (2023) Uncovering the candidate genes related to sheep body weight using multi-trait genome-wide association analysis. Front Vet Sci 10. https://doi.org/10.3389/fvets.2023.1206383

Lin W, Ren T, Liu M, Zhang X (2021) Novel 61-bp indel of RIN2 is associated with fat and hatching weight traits in chickens. Front Genet 12. https://doi.org/10.3389/fgene.2021.672888

Liu JX, Wei X, Deng TY, Xie R, Han JL, Du LX, Zhao FP, Wang LX (2019) Genome-wide scan for run of homozygosity and identification of corresponding candidate genes in sheep populations. Acta Veterinaria et Zootechnica Sinica 50:1554-1566

Liu Q, Ren C, He X, Wang X, Xia Q, Chu M, Zhang Z (2022a) Screening of differentially expressed genes and miRNAs in hypothalamus and pituitary gland of sheep under different photoperiods. Genes 13. https://doi.org/10.3390/genes13061091

Liu Z, Bai C, Shi L, He Y, Hu M, Sun H, Peng H, Lai W, Jiao S, Zhao Z, Ma H, Yan S (2022b) Detection of selection signatures in South African Mutton Merino sheep using whole‐genome sequencing data. Anim Genet 53: 224-229. https://doi.org/10.1111/age.13173

Lu HF, Hung KS, Hsu YW, Tai YT, Huang LS, Wang YJ, Wong HSC, Hsu YH, Chang WC (2015) Association study between the FTCDNL1 (FONG) and susceptibility to osteoporosis. PLoS One 10. https://doi.org/10.1371/journal.pone.0140549

Ma L, Jia P, Zhao Z (2018) Splicing QTL of human adipose-related traits. Scientific reports 8. https://doi.org/10.1038/s41598-017-18767-z

Mastrangelo S, Moioli B, Ahbara A, Latairish S, Pportolano B, Pilla E, Ciani E (2019) Genome-wide scan of fat-tail sheep identifies signals of selection for fat deposition and adaptation. Anim Prod Sci 59:835-848. https://doi.org/10.1071/AN17753

Mattson AM, Begun DL, Molstad DH, Meyer MA, Oursler MJ, Westendorf JJ, Bradley EW (2019) Deficiency in the phosphatase PHLPP1 suppresses osteoclast-mediated bone resorption and enhances bone formation in mice. Journal of Biological Chemistry 294:11772-11784. https://doi.org/10.1074/jbc.RA119.007660

Meng Q, Wang K, Liu X, Zhou H, Xu L, Wang Z, Fang M (2017) Identification of growth trait related genes in a Yorkshire purebred pig population by genome-wide association studies. Asian-Australasian Journal of Animal Sciences 30:462-469. https://doi.org/10.5713/ajas.16.0548

Mohammadi H, Rafat SA, Shahrebabak HM, Shodja J, Moradi MH (2020) Genome-wide association study and gene ontology for growth and wool characteristics in Zandi sheep. Journal of Livestock Science and Technologies 8:45-55. https://doi.org/10.22103/jlst.2020.15795.1317

Mohammadi H, Khaltabadi Farahani AH, Moradi H, Haj I (2022) Genome wide association study based on gene-set enrichment analysis of growth traits in a chicken advanced intercross line. J Anim Sci Res 31. https://doi.org/10.22034/AS.2021.46637.1621

Moioli B, Steri R, Marchitelli C, Catillo G, Buttazzoni L (2017) Genetic parameters and genome-wide associations of twinning rate in a local breed, the Maremmana cattle. Animal 11:1660-1666. https://doi.org/10.1017/S1751731117000283

Nayak SS, Panigrahi M, Rajawat D, Ghildiyal K, Sharma A, Parida S, Bhushan B, Mishra BP, Dutt T (2023) Comprehensive selection signature analyses in dairy cattle exploiting purebred and crossbred genomic data. Mammalian Genome 34:615-631. https://doi.org/10.1007/s00335-023-10021-4

Nistala H, Lee-Arteaga S, Smaldone S, Siciliano G, Carta L, Ono RN, Sengle G, Arteaga-Solis E, Levasseur R, Ducy P, Sakai LY, Karsenty G, Ramirez F (2010) Fibrillin-1 and-2 differentially modulate endogenous TGF-β and BMP bioavailability during bone formation. Journal of Cell Biology 190:1107-1121. https://doi.org/10.1083/jcb.201003089

Nistala H, Lee-Arteaga S, Smaldone S, Siciliano G, Ramirez F (2010b) Extracellular microfibrils control osteoblast-supported osteoclastogenesis by restricting TGFβ stimulation of RANKL production. Journal of Biological Chemistry 285:34126-34133. https://doi.org/10.1074/jbc.M110.125328

Niu Q, Zhang T, Xu L, Wang T, Wang Z, Zhu B, Zhang L, Gao H, Song J, Li J, Xu L (2021) Integration of selection signatures and multi-trait GWAS reveals polygenic genetic architecture of carcass traits in beef cattle. Genomics 113:3325-3336. https://doi.org/10.1016/j.ygeno.2021.07.025

Osmanovic A, Rangnau I, Kosfeld A, Abdulla S, Janssen C, Auber B, Raab P, Preller M, Petri S, Weber RG (2017). FIG4 variants in central European patients with amyotrophic lateral sclerosis: a whole-exome and targeted sequencing study. European Journal of Human Genetic 25:324-331. https://doi.org/10.1038/ejhg.2016.186

Pan J, Purev C, Zhao H, Zhang Z, Wang F, Wendoule N, Qi G, Liu Y, Zhou H (2022) Discovery of exercise-related genes and pathway analysis based on comparative genomes of Mongolian originated Abaga and Wushen horse. Open Life Sciences 17:1269-1281. https://doi.org/10.1515/biol-2022-0487

Pasandideh M, Gholizadeh M, Rahimi‐Mianji G (2020) A genome‐wide association study revealed five SNPs affecting 8‐month weight in sheep. Anim Genet 5:973-976. https://doi.org/10.1111/age.12996

Pei YF, Liu L, Liu TL, Yang XL, Zhang H, Wei XT, Feng GJ, Hai R, Ran S, Lei Z (2019) Joint association analysis identified 18 new loci for bone mineral density. Journal of Bone and Mineral Research 34:1086-1094. https://doi.org/ 10.1002/jbmr.3681

Pickering GA, Felix-Ilemhenbhio F, Clark MJ, Kocsy K, Simpson J, Bellantuono I, Gartland A, Wilkinson JM, Hatzikotoulas K, Kiss-Toth E (2022) The Kinesin Gene KIF26B Modulates the Severity of Post-Traumatic Heterotopic Ossification. International Journal of Molecular Sciences 23. https://doi.org/10.3390/ijms23169203

Rabjohns EM, Rampersad RR, Ghosh A, Hurst K, Eudy AM, Brozowski JM, Lee HH, Ren Y, Mirando A, Gladman J, Bowser JL, Berg K, Wani S, Ralston SH, Hilton MJ, Tarrant TK (2023) Aged G protein-coupled receptor kinase 3 (Grk3)-deficient mice exhibit enhanced osteoclastogenesis and develop bone lesions analogous to human Paget’s disease of bone. Cells 12. https://doi.org/10.3390/cells12070981

Ramos Z, Garrick DJ, Blair HT, Vera B, Ciappesoni G, Kenyon PR (2023). Genomic regions associated with wool, growth and reproduction traits in Uruguayan Merino sheep. Genes 14. https://doi.org/10.3390/genes14010167

Rask-Andersen M, Almén MS, Lind L, Schiöth HB (2015) Association of the LINGO2-related SNP rs10968576 with body mass in a cohort of elderly Swedes. Molecular Genetics and Genomics 290:1485-1491. https://doi.org/10.1007/s00438-015-1009-7

Sanford LP, Ormsby I, Groot ACGD, Sariola H, Friedman R, Boivin GP, Cardell EL, Doetschman, T (1997) TGFβ2 knockout mice have multiple developmental defects that are non-overlapping with other TGFβ knockout phenotypes. Development 1242659-2670. https://doi.org/10.1242/dev.124.13.2659

Sani MB, Roudbari Z, Karimi O, Banabazi MH, Esmaeilkhanian S, Asadzadeh N, Harofte JZ, Naderi AS, Burger PA (2022) Gene-set enrichment analysis for identifying genes and biological activities associated with growth traits in dromedaries. Animals 12. https://doi.org/10.3390/ani12020184

Sani MB, Karimi O, Burger PA, Javanmard A, Roudbari Z, Mohajer M, Asadzadeh N, Harofteh JZ, Kazemi A, Naderi AS (2023) A genome-wide association study of morphometric traits in dromedaries. Vet Med Sci 9:1781-1790. https://doi.org/10.1002/vms3.1151

Saravanan KA, Panigrahi M, Kumar H, Parida S, Bhushan B, Gaur GK, Dutt T, Mishra BP, Singh RK (2021) Genomic scans for selection signatures revealed candidate genes for adaptation and production traits in a variety of cattle breeds. Genomics 113:955-963. https://doi.org/10.1016/j.ygeno.2021.02.009

Selionova M, Aibazov M, Mamontova T, Malorodov V, Sermyagin A, Zinovyeva N, Easa AA (2022) Genome-wide association study of live body weight and body conformation traits in young Karachai goats. Small Ruminant Research 216:106836. https://doi.org/10.1016/j.smallrumres.2022.106836

Sheet S, Kim JS, Ko MJ, Kim NY, Lim YJ, Park MR, Lee SJ, Kim JM, Oh SI, Choi BH (2021) Insight into the candidate genes and enriched pathways associated with height, length, length to height ratio and body-weight of Korean indigenous breed, Jindo dog using gene set enrichment-based GWAS analysis. Animals 11. https://doi.org/10.3390/ani11113136

Shen T, Sun C, Zhang Z, Xu N, Duan X, Feng XH, Lin X (2014) Specific control of BMP signaling and mesenchymal differentiation by cytoplasmic phosphatase PPM1H. Cell Research 24:727-741. https://doi.org/10.1038/cr.2014.48

Smith AL, Gjoka E, Izhar M, Novo KJ, Mason BC, De Las Casas A, Waddell DS (2021) FGGY carbohydrate kinase domain containing is expressed and alternatively spliced in skeletal muscle and attenuates MAP kinase and Akt signaling. Gene 800. https://doi.org/10.1016/j.gene.2021.145836

Smits P, Li P, Mandel J, Zhang Z, Deng JM, Behringer RR, de Crombrugghe B, Lefebvre V (2001) The transcription factors L-Sox5 and Sox6 are essential for cartilage formation. Developmental cell 1:277-290.

Steiglitz BM, Keene DR, Greenspan DS (2002) PCOLCE2 encodes a functional procollagen C-proteinase enhancer (PCPE2) that is a collagen-binding protein differing in distribution of expression and post-translational modification from the previously described PCPE1. Journal of Biological Chemistry, 277:49820-49830. https://doi.org/10.1074/jbc.M209891200

Sun Y, Li YH, Zhao CH, Teng J, Wang YH, Wang TQ, Shi XY, Liu ZW, Li HJ, Wang JJ, Wang WW, Ning C, Wang CF, Zhang Q (2023a) Genome-wide association study for numbers of vertebrae in Dezhou donkey population reveals new candidate genes. Journal of Integrative Agriculture 22:3159-3169. https://doi.org/10.1016/j.jia.2023.04.038

Sun X, Niu Q, Jiang J, Wang G, Zhou P, Li J, Chen C, Liu L, Xu L, Ren H (2023b) Identifying Candidate Genes for Litter Size and Three Morphological Traits in Youzhou Dark Goats Based on Genome-Wide SNP Markers. Genes 14. https://doi.org/10.3390/genes14061183

Sutera AM, Riggio V, Mastrangelo S, Di Gerlando R, Sardina MT, Pong-Wong R, Tolone M, Portolano B (2019) Genome-wide association studies for milk production traits in Valle del Belice sheep using repeated measures. Anim Genet 50:311-314. https://doi.org/10.1111/age.12789

Suzuki A, Pelikan RC, Iwata J (2015) WNT/β-catenin signaling regulates multiple steps of myogenesis by regulating step-specific targets. Molecular and Cellular Biology 35:1763-1776. https://doi.org/10.1128/MCB.01180-14

Suzuki A, Minamide R, Iwata J (2018) The role of acetyltransferases for the temporal-specific accessibility of β-catenin to the myogenic gene locus. Scientific Reports 8. https://doi.org/10.1038/s41598-018-32888-z

Tang X, Wang J, Zhou S, Zhou J, Jia G, Wang H, Xin C, Fu G, Zhang J (2019) miR‑760 regulates skeletal muscle proliferation in rheumatoid arthritis by targeting Myo18b. Molecular Medicine Reports 20:4843-4854. https://doi.org/10.3892/mmr.2019.10775

Tao L, He XY, Pan LX, Wang JW, Gan SQ, Chu MX (2020) Genome-wide association study of body weight and conformation traits in neonatal sheep. Anim Genet 51:336-340. https://doi.org/10.1111/age.12904

Tao L, Liu YF, Zhang H, Li HZ, Zhao FP, Wang FY, Zhang RS, Di R, Chu MX (2021) Genome-wide association study and inbreeding depression on body size traits in Qira black sheep (Ovis aries). Anim Genet 52:560-564. https://doi.org/10.1111/age.13099

Uusitalo H, Hiltunen A, Ahonen M, Gao TJ, Lefebvre V, Harley V, Kähäri VM, Vuorio E (2001) Accelerated up‐regulation of L‐Sox5, Sox6, and Sox9 by BMP‐2 gene transfer during murine fracture healing. Journal of Bone and Mineral Research 16:1837-1845. https://doi.org/10.1359/jbmr.2001.16.10.1837

van Gool S, Emons J, Leijten J, Decker E, Yu X, Sticht C van Houwelingen JC, Goeman JJ, Kleijburg C, Scherjon S, Gretz N, Wit JM, Rappold G, Karperien M (2010) Human fetal mesenchymal stem cells differentiating towards chondrocytes display a similar gene expression profile as growth plate cartilage. Regulation and modulation of growth 135

Verbrugge SAJ, Schönfelder M, Becker L, Nezhad FY, de Angelis MH, Wackerhage H (2018) Genes whose gain or loss-of-function increases skeletal muscle mass in mice: a systematic literature review- Front Physiol 9. https://doi.org/10.3389/fphys.2018.00553

Wang L and Wang Y (2012) Molecular characterization, expression patterns and subcellular localization of Myotrophin (MTPN) gene in porcine skeletal muscle. Molecular Biology Reports 39:2733-2738. https://doi.org/10.1007/s11033-011-1028-3

Wang Z, Xie Q, Yu Z, Zhou H, Huang Y, Bi X, Wang Y, Shi W, Sun H, Gu P, Fan X (2015) A regulatory loop containing miR-26a, GSK3β and C/EBPα regulates the osteogenesis of human adipose-derived mesenchymal stem cells. Scientific Reports 5. https://doi.org/10.1038/srep15280

Wang X, Liu X, Deng D, Yu M, Li X (2016) Genetic determinants of pig birth weight variability. BMC Genetics 17. https://doi.org/10.1186/s12863-015-0309-6

Wang S, Yang J, Li G, Ding R, Zhuang Z, Ruan D, Wu J, Yang H, Zheng E, Cai G, Wang X Wu Z (2022b) Identification of homozygous regions with adverse effects on the five economic traits of Duroc pigs. Frontiers in Veterinary Science 9. https://doi.org/10.3389/fvets.2022.855933

Wang JJ, Li ZD, Zheng LQ, Zhang T, Shen W, Lei CZ (2022c) Genome-wide detection of selective signals for fecundity traits in goats (Capra hircus). Gene 818. https://doi.org/10.1016/j.gene.2022.146221

Wang H, Zhao X, Wen J, Wang C, Zhang X, Ren X, Zhang J, Li H, Muhatai G, Qu L (2023) Comparative population genomics analysis uncovers genomic footprints and genes influencing body weight trait in Chinese indigenous chicken. Poultry Science 102. https://doi.org/10.1016/j.psj.2023.103031

Wu P, Yang Q, Wang K, Zhou J, Ma J, Tang Q, Jin L, Xiao W, Jiang A, Jiang Y, Zhu L, Li X, Tang G (2018) Single step genome-wide association studies based on genotyping by sequence data reveals novel loci for the litter traits of domestic pigs. Genomics 110:171-179. https://doi.org/10.1016/j.ygeno.2017.09.009

Wu F, Sun H, Lu S, Gou X, Yan D, Xu Z, Zhang Z, Qadri QR, Zhang Z, Wang Z, Chen Q, Li M, Wang X, Dong X, Wang Q, Pan Y (2020) Genetic diversity and selection signatures within Diannan small-ear pigs revealed by next-generation sequencing. Front Genet 11. https://doi.org/10.3389/fgene.2020.00733

Wu X, Chu M, Ma X, Pei J, Xiong L, Guo X, Liang C, Yan P (2022a) Genome-wide identification of RNA editing sites affecting muscle development in yak. Front Vet Sci 9. https://doi.org/10.3389/fvets.2022.871814

Wu P, Zhou K, Zhang J, Ling X, Zhang X, Zhang L, Li P, Wie Q, Zhang T, Wang X, Zhang G (2022b) Identification of crucial circRNAs in skeletal muscle during chicken embryonic development. BMC Genomics 23. https://doi.org/10.1186/s12864-022-08588-4

Xiao T, Li Y, Yue L, Lu Z, Yuan C, Song Y, Yang B, Liu J, Guo, T. (2023) Correlation of 20 Single-Nucleotide Polymorphisms with Weight and Wool Traits in Alpine Merino Sheep. Animals 14. https://doi.org/10.3390/ani14010127

Xu Z, Wang X, Zhang Z, An Q, Wen Y, Wang D, Liu X, Li Z, Lyu S, Li L, Wang E, Ru B, Xu Z, Huang Y (2020) Copy number variation of CADM2 gene revealed its association with growth traits across Chinese Capra hircus (goat) populations. Gene 741. https://doi.org/10.1016/j.gene.2020.144519

Xu S., Gao L, Shen M, Lyu F (2021) Whole-genome selective scans detect genes associated with important phenotypic traits in sheep (Ovis aries). Front Genet 12. https://doi.org/10.3389/fgene.2021.738879

Xu Y, Hu J, Fan W, Liu H, Zhang , Guo Z, Huang W, Liu X, Hou S (2022) Genome-wide association analysis reveals 6 copy number variations associated with the number of cervical vertebrae in Pekin ducks. Frontiers in Cell and Developmental Biology 10. https://doi.org/10.3389/fcell.2022.1041088

Yan XR, Shi T, Pan YY, Jing JJ, Cheng LF, Cao NX, Qiao LY, Liu WZ (2017) Regulation of the BCKDHB gene expression by miR-433-3p in ovine preadipocytes. Scientia Agricultura Sinica 50:4389-4397. https://doi.org/10.3864/j.issn.0578-1752.2017.22.015

Yan X, Wang Z, Schmidt V, Gauert A, Willnow TE, Heinig M, Poy MN (2018) Cadm2 regulates body weight and energy homeostasis in mice. Molecular metabolism 8:180-188. https://doi.org/10.1016/j.molmet.2017.11.010

Yang TL, Guo Y, Liu YJ, Shen H, Liu YZ, Lei SF, Li J, Tian Q, Deng HW (2011) Genetic variants in the SOX6 gene are associated with bone mineral density in both Caucasian and Chinese populations. Osteoporos Int 23:781-787. https://doi.org/10.1007/s00198-011-1626-x

Yang X, Wang W, Zhang D, Li X, Zhang Y, Zhao Y, Zhao L, Wang J, Xu D, Cheng J, Li W, Zhou B, Lin C, Zeng X, Zhai R, Ma Z, Liu J, Cui P, Zhang X (2023) Genetic polymorphism of the ovine MAP3K5 gene and its association with body size traits in Hu sheep of China. Arch Anim Breed 66:71-79. https://doi.org/10.5194/aab-66-71-2023

Yao Z, Li J, Zhang Z, Chai Y, Liu X, Li J, Huang Y, Li L, Huang W, Yang G, Chen F, Shi Q, Ru B, Lei C, Wang E, Huang Y (2022) The relationship between MFN1 copy number variation and growth traits of beef cattle. Gene 811. https://doi.org/10.1016/j.gene.2021.146071

Yilmaz O, Kizilaslan M, Arzik Y, Behrem S, Ata N, Karaca O, Elmaci C, Cemal I (2022) Genome‐wide association studies of preweaning growth and in vivo carcass composition traits in Esme sheep. Journal of Animal Breeding and Genetics 139:26-39. https://doi.org/10.1111/jbg.12640

Yu H, Yu S, Guo J, Cheng G, Mei C, Zan L (2023) Genome-Wide Association Study Reveals Novel Loci Associated with Body Conformation Traits in Qinchuan Cattle. Animals 13. https://doi.org/10.3390/ani13233628

Yuan Z, Liu Z, Kijas JW, Zhu C, Hu S, Ma X, Zhang L, Du L, Wang H, Wei C (2016) Selection signature analysis reveals genes associated with tail type in Chinese indigenous sheep. Anim Genet 48:55-66. https://doi.org/10.1111/age.12477

Yuan Z, Xiang R, Li W, Li F, Yue X (2019) Transcriptomic analyses revealed common tailed and perirenal adipose differentially expressed genes in four Chinese indigenous sheep breeds. Livestock Science 230. https://doi.org/10.1016/j.livsci.2019.103832

Yuan Q, Wang X, Liu L, Cai Y, Zhao X, Ma H, Zhang Y (2020) Exosomes derived from human placental Mesenchymal Stromal Cells carrying miR-4450 inhibitor alleviate intervertebral disc degeneration and ameliorate gait disturbances via up-regulation of ZNF121. Stem Cells and Development 29:1038-1058. https://doi.org/10.1089/scd.2020.0083

Yudin NS and Larkin DM (2023) Candidate genes for domestication and resistance to cold climate according to whole genome sequencing data of Russian cattle and sheep breeds. Vavilov Journal of Genetics and Breeding 27:463-470. https://doi.org/ 10.18699/VJGB-23-56

Zhang W, Xu L, Gao H, Wu Y, Gao X, Zhang L, Zhu B, Song Y, Bao J, Li J, Chen Y (2018) Detection of candidate genes for growth and carcass traits using genome-wide association strategy in Chinese Simmental beef cattle. Animal Production Science 58:224-233. http://dx.doi.org/10.1071/AN16165

Zhang Z, Ye M, Li Q, You Y, Yu H, Ma Y, Mei L, Sun X, Wang L, Yue W, Li R Li J, Zhang D (2019a) The schizophrenia susceptibility gene OPCML regulates spine maturation and cognitive behaviors through Eph-Cofilin signaling. Cell Reports https://doi.org/10.1016/j.celrep.2019.08.091

Zhang W, Xu M, Wang J, Wang S, Wang X, Yang J, Gao L, Gan S (2020a) Comparative tail fat transcriptome analysis of key genes and pathways activated in response to fat deposition in two sheep breeds with extreme fat-tail phenotype differences. https://doi.org/10.21203/rs.3.rs-33090/v1

Zhang L, Wang F, Gao G, Yan X, Liu H, Liu Z, Wang Z, He L, Lv Q, Wang Z, Wang R, Zhang Y, Li J, Su R (2021) Genome-wide association study of body weight traits in inner Mongolia Cashmere goats. Front Vet Sci 8. https://doi.org/10.3389/fvets.2021.752746

Zhang DY, Li XL, Li FD, Zhang XX, Zhao Y, Zhang, YK, Ma ZW, Tian HB, Weng XS, Wang WM (2023) Genome-wide association study identifies novel loci associated with feed efficiency traits in Hu lambs. Journal of Integrative Agriculture. https://doi.org/10.1016/j.jia.2023.10.011

Zhao X, Mo D, Li A, Gong W, Xiao S, Zhang Y, Qin L, Niu Y, Guo Y, Liu X, Cong P, He Z, Wang C, Li J, Chen Y (2011) Comparative analyses by sequencing of transcriptomes during skeletal muscle development between pig breeds differing in muscle growth rate and fatness. PLoS One 6. https://doi.org/10.1371/journal.pone.0019774

Zhao X, Onteru SK, Piripi S, Thompson KG, Blair HT, Garrick DJ, Rothschild MF (2012) In a shake of a lamb’s tail: using genomics to unravel a cause of chondrodysplasia in Texel sheep. Anim Genet 43:9-18. https://doi.org/10.1111/j.1365-2052.2011.02304.x

Zhao L, Li F, Yuan L, Zhang X, Zhang D, Li X, Zhang Y, Zhao Y, Song Q, Wang J, Zhou B, Cheng J, Xu D, Li W, Lin C, Wang W (2022a) Expression of ovine CTNNA3 and CAP2 genes and their association with growth traits. Gene 807. https://doi.org/10.1016/j.gene.2021.145949

Zhao L, Zhang D, Li X, Zhang Y, Zhao Y, Xu D, Cheng J, Wang J, Li W, Lin C, Yang X, Ma Z, Cui P, Zhang X, Wang W (2022b) Comparative proteomics reveals genetic mechanisms of body weight in Hu sheep and Dorper sheep. Journal of Proteomics 267. https://doi.org/10.1016/j.jprot.2022.104699

Zheng J, Deng T, Jiang E, Li J. Wijayanti D, Wang Y, Ding X, Lan X (2021) Genetic variations of bovine PCOS-related DENND1A gene identified in GWAS significantly affect female reproductive traits. Gene 802. https://doi.org/10.1016/j.gene.2021.145867

Zhou F, Quan J, Ruan D, Qiu Y, Ding R, Xu C, Ye Y, Cai G, Liu L, Zhang Z, Yang J, Wu Z, Zheng E (2023) Identification of Candidate Genes for Economically Important Carcass Cutting in Commercial Pigs through GWAS. Animals 13. https://doi.org/10.3390/ani13203243

Zordoky BN and El-Kadi AO (2010) Effect of cytochrome P450 polymorphism on arachidonic acid metabolism and their impact on cardiovascular diseases. Pharmacology & Therapeutics 125:446-463. https://doi.org/10.1016/j.pharmthera.2009.12.002

Zuo P, Zhang C, Gao Y, Zhao L, Guo J, Yang Y, Yu Q, Li Y, Wu Z, Yang H (2022) Genome-wide unraveling SNP pairwise epistatic effects associated with sheep body weight. Anim Biotechnol 34:3416-3427. https://doi.org/10.1080/10495398.2022.2152349
